# Spiking neural network decoders of finger forces from high-density intramuscular microelectrode arrays

**DOI:** 10.1038/s41467-026-74243-1

**Published:** 2026-06-24

**Authors:** Farah Baracat, Agnese Grison, Dario Farina, Giacomo Indiveri, Elisa Donati

**Affiliations:** 1 Institute of Neuroinformatics, University of Zürich and ETH Zürich, Zürich, Switzerland; 2 Department of Bioengineering, Imperial College London, London, UK

**Keywords:** Biomedical engineering, Motor neuron, Electrical and electronic engineering, Computer science

## Abstract

Assistive technologies for restoring naturalistic finger control require continuous and robust decoding of motor intent, with high accuracy and low latency. Here, we present a spike-based decoding framework that exploits the dynamics of spiking neural networks (SNNs) to efficiently process motor unit activity extracted from high-density intramuscular microelectrode arrays. Using this framework, we demonstrate simultaneous and proportional decoding of individual finger forces from motor unit spike trains during isometric contractions at 15% of maximum voluntary contraction. We systematically evaluated the properties of different SNN decoder configurations, comparing two possible input modalities: physiologically grounded motor unit spike trains and spike-encoded intramuscular EMG signals. Through this comparison, we determined the trade-offs between decoding accuracy, memory footprint, and robustness to input errors. Our results show that lean shallow SNNs are sufficient to decode finger-level motor intent with competitive accuracy, while operating, with minimal memory requirements and without the need for external pre-processing modules. This work provides a practical blueprint for integrating compact, low-power and low-latency SNNs into finger-level force decoding systems, demonstrating how the choice of input representation can be strategically tailored to meet application-specific requirements for accuracy, robustness, and memory efficiency.

## Abstract (web summary)

Decoding finger forces requires accurate and efficient estimation of motor intent. Here, the authors show that spiking neural networks enable precise, low-cost decoding from high-density intramuscular EMG using motor unit activity or directly encoded muscle signals.

## Introduction

Myoelectric systems interface with the peripheral nervous system by recording the electrical activity of skeletal muscles through electromyography (EMG), using either non-invasive surface electrodes or minimally invasive intramuscular electrodes^1–3^. Although EMG signals do not directly capture neural activity, they reflect the electrical activity of muscle fibers innervated by motor neurons and therefore provide an indirect estimate of the cumulative output of spinal motor circuits^4^. EMG-based systems have consequently been widely employed to infer user intent and control external devices such as neuroprostheses and exoskeletons^5–7^, as well as for applications in virtual and augmented environments^8^.

Among these applications, assistive and prosthetic systems impose particularly stringent requirements. Proportional and simultaneous control of individual fingers demands not only accurate decoding, but also low latency, minimal power consumption, and a small memory footprint. These constraints become critical in wearable or implantable systems, where increasing electrode density improves spatial and temporal resolution but amplifies data bandwidth, necessitating resource-efficient on-site processing^9^.

Conventional myoelectric decoding approaches typically rely on features extracted from EMG signals, such as time-domain statistics or frequency components, to estimate motor intent^10–12^. These methods have proven effective for classifying discrete movement classes and enabling proportional and simultaneous control of multiple Degrees-of-Freedoms (DoFs)^13–15^. However, reliance on global EMG features presents key limitations: signal features are often sensitive to electrode placement, skin-electrode impedance variations, and inter-subject variability, and they do not directly capture the underlying neural commands originating from spinal motor neurons^16–18^. As such, these global features offer only an indirect and coarse estimate of the intended neural drive to the muscles.

A promising alternative is to extract the precise discharge timings of individual motor units—the smallest functional elements of the motor system—using high-density EMG decomposition techniques^19–21^. Motor unit spike trains provide a direct, physiologically grounded, and temporally precise representation of the descending neural drive. Recent studies have demonstrated that decoding motor intent from motor unit activity outperforms traditional approaches based on global EMG features, particularly in tasks demanding high-resolution proportional control of individual fingers^22–24^.

However, a key limitation remains. Although motor unit activity is inherently discrete and event-driven, most decoding pipelines convert spike trains into continuous discharge rates using fixed-duration binning^22,24^. This rate conversion introduces latency and requires buffering spikes in memory, increasing computational overhead. In the context of real-time, resource-constrained assistive systems, this representational mismatch undermines the very advantages gained by accessing temporally precise neural activity.

A principled alternative emerges from the biological origin of movement. In the motor system, cortical commands are conveyed to spinal motor neurons as sequences of action potentials, which are integrated over time and translated into muscle contractions without intermediate rate conversion or external buffering^25^. Information is therefore transmitted, integrated, and acted upon directly in the form of discrete events. Spiking Neural Networks (SNNs) operate according to the same principles^26,27^. Rather than relying on continuous-valued activations, SNNs process streams of time-resolved spike events, where neurons and synapses jointly perform computation and state retention through their intrinsic temporal dynamics. This tight coupling between processing and memory reduces latency and computational complexity in real-time motor decoding applications^28^. Because computation is triggered only by events, unnecessary data movement and power consumption are minimized, making this paradigm particularly well-suited for neuromorphic hardware implementations^29,30^. The alignment between motor unit spike trains and event-driven computation offers, therefore, a natural framework for proportional decoding under assistive system constraints.

Despite this compatibility, the adoption of SNNs for proportional motor intent decoding remains limited. Existing studies have mostly focused on gesture classification from surface EMG, using spike-encoded global features and benchmark datasets^31–33^. For example, delta-modulated spike encodings have been deployed on neuromorphic hardware for low-power hand gesture recognition, while other works have explored alternative SNN topologies on datasets such as NinaPro^34^. However, these approaches target discrete gesture recognition rather than continuous, simultaneous force decoding. To date, only a single study has explored decomposed motor unit spike trains within an SNN framework, and it focused on gesture classification rather than proportional control^35^. Proportional decoding from intramuscular motor unit activity using SNNs, particularly under resource-constrained conditions, therefore, remains largely unexplored.

In this work, we address this gap by systematically investigating spike-based motor decoding for real-time assistive applications. Specifically, we compare classical and neuromorphic decoding pipelines through three key methodological advancements. First, leveraging recent advances in high-density intramuscular electromyography (HD-iEMG) decomposition that enable the identification of large motor unit populations^36–38^, we demonstrate proportional and simultaneous decoding of individual finger forces from motor unit spike trains recorded using implanted microelectrode arrays (MEAs). Second, we compare classical pipelines and shallow SNN architectures, explicitly evaluating decoding performance alongside memory footprint and computational cost. Third, recognizing that motor unit decomposition itself incurs substantial computational and memory overhead due to the need to store the separation matrices^39,40^, we evaluate a complementary event-based strategy that encodes filtered HD-iEMG signals into spike trains using adaptive leaky integrate-and-fire (ALIF) neurons. Although such representations lack explicit physiological correspondence to individual motor units, they provide a lightweight, hardware-compatible alternative for resource-constrained implementations^36–38^.

By comparing these decoding strategies, we expose the fundamental trade-offs between physiologically meaningful inputs and implementation efficiency. This comparison provides a principled basis for selecting decoding pipelines according to the constraints and objectives of assistive systems, where accuracy, latency, memory footprint, and computational cost must be jointly considered.

## Results

**Fig. 1:**
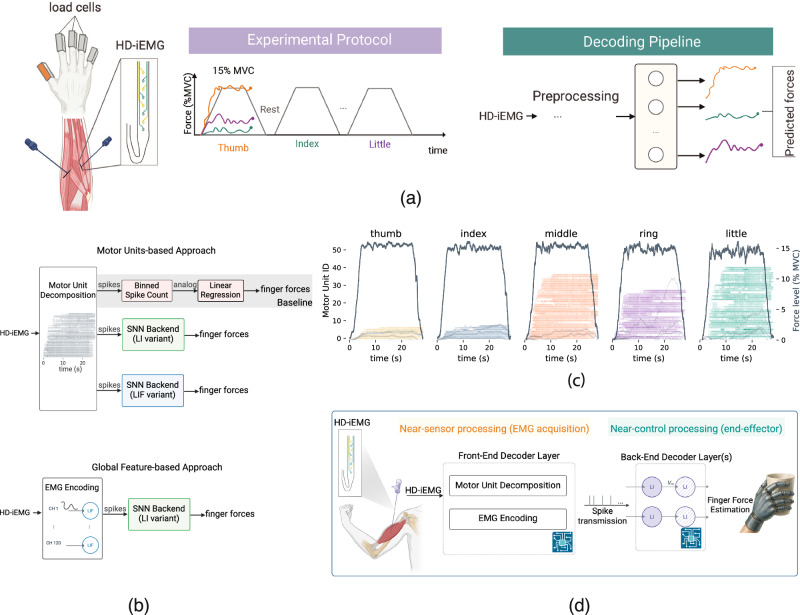
Force decoding from HD-iEMG using spike-based representations. **a** Experimental setup and protocol. HD-iEMG was recorded from forearm muscles using microelectrode arrays while subjects performed single-digit isometric contractions at 15% MVC. Individual finger forces were measured simultaneously using load cells. The decoding pipeline aimed to estimate the continuous force trajectories of all fingers. Created in BioRender. Baracat, F. (2026) https://BioRender.com/8ecqk7a. **b** Schematic of decoding architectures using either decomposed motor unit spike trains (top) or spike-encoded HD-iEMG signals (bottom). Motor unit spikes are processed through an LI layer or a cascade of LIF neurons followed by LI units. The baseline model applies linear regression to binned spike counts. Created in BioRender. Baracat, F. (2026) https://BioRender.com/q26yhw4. **c** Example task-specific motor unit activity decomposed from HD-iEMG and corresponding force output during isometric contractions across five finger tasks (Subject 1, repetition 2). Motor units were decomposed independently for each task; therefore, motor unit IDs (*y* axis) are not shared across the five finger tasks. **d** Conceptual overview of the proposed pipeline implementation for near-sensor spike-based processing and distal force estimation. Created in BioRender. Baracat, F. (2026) https://BioRender.com/v0qe9qv.

We demonstrate the potential of spike-based processing for decoding individual finger movements in isometric contraction at 15% maximal voluntary contraction (MVC), exploiting an HD-iEMG setup (Fig. 1a)^38^. We constructed two SNN architectures and investigated two possible input modalities derived from intramuscular EMG recordings: decomposed motor unit spike trains and direct spike-encoded representations (Fig. 1b). For both cases, the resulting input spikes are streamed into the SNNs composed of leaky integrate-and-fire (LIF) neurons and biophysically realistic synapses which leverage their intrinsic temporal dynamics to process continuous force trajectories, without requiring extra spike binning or stand-alone feature extraction stages.

We investigated two variants of the pipelines, distinguished by the readout neuron type of the decoder network. In the leaky readout variant, motor units were mapped to five leaky integrator (LI) units through dense feedforward synapses, with continuous force estimates for each finger read directly from their continuous-time real-value output. In the spiking variant, a first layer of five LIF neurons processed the motor unit spike trains, followed by a cascade of two LI layers connected one-to-one with the preceding units: the first transformed spike trains into continuous values, and the second applied temporal smoothing to yield the final force estimates. Hereafter, we refer to these two configurations as the LI and LIF configurations, respectively.

We conducted four experiments to: (1) benchmark the two SNN topologies against a conventional linear baseline for decoding finger forces from motor unit spike trains; (2) assess model robustness to spike omissions simulating decomposition errors; (3) compare decoding performance using spike-encoded HD-iEMG signals as an alternative to motor unit-based inputs; and (4) validate the pipeline on mixed-signal neuromorphic hardware.

### Streamed processing of motor units' spike trains with neurons and synapses

Three high-density intramuscular MEAs with a total of 120 channels were inserted within the forearm muscles of two healthy male participants as they performed sequential isometric flexion and extension tasks with individual fingers at 15% of their MVC. Motor unit spike trains were decoded from the recorded intramuscular HD-iEMG using a validated blind source separation algorithm designed for high-yield decomposition^37,38,41^. Decomposition was performed independently for each finger and movement direction, and the resulting spike trains were aggregated to form direction-specific input sets. For instance, all motor units identified during flexion trials of all fingers were combined to train the decoder. A total of 121 motor units were identified during finger flexion tasks and 196 during extension tasks for Subject 1. For Subject 2, 51 units were identified during flexion and 93 units during extension. An example of decomposed motor units' spike trains during finger extension tasks is shown in Fig. 1c.

**Fig. 2:**
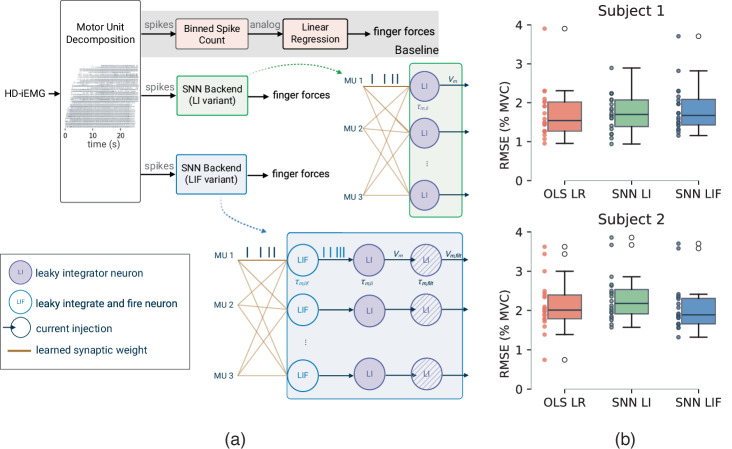
Comparison of motor unit-based decoder performance across a baseline linear regression model and SNN. **a** Schematic illustration of the two decoding pipelines: motor unit spike counts binned over fixed windows and decoded with an OLS LR (baseline), versus direct decoding from motor unit spike times with SNN backends. Created in BioRender. Baracat, F. (2026) https://BioRender.com/se6u1m4. **b** RMSE distributions for individual finger flexion and extension tasks, comparing the baseline model (spike count) to SNN decoders with leaky integrator (LI) and leaky integrate-and-fire (LIF) readout units. Points represent RMSE values for each repetition, finger task, and movement direction (*n* = 20). Box plots show the median (centre line), interquartile range (box; 25th–75th percentiles), and whiskers extending to the minimum and maximum values. Distributions are shown descriptively; quantitative comparisons are reported using effect sizes and confidence intervals in the Results.

In the first experiment, we compared SNN decoders to a conventional ordinary least-squares linear regression (OLS LR) baseline, as used in previous studies^23,24^. The overview schematic for the first experiment is illustrated in Fig. 2a. The baseline model received binned spike counts computed over 80 ms analysis windows with 50% overlap as input features (see “Baseline regression models”). In contrast, the SNN decoders operated directly on the motor unit spike trains, which were streamed into a dense layer of either spiking neurons or leaky integrator units. This first layer integrated input spikes continuously, as they arrived, without requiring explicit temporal segmentation (Fig. 1d).

All models were trained in a multi-output regression setting, simultaneously estimating the force trajectories of all five fingers from the pool of identified motor units associated with a given movement direction (flexion or extension). Performance metrics, summarized in Fig. 2b, report root mean squared error (RMSE) values averaged across two repetitions using cross-validation and computed over all finger tasks. Additional metrics, including coefficient of determination (R2) and mean absolute error (MAE), are summarized in Table 1 (see Table S1 for a detailed performance breakdown by movement direction). The baseline linear regression model achieved an average RMSE of 1.73 ± 0.29% MVC for Subject 1 and 2.13 ± 0.32% MVC for Subject 2. The LI configuration achieved 1.73 ± 0.23 and 2.31 ± 0.34% MVC, while the LIF configuration yielded 1.78 ± 0.27 and 2.12 ± 0.41 % MVC for Subjects 1 and 2, respectively. Both network variants achieved decoding performance comparable to the linear baseline model. To quantify these differences, we computed within-subject effect sizes defined as the mean difference in RMSE between the baseline OLS LR decoder and each SNN decoders (baseline-SNN), along with 95% bootstrap confidence intervals estimated at the repetition level. For Subject 1, the mean RMSE difference was 0.003 % MVC for the LI configuration (95% CI: [−0.25, 0.25] % MVC) and −0.05 % MVC for the LIF-based variant (95% CI: [−0.31, 0.21] % MVC). For Subject 2, the corresponding differences were −0.18% MVC for the LI variant (95% CI: [−0.64, 0.28] % MVC) and 0.01% MVC for the LIF variant (95% CI: [−0.44, 0.46] % MVC). In all cases, confidence intervals included zero, indicating that observed accuracy differences are small compared to repetition-level variability and do not support a consistent advantage of either model.

**Table 1:** Decoder performance across subjects, input features, and models (LR, RNNs, and SNNs)

Subject	Input type	Decoder Model	RMSE (% MVC)	MAE (% MVC)	*R*^2^
1	Motor unit spike count	**LR**	**1.73** ± **0.29**	**1.01** ± **0.14**	**0.88** ± **0.04**
	Motor unit spike train	RNN (vanilla)	2.02 ± 0.37	1.21 ± 0.19	0.85 ± 0.05
		RNN (GRU)	1.93 ± 0.28	1.29 ± 0.17	0.86 ± 0.04
		**SNN (LI)**	**1.73** ± **0.23**	**1.09** ± **0.13**	**0.89** ± **0.03**
		SNN (LIF)	1.78 ± 0.27	1.10 ± 0.21	0.88 ± 0.04
	Encoded HD-iEMG	SNN (LI)	2.76 ± 1.57	1.86 ± 1.05	0.66 ± 0.35
2	Motor unit spike count	LR	2.13 ± 0.32	1.36 ± 0.16	0.84 ± 0.05
	Motor unit spike train	RNN (vanilla)	2.74 ± 0.61	1.83 ± 0.45	0.73 ± 0.11
		RNN (GRU)	2.21 ± 0.48	1.50 ± 0.39	0.83 ± 0.08
		SNN (LI)	2.31 ± 0.34	1.64 ± 0.29	0.82 ± 0.05
		**SNN (LIF)**	**2.12** ± **0.41**	**1.46** ± **0.38**	**0.85** ± **0.06**
	Encoded HD-iEMG	SNN (LI)	2.91 ± 1.15	2.13 ± 0.86	0.70 ± 0.21

Metrics are averaged across fingers, directions, and 10 random initialization seeds for the SNN decoders and for the recurrent baselines (vanilla RNN and GRU). Bold values indicate the best-performing models (lowest mean RMSE) for each subject.

We next sought to isolate the temporal benefits of SNNs and to examine whether the mapping from decomposed motor units to finger forces is essentially linear. To this end, we compared the SNNs with lightweight Recurrent Neural Networks (RNNs) baselines, a vanilla RNN and a Gated Recurrent Unit (GRU) variant with matched parameter counts. These RNNs operated directly on the smoothed motor unit spike trains, obtained by convolving each spike train with an exponential kernel (time constant 10 ms) that mirrors the synaptic filtering in the SNNs, thereby eliminating the need for binning. This comparison serves two purposes. First, it evaluates whether the mapping from decomposed motor units to multi-finger forces requires nonlinear temporal processing or is sufficiently captured by a linear decoder. Second, it assesses whether the proposed SNNs extract and exploit temporal dependencies in the data on par with recurrent architectures explicitly designed for this role.

As summarized in Table 1 and detailed in Table S1, the recurrent baselines achieved decoding accuracy comparable to the SNNs, with subject- and architecture-dependent differences. For Subject 1, the vanilla RNN and GRU achieved average RMSE values of 2.02 ± 0.37 and 1.93 ± 0.28 % MVC, respectively, compared to 1.73 ± 0.23 % MVC for the SNN with a direct LI readout and 1.78 ± 0.27 % MVC for the LIF variant. The corresponding effect sizes also indicated small differences: the GRU exhibited a mean RMSE difference of 0.20 % MVC relative to the LI SNN (95% CI: [0.08, 0.32]) and 0.15 % MVC relative to the LIF variant (95% CI: [−0.01, 0.31]), while the vanilla RNN differences were smaller and not directionally conclusive. For Subject 2, the vanilla RNN achieved an average RMSE of 2.74 ± 0.61 % MVC, compared to 2.31 ± 0.34 % MVC (LI) and 2.12 ± 0.41 % MVC (LIF) for the SNNs. The associated effect sizes were negative and larger in magnitude, with mean differences of  − 0.54 % MVC for both LI and LIF topologies (95% CI: [ − 0.77,   − 0.31]), indicating poorer decoding accuracy of the vanilla RNN under matched-parameter constraints. In contrast, the GRU achieved 2.21 ± 0.48 % MVC, with mean differences close to zero relative to the SNNs and confidence intervals spanning zero. Overall, while some recurrent configurations exhibited modest subject-specific differences, effect sizes remained small relative to repetition-level variability and did not reveal a consistent accuracy advantage of recurrent architectures over the SNNs.

However, we must acknowledge two caveats to these outcomes. First, the limited amount of data available is likely constraining the performance of the RNNs. Second, because the size of the recurrent hidden state was restricted to match the parameter counts of the linear and SNN models, the RNNs were limited to a single recurrent layer with only five units. Such a compact RNN architecture may simply not have sufficient capacity to capture potential nonlinear structure in the data, even if such structure is present. Yet, this also points to a potential advantage: under matched-parameter conditions, SNNs may be less data-hungry than the conventional RNN architectures. Further validation on larger datasets is needed to conclusively assess this possibility. Nonetheless, decoding accuracy, although a critical measure of model performance, does not by itself capture the practical trade-offs associated with different decoding architectures.

**Fig. 3:**
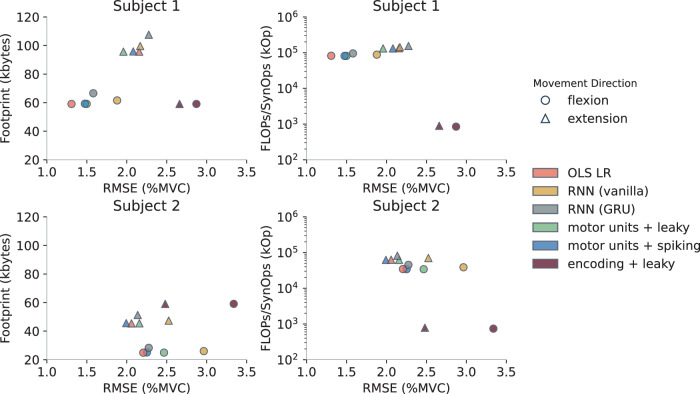
Trade-offs between decoding accuracy, memory footprint, and computational cost. Relationship between decoding accuracy, RMSE, expressed in % MVC, memory footprint, and computational cost for baseline and SNN decoding pipelines. Left panels report memory footprint (kbytes) as a function of RMSE, while right panels report computational cost, measured as the average FLOPs per sample for conventional pipelines and SynOp for SNN-based models, as a function of RMSE. Memory and computational metrics account for all stages of the decoding pipeline, including signal decomposition for motor-unit-based approaches and binning or convolutional preprocessing for baseline pipelines. Marker shape denotes movement direction (flexion or extension), and color indicates decoder architecture.

Figure 3 places these results in the broader context of memory footprint and computational cost. To ensure a fair comparison to the SNN pipelines, we break down computational costs by processing stage for each decoding strategy, and the reported totals reflect the full pipeline. For motor unit-based approaches, this includes motor unit decomposition. For the linear and RNN baselines, it includes spike-count binning and convolution operations (see Supplementary Methods 2). While the RNNs achieved comparable accuracy to the linear baseline and the SNNs, they did so at higher resource demands. In terms of memory footprint, the recurrent models required more storage than both the linear decoder and the SNNs, with the GRU exhibiting the largest footprint, followed by the vanilla RNN. This increase arises from the need to store recurrent weight matrices and hidden-state parameters (see Supplementary Methods 1). A similar ordering was observed when considering computational cost. The recurrent models incur substantially higher operation counts, measured as the average number of floating-point operations (FLOPs) per sample, compared to both the linear baseline and the SNNs.

In contrast, the SNNs required markedly fewer operations, quantified in synaptic operations (SynOp), due to their event-driven computation. In the decoder-level comparison reported below, we exclude the cost of motor unit decomposition, as it is a shared upstream stage in both pipelines. Under this condition, the quantified reduction corresponded to at least one order of magnitude fewer operations relative to recurrent models and remained substantial even when compared to the linear decoder. For example, for the extension direction of Subject 1—corresponding to the largest number of decomposed motor units and, consequently, the highest computational load—the GRU and vanilla RNN required 11.09 Mops and 22.48 Mops, respectively. By comparison, the SNNs variants required 63.60 and 64.60 kops when accounting only for effective operations elicited by incoming spikes. Under a conservative assumption of dense computation, corresponding to hardware that does not exploit sparsity (see Supplementary Methods 2), these costs increased to 2.73 Mops and 2.80 Mops, respectively, while the linear decoder required 1.39 Mops (see Tables S3 and S4).

Overall, under the present conditions, the non-linear recurrent architectures did not provide measurable gains in decoding performance, despite incurring higher memory and computational costs. For these reasons, we proceeded under the linearity assumption and adopted the linear baseline as the primary comparator to the SNNs in subsequent experiments.

**Fig. 4:**
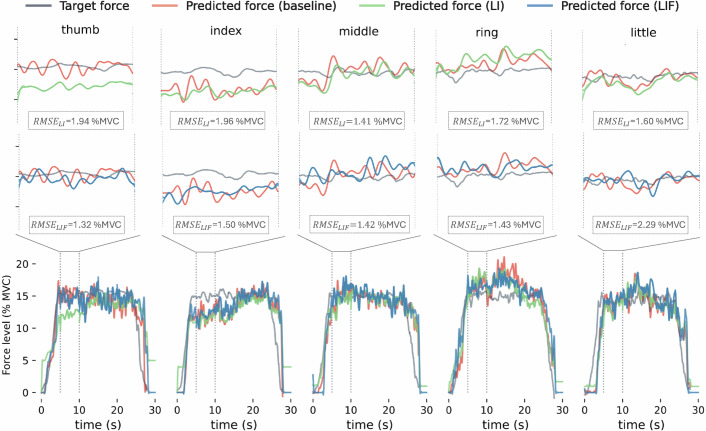
Example decoding performance of the baseline and SNN models during finger flexion tasks. Representative force estimation traces for Subject 1 (repetition 2) using motor unit spike trains as input. The five columns correspond to individual finger flexion tasks (thumb to little finger). Top insets: zoomed view of the 5–10 s interval, showing target force (black) alongside predicted forces from the baseline linear regression model (red), the LI configuration (green), and the LIF configuration (blue). RMSE (% MVC) for each model is reported in each inset. Bottom panels: full 30-s contraction profiles for each task, with traces for non-targeted fingers omitted for clarity.

Representative decoding outputs from single trials of the five-finger tasks are shown in Fig. 4 (see Figs. S2–S4 for additional examples). Each column corresponds to a different finger, and for clarity, only the trajectory of the actively engaged (i.e., highly activated) finger is depicted. Although all models decoded forces for all five fingers simultaneously, non-target finger outputs are omitted for visual clarity. Across all models, the overall shape of the force profile—defined by the rising, plateau and falling phases of the isometric contractions—was reliably captured. However, finer modulations in force during the plateau phase were not consistently reproduced, as highlighted in the zoomed five-second inset. This limitation likely stems from an incomplete sampling of the motor unit activity required to recover those fluctuations. As a consequence, subtle variations in motor unit discharge rates encoding fine force adjustments may have been partially lost^17^, thereby limiting the decoder’s ability to track slow force changes during sustained contractions. While this limitation may pose challenges for applications that demand high-fidelity force reconstruction—such as precision control in virtual reality scenarios—it is likely less critical in assistive contexts like prosthetic hand control for amputees, where capturing fine fluctuations is often unnecessary. Nevertheless, further analysis is needed to assess the functional implications of this limitation and to explore strategies for improving resolution during sustained-force decoding from motor units.

We also acknowledge that the limited sample size constrains the statistical power of these comparisons. Accordingly, decoding accuracy comparisons are interpreted descriptively using effect sizes and confidence intervals. The results suggest that shallow SNN architectures composed of LI units or LIF neurons can, in principle, achieve decoding performance on par with conventional regression pipelines using the decomposed motor unit spike trains. A key advantage of the approach presented lies in its event-driven, continuous processing capability, which eliminates the need for explicit memory buffers or spike count accumulation. Instead, the network leverages the intrinsic state dynamics of the neurons, which integrate and decay over time according to their membrane time constants.

### Networks resilience to real-time decomposition errors

Real-time decomposition of motor unit activity typically relies on precomputed separation matrices, derived offline, which are subsequently applied to streamed EMG signals for online motor unit identification^39,40^. This strategy circumvents the computational cost of performing blind source separation in real time, but introduces trade-offs: variations in motor behavior, electrode placement, or signal quality between the calibration phase and the online phase can influence both the number of motor units detected and the temporal precision of their identified discharge timings.

To simulate the effects of these limitations in a controlled setting, we introduced various perturbations to the input motor unit spike trains in the form of random spike omissions (false negative errors), spike additions (false positive errors), or cross-motor unit misattributions, which combine both decomposition error types^42^. Rather than explicitly retraining or recalibrating the decoders, we evaluated the impact of these perturbations at inference time to quantitatively assess the intrinsic noise resilience of the baseline model and SNN decoding architectures.

We hypothesized that the spiking intermediate layer configuration would confer greater robustness to decomposition errors compared to both the baseline model and the leaky direct-readout configuration, due to the thresholding properties of LIF neurons. By filtering out small membrane fluctuations induced by missing or added spikes, spiking neurons can inherently stabilize the network output against noisy inputs.

**Fig. 5:**
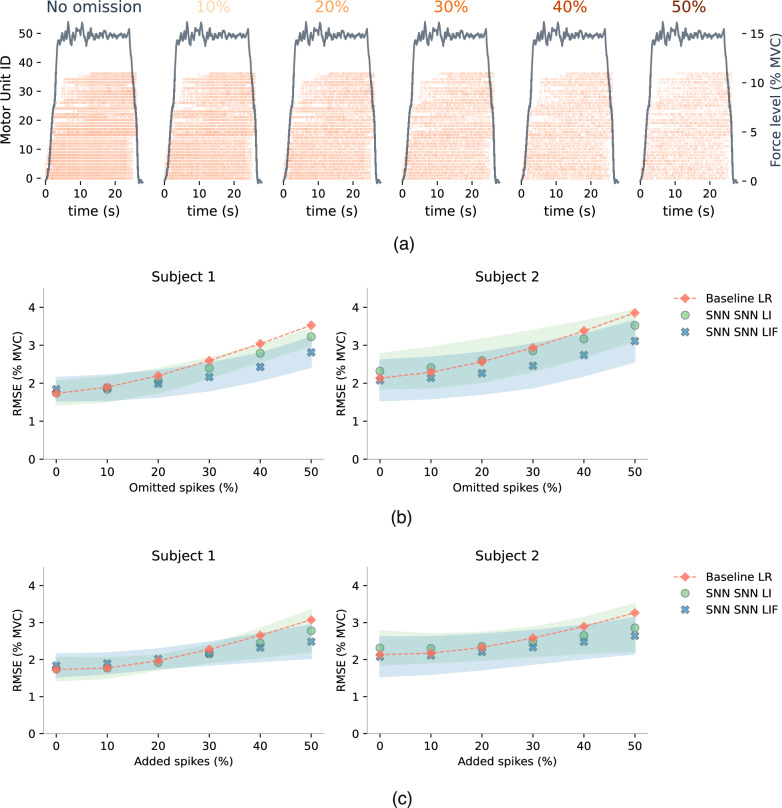
Robustness of SNN configurations to random spike omission and addition during inference. **a** Raster plots of decomposed motor unit spike trains from a representative trial (middle finger flexion, repetition 2, Subject 1) under increasing levels of motor unit spike omission (from 0% to 50%). Spike omissions were applied randomly during inference to simulate decomposition noise. Overlaid force trajectories (% MVC) correspond to the target force level during the task. Decoding performance is reported as RMSE across all tasks and movement directions for both subjects as a function of omitted spikes (**b**) and added spikes (**c**). Models were trained on clean motor unit spike trains (no omission or addition) and evaluated under increasing levels of perturbation. RMSEs are presented as the aggregate mean values across tasks and movement directions, and shaded areas indicate  ± SD. The standard deviation of the baseline model was omitted for clarity and is reported in Tables S6–S8.

Figure 5 illustrates the degradation in decoding performance, quantified by RMSE, under increasing levels of spike omissions (Fig. 5a) and additions (see Tables S6–S8 for corresponding MAE and *R*^2^ metrics). As expected, performance declined with higher omission rates across all models (Fig. 5b); however, the LIF configuration consistently exhibited greater robustness compared to both the baseline linear model and the LI configuration. In Subject 1, for example, the RMSE of the baseline model increased from 1.73 ± 0.26 to 3.53 ± 0.27 % MVC at 50% spike omission, while the LI model rose from 1.74 ± 0.32 to 3.22 ± 0.22 % MVC. The LIF model showed a more moderate increase from 1.84 ± 0.31 to 2.81 ± 0.40 % MVC, indicating improved resilience to spike loss. Examples of decoded force trajectories across omission levels are provided in Fig. S5.

In contrast, as shown in Fig. 5c, all decoders demonstrated less overall sensitivity to false positive errors, with differences between decoders becoming more apparent at high error levels (≥40%). For Subject 1, the baseline linear regression (LR) model increased from 1.73 ± 0.26 to 3.07 ± 0.58 % MVC at 50% spike addition, whereas the LI model increased from 1.74 ± 0.32 to 2.78 ± 0.57 % MVC and the LIF model from 1.84 ± 0.31 to 2.48 ± 0.45 % MVC. However, the limited sensitivity in this context should be interpreted with caution, as the results are partly shaped by the temporal structure of the force profiles used in this study. Random spike additions (Fig. S6) are more likely to affect transient ramp phases, where they primarily accelerate force onset rather than substantially perturb steady-state force levels (see Figs. S7 and S8). Because these ramp phases constitute a relatively small fraction of each trial, their influence on the overall RMSE is correspondingly limited. Consequently, spike-addition perturbations provide only partial insight into decoder robustness in this context. Further evaluation using alternative force trajectories (e.g., sinusoidal or triangular) is therefore required to more fully characterize the impact of false positive decomposition errors.

For cross-motor unit misattribution errors, performance differences across decoders were relatively small, including for the baseline LR model (see Fig. S9). In Subject 1, the LR RMSE increased from 1.73 ± 0.26 to 1.81 ± 0.25 % MVC at 20% misassigned spikes, while the SNN LI and LIF models increased from 1.74 ± 0.32 to 1.75 ± 0.33 % MVC and from 1.84 ± 0.31 to 1.89 ± 0.31 % MVC, respectively (see Table S9 for corresponding MAE and *R*^2^ metrics). This limited sensitivity likely reflects the fact that misattribution preserves the overall spike timing and event count, redistributing activity across motor units rather than removing or adding spikes; at moderate error levels (0–20%) and with many units contributing to force generation, such redistributions produce only modest changes in the decoded force trajectory (see Fig. S10).

The observed robustness to the false negative decomposition errors, however, comes at the cost of a marginal increase in both the parameter space and computational costs. As shown in Fig. 3 and detailed in Tables S2–S5, the memory required to store model parameters, including the decomposition separation matrix, is marginally higher for the LIF decoder compared to the LI configuration. For Subject 1, for instance, the parameter memory footprint increased from 98,028 bytes for the LI configuration to 98,156 bytes for LIF, with an increase of 1k SynOp, reflecting the addition of the intermediate spiking layer. Although the increase is negligible in absolute terms, it still reflects a design trade-off. Whether this trade-off matters ultimately depends on the deployment setting and the available resources of the intended application. It is important to note that these values represent a conservative, worst-case estimate, as they assume full 32-bit floating-point precision for all parameters. In practice, memory requirements can be substantially reduced through quantization, albeit at the potential cost of reduced performance. A systematic analysis of the trade-off between memory optimization and decoding accuracy, however, lies beyond the scope of this study and is left for future work.

### Encoding HD-iEMG into events bypassing motor unit decomposition

**Fig. 6:**
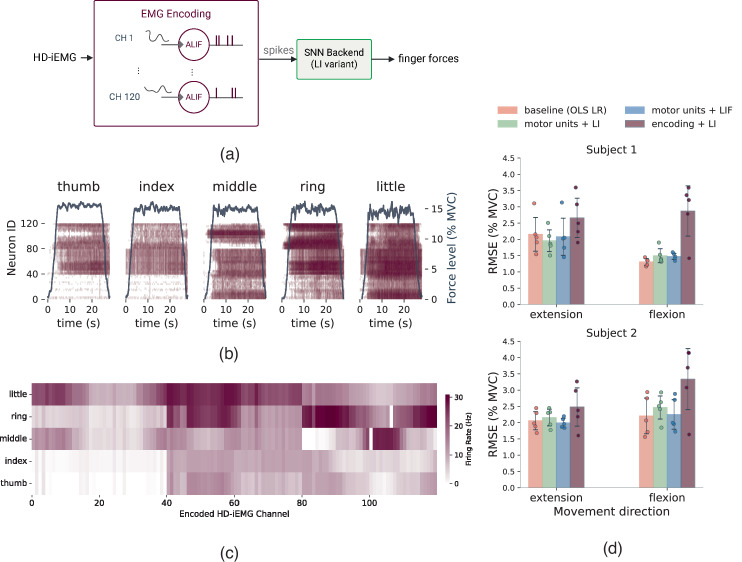
Decoder performance using spike-encoded HD-iEMG signals. **a** Schematic of the processing pipeline for decoding finger forces from HD-iEMG: filtered signals from 120 channels are spike-encoded using ALIF neurons and passed to an SNN backend. The baseline threshold *θ*_base_ and adaptation scale *w*_*θ*_ were set to 0.1 V, with an adaptation time constant of 100 ms. Created in BioRender. Baracat, F. (2026) https://BioRender.com/jiw6ygs. **b** Example raster plots of spikes generated by the encoding stage in response to the iEMG inputs (Subject 1, flexion tasks, repetition 2). Force trajectories (in % MVC) are overlaid for reference. **c** Corresponding example average firing rates of the encoding neurons across finger tasks. **d** Decoding performance reported as RMSE (% MVC) across finger tasks for each subject, separated by movement direction. Each dot represents the mean RMSE for a single finger, averaged across two repetitions (*n* = 5 fingers). Bars show the mean across fingers, and error bars indicate  ± SD.

In parallel with the use of decomposed motor unit spike trains, we investigated in a third experiment whether direct event-based encoding of HD-iEMG signals could support proportional and simultaneous decoding of finger forces (Fig. 6a). This approach avoids the computational overhead associated with offline motor unit decomposition and eliminates the need for storing separation matrices during real-time operation. However, it introduces an additional processing stage to convert the multichannel EMG signals into spike trains, which adds to the pipeline footprint and computational costs. At the same time, because this strategy essentially relies on global features extracted from EMG activity rather than direct neural discharge information, a reduction in decoding accuracy relative to motor unit-based approaches was anticipated. The objective of this experiment is therefore to quantify the extent of information loss incurred when bypassing decomposition, and to evaluate whether the resulting trade-off between decoding performance, computational cost, memory footprint, and implementation feasibility justifies favoring this strategy for real-time control scenarios despite the additional cost introduced by an encoding stage.

Filtered HD-iEMG signals were encoded into spike trains using an additional layer of ALIF neurons configured with homogeneous membrane time constants. Because the three microelectrode arrays were implanted at anatomically distinct sites, the amplitude distributions of the recorded intramuscular electromyography (iEMG) signals varied across tasks and subjects (see Fig. S11), effectively calling for a normalization mechanism. To avoid manual tuning of the encoding neurons’ thresholds for this purpose, adaptive neurons were therefore employed at this stage. Through threshold adaptation, neurons dynamically modulate their firing thresholds based on recent spiking activity, yielding a more balanced distribution of firing rates across channels, without the need for manual calibration of thresholds. An example of the spike-encoded representation during finger flexion tasks is shown in Fig. 6b with the corresponding event rates across the HD-iEMG channels reported in Fig. 6c.

After encoding, the resulting spike trains were directly streamed into the same leaky SNN architecture used for motor unit-based decoding, for a systematic comparison between the two input modalities under identical network configuration. Importantly, the number of trainable parameters remained unchanged; the encoding layer simply replaced physiological motor unit spike timing information with a binary threshold-based representation, without increasing model complexity, defined in terms of the number of trainable parameters.

Performance metrics for motor unit-based decoding were previously reported (see “Streamed processing of motor units spike trains with neurons and synapses”) and further compared across task directions in Fig. 6d. Decoding based on spike-encoded HD-iEMG signals resulted in higher RMSE values as expected, with the leaky configuration reaching 2.76 ± 1.57 % MVC for Subject 1 and 2.91 ± 1.15 % MVC for Subject 2 (see Table 1). This represents a performance drop of ~0.6–1 % MVC relative to motor unit-based SNN decoders (see Fig. S12 for an example decoded force trajectories using spike-encoded HD-iEMG).

**Fig. 7:**
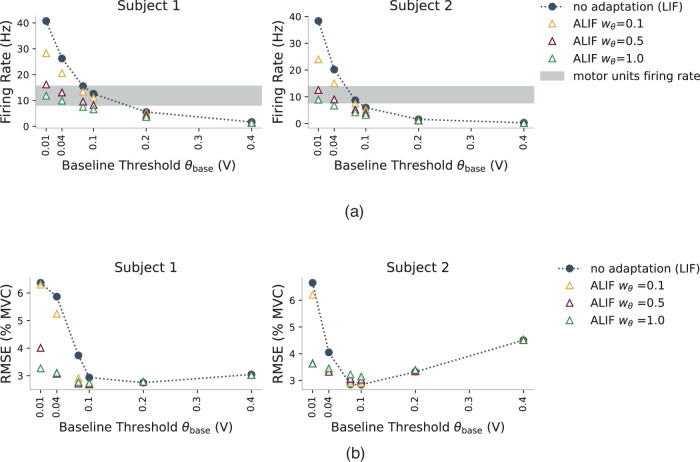
Sensitivity analysis of encoding neuron adaptation parameters. **a** Effect of varying the baseline spiking threshold, *θ*_base_, and the adaptation scale, *w*_*θ*_, on average firing rates across encoding neurons and finger tasks. Markers indicate the average firing rates across all input channels, cross-validation folds and movement directions. **b** Impact on decoding performance measured in terms of RMSE (% MVC). Markers indicate the average RMSE across cross-validation folds and movement directions. The no-adaptation condition corresponds to networks using LIF neurons with fixed firing thresholds in the encoding stage and is compared against adaptive ALIF models.

To further characterize the role of threshold adaptation in shaping the firing patterns of the encoding stage and their impact on downstream decoding performance, the influence of the adaptation parameters was investigated through a sensitivity analysis. As shown in Fig. 7 and further detailed in Figs. S13 and S14, increasing the baseline threshold *θ*_base_ progressively reduces the mean event rates, as expected. Notably, an intermediate operating regime emerges at *θ*_base_ = 0.1 V and *w*_*θ*_ = 0.1 V, in which the average event rate is ~10 Hz for both subjects. This regime matches the firing rates of the motor units for which the networks were tuned, explaining why both excessive spiking and overly sparse activation outside this operating range are associated with increased decoding errors.

Despite the drop in accuracy, spike-encoded HD-iEMG representations offer a compelling tradeoff in terms of both memory and computational efficiency. Unlike motor unit-based decoders, they do not require storage of decomposition separation matrices, resulting in memory and computational costs that are independent of the number of extracted motor units. In contrast, motor unit-based pipelines incur a subject-dependent overhead associated with the decomposition stage, which dominates both memory footprint and inference-time computation.

It is important to highlight that whether an increase of ~0.6–1% MVC in RMSE is acceptable depends on the target application and its tolerance to error. For tasks requiring very fine force modulation, even small deviations may be functionally relevant. However, in many assistive control scenarios, decoding errors on the order of a few per cent of MVC are commonly reported and may remain within a practically usable range, particularly when weighed against substantial reductions in memory and computational requirements.

For example, in Subject 1, the extension decoder required an additional 94,080 bytes, assuming full-resolution (32-bit float) representation, solely to store the separation matrix corresponding to 196 identified motor units, as shown in Fig. 3 and detailed in Table S2. The memory advantage of bypassing the decomposition is not, however, uniform across subjects: in Subject 2, where fewer motor units were identified (51 for flexion and 93 for extension), the memory required to store the separation matrices does not exceed the footprint of the spike-encoding stage. This comparison should be interpreted in light of how memory is accounted for, as the encoding footprint includes the full synaptic matrix for that stage, including zero-valued entries (see Supplementary Methods 1). Consequently, when the number of decomposed motor units is lower than the original HD-iEMG input dimensionality, spike-based encoding offers a clear memory footprint advantage.

From a computational standpoint, motor-unit decomposition constitutes the dominant contribution to the overall computational cost for both subjects. By operating directly on the raw EMG signals and bypassing this stage, spike-based encoding introduces only a minor additional overhead, resulting in inference-time computational costs that are up to two orders of magnitude lower (~10^3^ to 10^5^ FLOPs/SynOp) than those of motor unit-based pipelines.

Together, these observations highlight a fundamental tradeoff: while spike-encoded HD-iEMG inputs result in slightly reduced accuracy, they achieve that with lower memory demands and computational cost. Ultimately, the choice between physiologically plausible motor unit inputs and efficient spike-based encoding should be guided by the specific accuracy, memory requirements, and computational resources available for the intended application.

### Decoding finger forces from motor units on neuromorphic hardware

All results presented thus far were obtained in software simulation, where the proposed decoding framework was evaluated independently of any specific hardware implementation. This decoupling allowed us to establish baseline performance under idealized conditions. We now investigate how the framework translates to physical neuromorphic hardware, and how hardware-specific constraints affect decoding accuracy, latency, and power consumption.

In our hardware experiments, a shallow SNN with a spiking output layer, analogous to the LIF-SNN configuration presented earlier, was mapped onto the (DYNAP-SE) mixed-signal neuromorphic processor^43^. Because the DYNAP-SE does not support on-chip learning, network parameters were optimized using a chip-in-the-loop training framework to map decomposed motor unit activity to individual finger forces as a proof of concept (see “Spiking neural network on asynchronous neuromorphic hardware”). Given the chip fan-in limit of 64 incoming connections per neuron, the hardware demonstration was restricted to data from Subject 2.

**Fig. 8:**
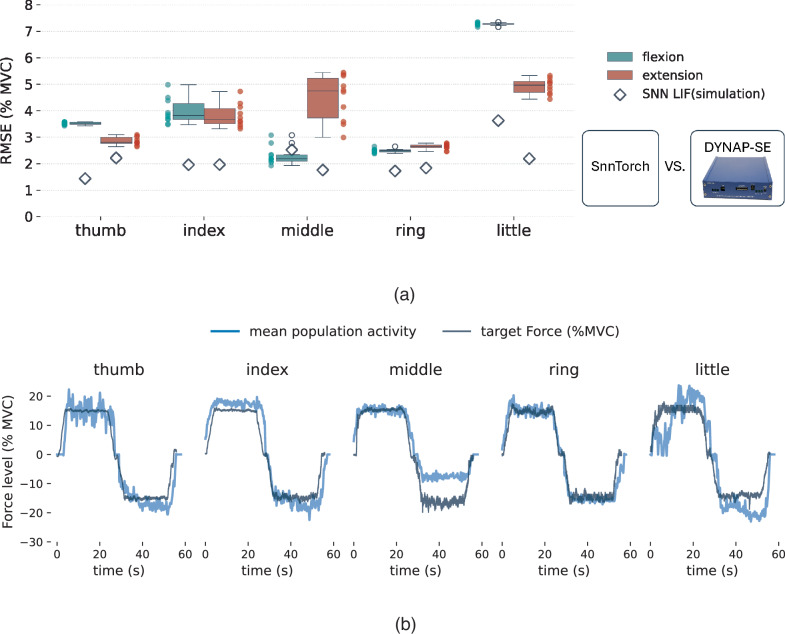
Decoding finger forces on DYNAP-SE. **a** Average test RMSE (in % MVC) for each finger, reported separately for flexion and extension. Box plots summarize performance across 10 independent runs (*n* = 10) collected on multiple days, with individual runs overlaid as scatter points. Box plots show the median (centre line), interquartile range (box; 25th–75th percentiles), and whiskers extending to the minimum and maximum values. Diamond markers indicate the corresponding RMSE obtained from the simulated SNN LIF configuration. **b** Example predicted forces on-chip (repetition 2). Two complementary neuron populations (five neurons each) were allocated on the chip to decode flexion and extension forces. Predictions were obtained from population activity smoothed using an exponential kernel (time constant 200 ms). Extension forces are displayed as negative values for visual clarity.

Figure 8 a summarizes the on-chip decoding performance across multiple repeated runs and finger tasks. Overall, on-chip decoding achieved an RMSE of 3.82 ± 1.62 % MVC, compared with 2.12 ± 0.41 % MVC obtained for the LIF configuration when training and testing were performed entirely in simulation using full-precision weights. Representative predicted force trajectories are shown in Fig. 8b, illustrating that the decoded signals generally follow the target force profiles. Nonetheless, deviations can be observed in specific cases; for example, during the middle finger task, the extension-phase plateau level is not fully captured.

This reduction in performance should be interpreted in light of the fundamental differences between simulation and hardware implementation of SNNs. In particular, on chip, synaptic weights can only assume discrete strength levels, which are implemented by adding or removing excitatory and inhibitory synapses, rather than by adjusting continuous real-valued parameters. In addition, network training was limited to modifying synaptic connectivity, while neuronal and synaptic time constants were fixed. These constraints, therefore, reduced the effective parameter space available during optimization and contributed to the observed loss in accuracy.

Even more broadly, these limitations reflect the use of a general-purpose neuromorphic platform. The DYNAP-SE processor was not specifically optimized for regression-based force decoding from motor unit activity, and the reported performance therefore only mirrors the constraints of a flexible research platform rather than those of a task-specific design. Neuromorphic processors incorporating on-chip synaptic plasticity and adaptive time constants are likely to enable further improvements in decoding accuracy^44^. The present results thus provide a realistic baseline for performance under current hardware constraints.

At the same time, the proposed decoding pipeline on-chip retains key advantages of neuromorphic processing. Motor unit spike trains are processed online as they arrive, without the need for external memory or buffering, in a manner analogous to biological neural circuits. Computation is performed in real time, enabling continuous inference. Across all runs, repetitions, and fingers, the average inference latency was 35.96 ± 99.81 ms. Importantly, this real-time computation is achieved at a total estimated power of only 13.24 μW (see Table S10 for a detailed breakdown).

Together, these results demonstrate that regression-based decoding of finger forces from motor unit activity can be implemented on neuromorphic hardware with acceptable accuracy, low latency, and ultra-low power consumption. Although performance is reduced relative to software simulation, the proposed framework remains effective under realistic hardware constraints.

## Discussion

Proportional and simultaneous decoding of individual finger forces from EMG signals is a key milestone towards naturalistic motor control for assistive technologies. Such systems must achieve accurate decoding with low latency and high energy efficiency. In this study, we integrated state-of-the-art HD-iEMG acquisition, motor unit decomposition, and SNNs to simultaneously decode individual finger force trajectories and systematically evaluate architectural trade-offs in accuracy, robustness, computational cost, and memory footprint. Although experiments were performed using HD-iEMG to ensure high-quality decomposition, the overall framework is, in principle, applicable to high-density surface electromyography (HD-sEMG). Compared with HD-iEMG, HD-sEMG is expected to yield fewer identifiable motor units and noisier decomposition due to increased cross-talk, potentially reducing absolute decoding performance without altering the architectural trade-offs or relative benefits of spike-based processing.

Neuromorphic computing has recently gained traction for efficient neural decoding in implantable brain-machine interface (iBMI) systems^45–47^. Achieving finer-grained decoding of user intent requires denser electrode arrays, substantially increasing data bandwidth and complicating wireless transmission under strict implant power and safety constraints^9^. Neuromorphic systems mitigate these limitations through early-stage event-driven computation near the sensor, encoding analog activity into sparse spike trains that reduce communication bandwidth^48^. By co-localizing memory and computation, they also avoid the latency and energy overheads associated with buffering chunks of data between processing stages. Similar constraints are emerging in motor unit-based EMG decoding, where high-density recordings are increasingly adopted to resolve larger motor unit pools^49,50^. As signal dimensionality grows, efficient low-latency edge processing becomes increasingly important, making neuromorphic approaches well-suited to this application.

We demonstrated that shallow SNNs with fixed synaptic time constants achieved decoding performance on par with the linear regression approach traditionally employed for motor unit-based force decoding^23^, using only synaptic weights and membrane time constants as trainable parameters (Fig. 2). To examine the implicit linearity assumption, we compared the SNNs against lightweight recurrent architectures. Under matched parameter budgets, compact recurrent baselines (vanilla RNN, GRU) showed no measurable accuracy gain over the SNNs or the linear decoder (Table 1), yet incurred substantially higher computational costs (Fig. 3). Although these results cannot rule out nonlinear temporal structure in the data, particularly given the limited dataset size, they suggest that such structure is not effectively exploited by compact recurrent models under the present conditions.

We further investigated how architecture affects decoding robustness. Incorporating a spiking intermediate layer improved resilience to decomposition errors at inference time (Fig. 5) relative to the direct-readout architecture. This effect likely arises from the thresholding dynamics of spiking neurons, which attenuate minor input perturbations. The improvement, however, incurs an additional memory footprint of 128 bytes and an additional computational cost of 1k SynOp (Fig. 3). The robustness analysis in this study was limited to decomposition-level errors, including false positives, false negatives, and cross-motor-unit misattributions during online decomposition. These perturbations approximate mild instabilities caused by electrode shifts, impedance changes, or motion artifacts within the same recording session. More substantial decomposition errors may arise across sessions or days due to stronger recording non-stationarities. Assessing decoding robustness under such longitudinal conditions is left for future work, as it would require dedicated experimental protocols.

Motor unit discharge activity provides a direct and physiologically grounded representation of the spinal output driving muscle contraction, and thus supports highly accurate proportional decoding. However, obtaining this information through decomposition remains computationally demanding. Consequently, decomposition is typically performed offline, with precomputed separation matrices later used for online decoding^39^. Although this avoids real-time source separation, storing and projecting signals through these matrices still incurs memory overhead. To address these limitations, we explored an alternative approach based on directly encoding filtered HD-iEMG signals into event-based spike trains using a layer of ALIF neurons.

We showed that a neuron model with adaptive firing threshold reduces the need for manual tuning across subjects, despite variability in electrode placement, signal amplitudes, and channel activation patterns. Moreover, we demonstrated that the event rate produced by the encoding stage critically influences downstream decoding performance, revealing an operating range in which event rates are neither excessively sparse nor overly dense (Fig. 7). Notably, this analysis was conducted without re-tuning the downstream decoder. Instead, we focused on substituting the decomposition stage with spike-based encoding to enable a fair comparison between the two approaches.

This method bypasses decomposition entirely, offering a lower-complexity alternative more suitable for real-time applications at the edge, albeit with an expected trade-off in decoding precision. We showed that shallow SNNs could still reconstruct force trajectories from these encoded events with reasonable accuracy (Table 1). Decoding performance degraded compared to motor unit-based input, reflecting the expected information loss when moving from neural to global signal features^22^. Nevertheless, the observed performance gap remained modest (0.6–1% MVC) and was accompanied by a pronounced reduction in inference-time computational cost of approximately two orders of magnitude. These considerations suggest that event-based EMG encoding could in practice be a viable compromise when decomposition is impractical or infeasible, particularly if combined with adaptive thresholding mechanisms, or alternatively with heterogeneous neural populations to improve sensitivity to input amplitude variability^51^.

Our results support a neuromorphic hardware architecture in which a first spiking layer operates near the acquisition unit to perform early-stage processing, either as an input encoder or as a LIF layer receiving decomposed motor unit events. A second continuous decoding layer could then be integrated into the end effector to generate real-time force commands (Fig. 1d). Communication between modules would rely on sparse spike trains, reducing transmission bandwidth^45^. This motivates a fully embedded multi-core neuromorphic solution for prosthesis control. Although neuromorphic platforms already exist^52,53^, they do not yet integrate all components identified in this study.

To evaluate the feasibility of this architecture, we therefore implemented a proof-of-concept realization on the general-purpose neuromorphic processor DYNAP-SE. Decoding accuracy was benchmarked against full-precision software simulation results, while latency and power consumption were measured directly on chip. For Subject 2, with a limited synaptic weight precision, decoding RMSE increased from 2.12 ± 0.41% MVC to 3.82 ± 1.62% MVC. Nevertheless, the system achieved real-time operation with an average inference latency of 35.96 ± 99.81 ms and an estimated power consumption of 13.24 μW. These results provide a conservative estimate of achievable performance on a general-purpose analog neuromorphic substrate. It is also important to emphasize that this hardware demonstration was performed in open loop after electrode explantation. Closed-loop evaluation with continuous user interaction and end-to-end latency measurements will therefore be necessary to assess practical usability and clinical translation.

Our results provide specifications that can inform the development of a complete stand-alone hardware implementation. However, a few aspects remain to be addressed in future work. First, conclusions are based on only two male participants due to the practical constraints of intramuscular EMG recordings. The study, therefore, emphasizes within-subject trends and architectural trade-offs rather than population-level generalization, which will require larger and more diverse cohorts. Second, the evaluation was limited to moderate-force isometric contractions. More dynamic tasks, such as time-varying force trajectories, may require richer temporal modeling and higher-capacity recurrent architectures. Finally, a separate decoder was trained for each participant, which is not scalable in practice. Future work could address this through hybrid approaches combining offline pretraining on pooled datasets with lightweight on-chip adaptation or few-shot calibration.

## Methods

### Ethics

The data analyzed in this study were collected as part of a previously approved experimental protocol involving human participants^38^. All procedures were conducted in accordance with the Declaration of Helsinki and approved by the relevant institutional review boards at Imperial College London (ICREC Project ID 19IC5640 for intramuscular recordings; JRCO Project ID 18IC4685 for MRI acquisition). All participants provided informed written consent, including consent for the use of their data in subsequent analyses. No personally identifiable information is included in this manuscript.

### High-density intramuscular EMG dataset

HD-iEMG signals were recorded from two healthy male participants using three multi-channel intramuscular MEAs^36^ inserted into the forearm muscles. Electrode placement targeted the extrinsic muscles responsible for finger flexion and extension, and was guided by anatomical landmarks and real-time ultrasound imaging to ensure accurate and consistent positioning across both individuals. Each array consisted of 40 electrodes (140 μ m × 40 μm), resulting in 120 recording sites in total. EMG signals were acquired using a multichannel amplifier system (OT-Bioelettronica, Torino, Italy), sampled at 10240 Hz, and digitized at 16-bit resolution.

In parallel with the HD-iEMG recordings, finger forces were measured using ten load cells (TAL220, 10 kg resolution), with two sensors assigned to each finger to independently capture flexion and extension force components. Further details regarding the electrode configuration and experimental setup are available in ref. ^38^.

Participants completed a series of isometric finger activation tasks, following a predefined trapezoidal force profile set at 15% of their maximum voluntary contraction (MVC). During each task, subjects were instructed to activate only one finger while keeping the others at rest. Although each task was designed such that only a single finger was intentionally activated at 15% MVC, the remaining fingers occasionally produced unintentional forces (see Fig. 1c). Importantly, forces from all five fingers were recorded continuously in every trial, ensuring that, even when only one finger was voluntarily engaged, the dataset captured the complete force profile across all digits. Each force trajectory lasted 26 s, consisting of a 3-second ramp-up, a 20-second isometric hold, and a 3-second ramp-down. Tasks were performed sequentially, one finger and one direction (flexion or extension) at a time, and each task was repeated twice. All analyses presented in this study are based on data collected within a single experimental session per subject.

### Motor unit decomposition

HD-iEMG signals were decomposed into individual motor unit spike trains using a blind source separation algorithm designed for high-yield extraction^37^. Decomposition was carried out independently for the isometric plateau phase of each finger task. For each task, signals from all EMG electrodes and both repetitions were concatenated and jointly decomposed to extract task-specific motor unit activity.

Extracted motor units were post-processed to ensure their quality and physiological plausibility. Only motor units with a silhouette value exceeding 0.85 and an average discharge rate within the expected range for sustained isometric contractions (4 Hz to 50 Hz) were retained. The silhouette score is a signal-based accuracy metric that measures the degree of separation between identified motor units and noise. Formally, it provides a normalized measure of clustering reliability, computed as the difference between within-cluster and between-cluster distances, applied to the separation between motor unit signals and noise^54^. Higher silhouette values indicate better separability and more reliable motor unit identification. To enhance temporal consistency and mitigate inter-repetition variability—particularly given the limited sample size—only motor units that were active in both repetitions of a given task were included in subsequent analyses. A detailed breakdown of the number of identified motor units for each subject is presented in Table S11.

### Baseline regression models

To contextualize the performance of the proposed SNN pipelines, two categories of baseline models were implemented: a simple OLS-LR and two lightweight RNNs: vanilla RNN and a GRU. The OLS-LR pipeline follows the traditional motor unit-based decoding used in literature^22,24^, while the RNN architectures set a non-linear baseline with temporal processing capabilities on par with SNNs.

#### Ordinary least-squares linear regression

The OLS LR operates on binned spike counts. For each movement direction (i.e., flexion, extension), decomposed motor unit spike trains from all corresponding finger tasks were pooled and segmented into 80 ms windows with 50% overlap. The window length was selected based on a sweep analysis in which the baseline model was evaluated across multiple window sizes ranging from 10 ms to 200 ms (see Fig. S1). A window size of 80 ms provided the best trade-off between decoding performance and responsiveness and was therefore fixed for all subsequent analyses. The degree of overlap between windows was not included in the sweep and was fixed to 50%, in line with our previous work^38^. Spike counts were computed within each window, min-max normalized, and used as input features to train a multi-output linear regression model. The predicted finger force trajectories were subsequently low-pass filtered with a cutoff frequency of 2 Hz to obtain smooth force estimates. The choice of a multi-output formulation is motivated by the structure of the recorded force signals: each trial contains the force trajectory of the intentionally activated finger as well as residual or unintentional forces from the other digits. Modeling all force channels jointly provides a more faithful mapping between the decomposed motor unit activity and the complete force profile, while also enabling straightforward scalability to future scenarios involving simultaneous multi-finger activations.

Separate models were trained for each subject and movement direction to decode the continuous force trajectories of all five fingers from the binned motor unit discharge activity. This design choice was motivated by the fact that motor unit decomposition was performed separately for each direction, resulting in non-overlapping sets of identified units across flexion and extension. Enforcing motor unit tracking across both flexion and extension would have substantially reduced the number of usable units, thereby limiting the richness of the input signal. Instead, by training independent models for each direction, the decoder could leverage a larger and more representative motor unit population for each task. From an architectural standpoint, this corresponds to two non-overlapping weight sets that can be combined into a unified decoder, where weights for the opposite direction are set to zero. This formulation preserves modularity while enabling future extensions that integrate direction-specific decoding into a single, comprehensive model.

#### Recurrent neural networks

The recurrent baselines operate on smoothened motor unit spike trains, obtained by convolving each spike train with an exponential kernel (decay time constant 10 ms), mirroring the synaptic filtering effect of the SNN decoders (see “Spiking neural network models in simulation”). Both models were configured with a number of trainable parameters closely matched to those of the SNN decoders to enable a fair comparison. The vanilla RNN consisted of a single recurrent layer with a hidden size of five units and no bias terms, with predictions read directly from the five hidden states. The GRU variant used the same hidden state dimensionality but incorporated an additional compact fully connected layer to map the recurrent state to the five finger force outputs.

### Spiking neural network models in simulation

For each subject and movement direction, a single-layer SNN was trained to estimate the continuous force trajectories of all five fingers. The network comprised *m* input neurons, where *m* corresponds to the total number of decomposed motor units pooled from all finger tasks associated with the given direction. Each input neuron is projected to five output neurons, with each output dedicated to predicting the force produced by a specific finger.

To avoid over-parameterization given the small dataset size, we adopted a single trainable layer in all SNN configurations. Deeper architectures were not explored, as they would substantially increase parameter count and memory footprint without a clear system-level motivation within the scope of this study.

#### Readout configurations

We evaluated two configurations for the trainable readout layer: a leaky configuration (LI configuration) and a spiking configuration (LIF configuration) (Fig. 1d). In the leaky configuration, output neurons were modeled as leaky integrators (LIs) that continuously accumulate synaptic input and directly produce smooth membrane potentials, which were later filtered to generate the final force predictions. In the spiking configuration, the output layer consisted of leaky integrate-and-fire (LIF) neurons, which emit spikes when their membrane potentials exceed a threshold. The resulting spike trains were then passed to an intermediate layer of LI neurons connected via instantaneous synapses with fixed weight, converting the discrete events into continuous-valued signals suitable for force decoding.

In both configurations, input motor unit spike trains were transmitted to the output neurons through synapses modeled as exponential kernels. Upon the arrival of a spike, a synaptic current *I*_syn_ is generated and scaled by the corresponding synaptic weight. These currents decay exponentially over time with a fixed synaptic time constant *τ*_syn_ and are integrated into the membrane potential *U* of each output neuron. Synaptic weights are optimized during training, while synaptic time constants remain fixed. The synaptic and membrane dynamics are described by the following equations:

1 $${I}_{{{\rm{syn}}},j}[t+1]=\alpha {I}_{{{\rm{syn}}},j}[t]+{\sum }_{i}{{{\bf{W}}}}_{ji}\,{{{\bf{x}}}}_{i}[t+1]$$

2 $${U}_{j}[t+1]=\beta {U}_{j}[t]+{I}_{{{\rm{syn}}},j}[t+1]$$

Here, **W**_*ji*_ is the synaptic weight from input unit MU_*i*_ to output neuron *j*, and **x**_*i*_[*t*] denotes the spike train of MU_*i*_ at time *t*. *I*_syn,*j*_ represents the cumulative synaptic input to neuron *j*, which is integrated into its membrane potential *U*_*j*_. The decay factors *α* and *β* are derived from the synaptic and membrane time constants, respectively, and are defined as $\alpha=\exp (-\Delta t/{\tau }_{{{\rm{syn}}}})$ and $\beta=\exp (-\Delta t/{\tau }_{m})$, with a discrete simulation time step Δ*t*.

In the LIF configuration, motor unit spike trains were integrated by a first layer of LIF neurons with a firing threshold of 0.045 V. When the membrane potential exceeded this threshold, a spike was emitted and the potential reset. The membrane time constants of these neurons were initialized from a normal distribution with mean *τ*_*m*_ = 30 ms and standard deviation 1.5 ms (5% of the mean). This value was heuristically chosen to be approximately two to three times larger than the fast synaptic time constant, ensuring sufficient temporal integration of synaptic inputs. The membrane time constants were treated as learnable parameters and jointly optimized with the synaptic weights during training.

Because the spiking output of this layer is inherently discrete, the resulting spike trains were transformed into continuous-valued signals by an intermediate LI layer with a fixed time constant *τ*_*m*,conv_ = 50 ms. This conversion stage extends the temporal integration window beyond the intrinsic dynamics of the spiking readout layer and was therefore chosen with a longer membrane time constant.

To further suppress residual high-frequency components of the membrane potential and ensure smooth force predictions, a second LI layer with a longer time constant of 80 ms was introduced, resulting in a double-stage filtering process applied only in the LIF configuration (Fig. 2a). This time constant corresponds approximately to a first-order low-pass filter with a cutoff frequency of ~2 Hz.

For the purely leaky (LI) configuration, the membrane time constant of the output neurons was fixed to *τ*_*m*_ = 80 ms, directly producing smooth membrane potentials without intermediate spike conversion. In both configurations, the final continuous force estimates were obtained by applying the same low-pass filtering procedure used in the baseline model (Methods 4.3).

All SNN models were implemented using *SnnTorch*^55^, and simulations were performed with a discrete time step of Δ*t* = 10 ms. A summary of all hyperparameters is provided in Table 2.

**Table 2:** Summary of the SNNs hyperparameters

Parameter	Description	Value
Δ*t*	Simulation time step	10 ms
*τ*_syn_	Fixed synaptic time constant (exponential kernel)	10 ms
weights	Synaptic weight initialization range	LI configuration: *U*( − 0.1, 0.1)
		LIF configuration: *U*( − 0.01, 0.01)
*τ*_*m*_	Membrane time constant of first layer of neurons (fixed or trainable)	LI configuration: 80 ms
		LIF configuration: ${{\mathcal{N}}}(30\,{{\rm{ms}}},1.5\,{{\rm{ms}}})$
*τ*_*m*,conv_	Time constant for spike-to-continuous conversion layer	LIF configuration: 50 ms
threshold	Firing threshold for LIF output neurons	LIF configuration 0.045 V
Train batch size	Number of samples per training batch	16
Test batch size	Number of samples per evaluation batch	1
Epochs	Number of training epochs	100
Learning rate	Optimizer learning rate	LI configuration: 0.01
		LIF configuration: 0.001
Surrogate gradient	Function used to approximate the derivative of the non-differentiable spike function during the backward pass	Fast sigmoid (slope = 25)

#### Training and inference

All SNN models were trained using surrogate gradient descent with a mean squared error loss and Adam optimizer. During training, the input repetition was segmented into overlapping 10-second windows with a 50% overlap. This window length was selected to balance computational efficiency and temporal context, ensuring that each training update was informed by a sufficient duration of motor unit activity and force output.

Once training was complete, the network was evaluated in inference mode under conditions that approximate real-time deployment, such as on always-on neuromorphic hardware. During inference, the batch size was set to one, and the model processed 10-second spike train segments sequentially. At each simulation time step (Δ*t* = 10 ms), it produced a continuous five-dimensional force estimate. To simulate uninterrupted, stateful processing within the PyTorch framework, neuron and synapse states were preserved across the segments without resets between inference windows. This workaround was applied solely to ensure a fair comparison with the baseline model. In mixed analog neuromorphic hardware, such state management is inherently handled, requiring no explicit intervention or additional memory buffers.

#### Offline and deployment-compatible stages

The proposed decoding framework comprises both offline and online components. Offline stages include signal preprocessing, calibration of motor unit decomposition (i.e., estimation of separation matrices), selection of encoding parameters, and model training. These procedures are performed prior to deployment and do not contribute to the real-time computational load during operation.

Online stages consist of spike encoding (when applicable), event-driven SNN inference, and force readout. In motor unit-based configurations, real-time execution assumes the use of precomputed separation matrices for motor unit identification, as is standard practice in practical systems. In the spike-encoded configuration, decomposition is bypassed, and encoding and decoding are performed sequentially on streamed input data.

All latency and power measurements reported for neuromorphic hardware (“Decoding finger forces from motor units on neuromorphic hardware”) correspond exclusively to the online inference stage. These measurements, therefore, reflect the real-time computational cost of decoding and exclude offline calibration and training procedures.

### Evaluation metrics and data splits

Model performance was assessed using standard regression metrics: RMSE, MAE, and the *R*^2^, computed under a twofold cross-validation scheme. Among these, RMSE was selected as the primary evaluation metric in the main text due to its interpretability: it quantifies the deviation from the target force as a percentage of MVC, providing a concrete measure of decoding accuracy.

Cross-validation was performed by splitting the data at the repetition level. All preprocessing steps, including spike binning for the OLS LR or convolution for the RNNs, were applied independently within each fold to prevent information leakage across splits.

To mitigate the risk of overfitting to fixed activation sequences, the order of finger activations was shuffled within each repetition prior to training. This randomized ordering was applied consistently across all models to ensure a fair comparison. In addition, training was repeated using ten different random initialization seeds for the SNN and RNN models, and reported performance metrics are averaged across seeds.

### Networks robustness analysis

To evaluate the robustness of the proposed SNN topologies to errors arising from online motor unit decomposition, controlled perturbations were introduced to the motor unit spike trains at inference time. All networks were trained exclusively on clean, offline-decomposed spike trains, and were subsequently evaluated under distinct modes of decomposition error, including missed detections (false negatives), false-positive detections^42^, and cross-motor unit misattributions.

False-negative errors were simulated by randomly removing a specified proportion of discharge events from motor unit spike trains at inference time, thereby modeling missed detections in online decomposition. False-positive errors were simulated by inserting additional spikes at randomly selected time points within the spike trains. Cross-motor unit misattribution was simulated by reassigning detected discharge events from their original motor unit to randomly selected neighboring motor units. For all perturbation modes, error levels were systematically varied to quantify their impact on decoding performance and to evaluate the stability of the trained models under increasing decomposition noise.

### EMG-to-spike encoding

A layer of ALIF neurons was used to convert the continuous iEMG signals into an event-based representation compatible with SNN processing. Each neuron was connected one-to-one to a corresponding filtered iEMG channel, thereby transforming continuous-valued signal amplitudes into characteristic spike patterns unfolded in time.

Because the three microelectrode arrays were implanted at anatomically distinct sites, the amplitude distributions of the recorded iEMG signals varied across electrodes and tasks (see Fig. S11). As a result, using a fixed neurons’ firing threshold for this encoding stage would lead to heterogeneous event rates across channels, with some producing dense spike trains and others remaining largely inactive. As the first stage of the network, such an imbalance would dilute the information conveyed by sparsely active channels while introducing redundancy in others, ultimately degrading the quality of the downstream representation. To account for inter-channel differences and to avoid manual tuning of encoding thresholds, adaptive neurons were therefore used at this stage.

#### Adaptive leaky integrate-and-fire neurons

Unlike LIF neurons, which maintain a constant firing threshold, ALIF neurons modulate their threshold based on recent spiking activity to discourage sustained firing and promote temporal sparsity. The effective threshold is implemented as the sum of a baseline threshold *θ*_base_ and an adaptive component *θ*_adapt_[*t*]:

3 $$\theta [t]={\theta }_{{{\rm{base}}}}+{\theta }_{{{\rm{adapt}}}}[t].$$

The adaptive component is updated in discrete time based on the spiking activity *S*[*t*] ∈ {0, 1} as

4 $${\theta }_{{{\rm{adapt}}}}[t+1]={\beta }_{{{\rm{adapt}}}}\,{\theta }_{{{\rm{adapt}}}}[t]+(1-{\beta }_{{{\rm{adapt}}}})\,S[t]\,{w}_{\theta },$$

where *w*_*θ*_ is the scale parameter controlling the amplitude of threshold adaptation, and the decay factor is given by ${\beta }_{{{\rm{adapt}}}}=\exp (-\Delta t/{\tau }_{{{\rm{adapt}}}})$. In the absence of spikes (*S*[*t*] = 0), *θ*_adapt_ decays exponentially toward zero with time constant *τ*_adapt_, such that *θ*[*t*] relaxes back to *θ*_base_. For both subjects, the baseline threshold *θ*_base_ and *w*_*θ*_ were set to 0.1 V and *τ*_adapt_ to 100 ms.

### Decoders footprint

The memory footprint of each decoder was estimated based on the number of parameters and their storage precision. All parameters in the baseline linear regression models and SNN decoders were stored at full precision (four bytes). Full weight matrices were included in the footprint calculation for consistency across decoder architectures, including zero-valued entries arising from one-to-one connectivity patterns. This choice reflects the static memory requirements associated with storing complete parameter tensors in standard implementations. To account for the presence of inactive or pruned connections, we additionally report the connection sparsity as a complementary metric, which quantifies the proportion of zero-valued weights (see Table S2). A sparsity value of zero indicates a fully connected network, whereas a value of one corresponds to the absence of connectivity. The resulting static metrics, i.e., footprint and connection sparsity, follow the formulation proposed in ref. ^56^. A complete description of the decoder footprint is provided in Supplementary Methods 1.

For all decoders operating on motor unit spike trains, we further included the memory required to store the separation matrix used during decomposition. This matrix, composed of 32-bit floats, has dimensions equal to the number of identified motor units multiplied by the number of EMG channels.

### Decoders computational and resource costs

We evaluated the computational and resource costs of each decoder and its associated processing pipeline by measuring the total number of operations, average wall-clock inference time, and average and peak runtime memory requirements required to process a single input sample during inference (forward pass only). Operation counts are reported as the average number of operations per input sample. A detailed description of the operation counting procedure is provided in Supplementary Methods 2.

For the baseline RNN decoders, the total computational cost was obtained by summing the contributions of the pre-processing convolutional stage and the recurrent decoding stage. The convolutional layer is used to transform motor unit spike trains into smoothed discharge patterns prior to decoding and was therefore included in the cost breakdown.

For the SNN decoders, computational costs were quantified in terms of SynOp. To capture the impact of activity sparsity, SynOp were further decomposed into dense and effective components. Dense SynOp provide an upper bound on computational cost and were computed under a fully time-clocked assumption, in which all synapses are evaluated at every time step, with no reliance on event-driven execution or hardware support for sparse spike representations. Effective SynOp account only for synaptic events triggered by non-zero inputs and spiking activity, thereby reflecting the actual computational workload incurred during inference on hardware architectures that support sparsity. For an encoding layer operating on continuous-valued signals, SynOp are implemented as multiply-accumulate (MAC) operations.

### Statistical analysis

As this study includes only two subjects and repeated measurements within each subject, we report effect sizes together with 95% confidence intervals to describe the magnitude of performance differences between decoding pipelines, rather than relying on inferential statistical tests.

For each subject, decoding differences were quantified as the mean of condition-level paired differences in RMSE between models,

$$\Delta {{\rm{RMSE}}}={{{\rm{RMSE}}}}_{{{\rm{baseline}}}}-{{{\rm{RMSE}}}}_{{{\rm{SNN}}}},$$

where the baseline corresponds to either the spike-count OLS LR decoder or the recurrent architectures (RNN and GRU), and the SNN denotes the spike-time–based decoding pipelines evaluated in this study. Differences were computed for each experimental condition (five finger tasks and two movement directions across two repetitions) and averaged to obtain a single effect size per subject.

Uncertainty was estimated using bootstrap resampling with 1000 samples at the repetition level. Repetitions were treated as the independent units, and all associated finger tasks and movement directions were retained within each resampled repetition. For SNN models, performance values were first averaged across the 10 random initialization seeds prior to effect-size computation. Ninety-five percent confidence intervals were obtained from the 2.5th and 97.5th percentiles of the resulting bootstrap distributions.

### Spiking neural network on asynchronous neuromorphic hardware

In this study, we demonstrate finger force decoding from motor units on DYNAP-SE mixed-signal neuromorphic processor^43^. The chip comprises 1024 analog neurons distributed across four cores of 256 neurons each, implementing the Adaptive Exponential Integrate-and-Fire (AdExp-IF) neuron model^57^. Networks can be connected through four possible synapses: two excitatory (AMPA- NMDA) and two inhibitory (GABA-A, GABA-B). Different types of synapses can be configured to express different temporal dynamics. Each neuron can receive a maximum of 64 incoming connections.

Configurable neuron parameters consist of the firing threshold, membrane time constant, refractory period and a gain factor. Synaptic parameters include the synaptic weight and time constant.

#### Topology

Bias parameters are expressed as currents, are set on-chip by the bias-generator circuit and are shared globally within each core. However, due to device mismatch, the effective values would differ within core^58^. This heterogeneity is exploited in decoding by replacing a single neuron per finger (as in simulation) with a small population of neurons. In this work, a population of five neurons was used for each finger direction. Decomposed motor units from each finger and direction served as input to a dedicated population of neurons on a selected core. All incoming connections were initialized with excitatory synapses, all-to-all connected to the neuronal population. External spike inputs are delivered to the chip via a dedicated FPGA interface using the address event representation (AER) protocol^43^.

#### Training with chip-in-the-loop

DYNAP-SE does not support on-chip learning. Synaptic connectivity was therefore trained using a chip-in-the-loop approach^59^, in which weight updates were computed off-chip based on spiking activity recorded from the hardware and subsequently applied to the device by updating the connectivity matrix.

At the start of training, neuronal and synaptic bias parameters were initialized and held constant throughout. During each training epoch, motor unit spike trains were delivered to their corresponding neuron populations, and the resulting output spikes were recorded from the chip and streamed back to the host computer. Output spike trains were temporally smoothed using an exponential kernel with a time constant of 200 ms. The smoothed activity was then used to compute the error associated with each input-output connection and to derive weight updates, Δ*w*, using a delta learning rule^60^:

5 $$\Delta {w}_{ij}=\eta \,{\left\langle \left({y}_{j}^{{{\rm{target}}}}(t)-{y}_{j}(t)\right)\cdot {x}_{i}(t)\right\rangle }_{{{\rm{t}}}},$$

where 〈⋅〉_t_ denotes temporal averaging over the duration of input presentation, *x*_*i*_(*t*) denotes the temporally smoothed presynaptic activity of motor unit *i*, *y*_*j*_(*t*) the temporally smoothed output activity of neuron *j*, ${y}_{j}^{{{\rm{target}}}}(t)$ the target finger force, and *η* = 0.001 the learning rate.

#### Synaptic efficacy update

Because synaptic weights are shared within each core, effective weight modulation was implemented by adjusting the number of physical synapses instantiated between input and output neurons, rather than by modifying continuous-valued parameters. Synaptic efficacy was therefore represented by discrete integer values: increasing the number of excitatory synapses strengthened the influence of a given motor unit on a neuron’s response, whereas reducing this number, or introducing inhibitory synapses, attenuated its contribution.

To map continuous-valued updates to discrete synaptic levels, we used stochastic rounding technique^61^. Each update was probabilistically rounded to the nearest lower or upper quantization level, with probabilities proportional to the fractional distance to each level.

Training was performed for a total of 10 epochs. Through this iterative process, network connectivity was progressively reshaped to produce population-level spiking activity that encoded force output.

#### Chip power consumption

As described in ref. ^62^, the power consumption consumption for a single population on DYNAP-SE can be estimated as:

6 $${P}_{{{\rm{pop}}}}={r}_{{{\rm{mu}}}}\,{N}_{{{\rm{mu}}}}\,{N}_{{{\rm{pop}}}}\,({E}_{{{\rm{spike}}}}+{E}_{{{\rm{pulse}}}})+{r}_{{{\rm{out}}}}\,{N}_{{{\rm{pop}}}}\,({E}_{{{\rm{en}}}}+{E}_{{{\rm{br}}}}),$$

where: *r*_mu_ = mean input firing rate of motor units to the population *r*_out_ = mean output firing rate of the population *N*_mu_ = number of input motor units to the population *N*_pop_ = number of post-synaptic neurons in the population *E*_spike_ = 883 pJ, energy to generate one spike *E*_pulse_ = 324 pJ, energy of the pulse extender circuit *E*_en_ = 883 pJ, energy to encode one spike and append destination *E*_br_ = 6.84 nJ, energy to broadcast events within the same core

#### Chip inference latency

Inference latency was computed as the time difference between the first input motor-unit spike and the first output spike emitted by the corresponding neuron population. This metric is fundamentally different from the wall-clock processing time typically reported for software implementations. Inference latency on neuromorphic hardware corresponds to the physical propagation and integration delay of spikes through the network. It is an event-driven latency that does not rely on fixed-time buffering and directly captures the end-to-end response time.

### Reporting summary

Further information on research design is available in the Nature Portfolio Reporting Summary linked to this article.

## Supplementary information

Supplementary Information

Reporting Summary

Transparent Peer Review file

## Source data

Source Data

## Data Availability

The motor unit decomposition data that support the findings of this study are publicly available on Figshare^63^. The original HD-iEMG recordings are not publicly available due to ethical, privacy considerations and ongoing research, but are available from the corresponding author (FB) upon reasonable request and subject to institutional approval. Source data are provided with this paper.

## Code availability

The code used to analyze the data and reproduce the main findings is available at https://github.com/FarahBaracat/snn-fingerforce-decoding^64^.

## References

[1] Parker, P., Stuller, J. & Scott, R. Signal processing for the multistate myoelectric channel. *Proc. IEEE* **65**, 662–674 (1977).

[2] Wołczowski, A. & Kurzyński, M. Human-machine interface in bioprosthesis control using EMG signal classification. *Expert Syst.* **27**, 53–70 (2010).

[3] Tam, W. -k, Wu, T., Zhao, Q., Keefer, E. & Yang, Z. Human motor decoding from neural signals: a review. *BMC Biomed. Eng.* **1**, 22 (2019). 32903354 10.1186/s42490-019-0022-z PMC7422484

[4] Day, S. J. & Hulliger, M. Experimental simulation of cat electromyogram: evidence for algebraic summation of motor-unit action-potential trains. *J. Neurophysiol.* **86**, 2144–2158 (2001). 11698507 10.1152/jn.2001.86.5.2144

[5] Parker, P. A. & Scott, R. N. Myoelectric control of prostheses. *Crit. Rev. Biomed. Eng.* **13**, 283–310 (1986). 3512166

[6] Fu, J., Choudhury, R., Hosseini, S. M., Simpson, R. & Park, J.-H. Myoelectric control systems for upper limb wearable robotic exoskeletons and exosuits—a systematic review. *Sensors* **22**, 8134 (2022). 36365832 10.3390/s22218134 PMC9655258

[7] Xiong, D. et al. Robotic telemanipulation with EMG-driven strategy-assisted shared control method. *Sci. China Technol. Sci.* **67**, 3812–3824 (2024).

[8] Lin, M., Huang, J., Fu, J., Sun, Y. & Fang, Q. A VR-based motor imagery training system with EMG-based real-time feedback for post-stroke rehabilitation. *IEEE Trans. Neural Syst. Rehabil. Eng.* **31**, 1–10 (2023). 36166567 10.1109/TNSRE.2022.3210258

[9] Even-Chen, N. et al. Power-saving design opportunities for wireless intracortical brain-computer interfaces. *Nat. Biomed. Eng.* **4**, 984–996 (2020). 32747834 10.1038/s41551-020-0595-9 PMC8286886

[10] Jiang, N., Dosen, S., Muller, K.-R. & Farina, D. Myoelectric control of artificial limbs—is there a need to change focus? [In the Spotlight]. *IEEE Signal Process. Mag.* **29**, 152–150 (2012).

[11] Boostani, R. & Moradi, M. H. Evaluation of the forearm EMG signal features for the control of a prosthetic hand. *Physiol. Meas.* **24**, 309 (2003). 12812417 10.1088/0967-3334/24/2/307

[12] Roche, A. D., Rehbaum, H., Farina, D. & Aszmann, O. C. Prosthetic myoelectric control strategies: a clinical perspective. *Curr. Surg. Rep.* **2**, 44 (2014).

[13] Jiang, N., Vest-Nielsen, J. L., Muceli, S. & Farina, D. EMG-based simultaneous and proportional estimation of wrist/hand kinematics in uni-lateral trans-radial amputees. *J. Neuroeng. Rehabil.* **9**, 42 (2012). 22742707 10.1186/1743-0003-9-42 PMC3546854

[14] Young, A. J., Smith, L. H., Rouse, E. J. & Hargrove, L. J. Classification of simultaneous movements using surface EMG pattern recognition. *IEEE Trans. Biomed. Eng.* **60**, 1250–1258 (2013). 23247839 10.1109/TBME.2012.2232293 PMC4208826

[15] Hahne, J. M. et al. Linear and nonlinear regression techniques for simultaneous and proportional myoelectric control. *IEEE Trans. Neural Syst. Rehabil. Eng.* **22**, 269–279 (2014). 24608685 10.1109/TNSRE.2014.2305520

[16] Del Vecchio, A. et al. Tutorial: analysis of motor unit discharge characteristics from high-density surface EMG signals. *J. Electromyogr. Kinesiol.* **53**, 102426 (2020). 32438235 10.1016/j.jelekin.2020.102426

[17] Keenan, K. G., Farina, D., Maluf, K. S., Merletti, R. & Enoka, R. M. Influence of amplitude cancellation on the simulated surface electromyogram. *J. Appl. Physiol.* **98**, 120–131 (2005). 15377649 10.1152/japplphysiol.00894.2004

[18] Thompson, C. K. et al. Robust and accurate decoding of motoneuron behaviour and prediction of the resulting force output. * J. Physiol.* **596**, 2643–2659 (2018). 29726002 10.1113/JP276153 PMC6046070

[19] Gazzoni, M., Farina, D. & Merletti, R. A new method for the extraction and classification of single motor unit action potentials from surface EMG signals. *J. Neurosci. Methods* **136**, 165–177 (2004). 15183268 10.1016/j.jneumeth.2004.01.002

[20] Holobar, A. & Zazula, D. Multichannel blind source separation using convolution kernel compensation. *IEEE Trans. Signal Process.* **55**, 4487–4496 (2007).

[21] Farina, D., Merletti, R. & Enoka, R. M. The extraction of neural strategies from the surface EMG: an update. *J. Appl. Physiol.* **117**, 1215–1230 (2014). 25277737 10.1152/japplphysiol.00162.2014 PMC4254845

[22] Kapelner, T. et al. Predicting wrist kinematics from motor unit discharge timings for the control of active prostheses. *J. Neuroeng. Rehabil.* **16**, 47 (2019). 30953528 10.1186/s12984-019-0516-x PMC6451263

[23] Xu, F., Zheng, Y. & Hu, X. Estimation of joint kinematics and fingertip forces using motoneuron firing activities: a preliminary report. In *2021 10th International IEEE/EMBS Conference on Neural Engineering (NER)*, 1035–1038 https://ieeexplore.ieee.org/document/9441433 (2021).

[24] Xia, M., Chen, C., Li, D., Sheng, X. & Ding, H. Simultaneous and proportional control of wrist and finger force via motor unit activity. *Biomed. Signal Process. Control* **103**, 107399 (2025).

[25] Duchateau, J. & Enoka, R. M. Human motor unit recordings: origins and insight into the integrated motor system. *Brain Res.* **1409**, 42–61 (2011). 21762884 10.1016/j.brainres.2011.06.011

[26] Maass, W. & Bishop, C. Pulsed neural networks (MIT Press, 1998).

[27] Gerstner, W. & Kistler, W. M. *Spiking Neuron Models: Single Neurons, Populations, Plasticity* (Cambridge University Press, 2002).

[28] Indiveri, G. & Sandamirskaya, Y. The importance of space and time for signal processing in neuromorphic agents. *IEEE Signal Process. Mag.* **36**, 16–28 (2019).

[29] Mead, C. Neuromorphic engineering: In Memory of Misha Mahowald. *Neural Comput.* **35**, 343–383 (2023). 36417590 10.1162/neco_a_01553

[30] Donati, E. & Indiveri, G. Neuromorphic bioelectronic medicine for nervous system interfaces: from neural computational primitives to medical applications. *Prog. Biomed. Eng.* **5**, 013002 (2023).

[31] Donati, E., Payvand, M., Risi, N., Krause, R. & Indiveri, G. Discrimination of EMG signals using a neuromorphic implementation of a spiking neural network. *Biomed. Circuits Syst. IEEE Trans.* **13**, 795–803 (2019). 10.1109/TBCAS.2019.2925454 31251192

[32] Sava, R., Donati, E. & Indiveri, G. Feed-forward and recurrent inhibition for compressing and classifying high dynamic range biosignals in spiking neural network architectures. In *Biomedical Circuits and Systems Conference (BioCAS)*10.1109/BioCAS58349.2023.10388963 (IEEE, 2023).

[33] Leroux, N., Finkbeiner, J. & Neftci, E. Online transformers with spiking neurons for fast prosthetic hand control https://arxiv.org/abs/2303.11860 (2023).

[34] Atzori, M. et al. Electromyography data for non-invasive naturally-controlled robotic hand prostheses. *Sci. Data* **1**, 140053 (2014). 25977804 10.1038/sdata.2014.53 PMC4421935

[35] Tanzarella, S., Iacono, M., Donati, E., Farina, D. & Bartolozzi, C. Neuromorphic decoding of spinal motor neuron behaviour during natural hand movements for a new generation of wearable neural interfaces. *IEEE Trans. Neural Syst. Rehabil. Eng.* **31**, 3035–3046 (2023). 37450365 10.1109/TNSRE.2023.3295658

[36] Muceli, S. et al. Accurate and representative decoding of the neural drive to muscles in humans with multi-channel intramuscular thin-film electrodes. * J. Physiol.* **593**, 3789–3804 (2015). 26174910 10.1113/JP270902 PMC4575568

[37] Grison, A. et al. A particle swarm optimised independence estimator for blind source separation of neurophysiological time series. *IEEE Trans. Biomed. Eng.* **72**, 227–237 (2025) 10.1109/TBME.2024.3446806 39167512

[38] Grison, A. et al. Intramuscular microelectrode arrays enable highly accurate neural decoding of finger tasks. *Interface Focus* **16**, 20250063 (2026).

[39] Rossato, J. et al. I-Spin live, an open-source software based on blind-source separation for real-time decoding of motor unit activity in humans. *eLife* **12**, RP88670 (2024). 39356736 10.7554/eLife.88670 PMC11446545

[40] Barsakcioglu, D. Y., Bräcklein, M., Holobar, A. & Farina, D. Control of spinal motoneurons by feedback from a non-invasive real-time interface. *IEEE Trans. Biomed. Eng.* **68**, 926–935 (2021). 32746024 10.1109/TBME.2020.3001942

[41] Grison, A. et al. Unlocking the full potential of high-density surface EMG: novel non-invasive high-yield motor unit decomposition. * J. Physiol.* **603**, 2281–2300 (2025). 40096591 10.1113/JP287913 PMC12013791

[42] Kline, J. C. & De Luca, C. J. Error reduction in EMG signal decomposition. *J. Neurophysiol.* **112**, 2718–2728 (2014). 25210159 10.1152/jn.00724.2013 PMC4254880

[43] Moradi, S., Qiao, N., Stefanini, F. & Indiveri, G. A scalable multicore architecture with heterogeneous memory structures for dynamic neuromorphic asynchronous processors (DYNAPs). *IEEE Trans. Biomed. Circuits Syst.* **12**, 106–122 (2018). 29377800 10.1109/TBCAS.2017.2759700

[44] Maryada et al. A canonical cortical electronic circuit for neuromorphic intelligence. *bioRxiv*10.1101/2025.03.28.646019 (2025).

[45] Biyan, Z., Sun, P.-S. V. & Basu, A. Combining SNNs with filtering for efficient neural decoding in implantable brain-machine interfaces. *Neuromorphic Comput. Eng.* **5**, 014013 (2025).

[46] Gallou, O. et al. Online epileptic seizure detection in long-term iEEG recordings using mixed-signal neuromorphic circuits. In *2024 IEEE Biomedical Circuits and Systems Conference (BioCAS)*, 1–5. 10.1109/BioCAS61083.2024.10798236(IEEE, 2024).

[47] Shaikh, S., So, R., Sibindi, T., Libedinsky, C. & Basu, A. Real-time closed loop neural decoding on a neuromorphic chip. In *2019 9th International IEEE/EMBS Conference on Neural Engineering (NER)*, 670–673 https://ieeexplore.ieee.org/document/8717122 (2019).

[48] Basu, A., Yi, C. & Enyi, Y. Big data management in neural implants: the neuromorphic approach. In *Emerging Technology and Architecture for Big-data Analytics* (eds. Chattopadhyay, A., Chang, C. H. & Yu, H.) 293–311 10.1007/978-3-319-54840-1_14 (Springer International Publishing, 2017).

[49] Farina, D., Merletti, R. & Enoka, R. M. The extraction of neural strategies from the surface EMG: 2004–2024. *J. Appl. Physiol.* **138**, 121–135 (2025). 39576281 10.1152/japplphysiol.00453.2024

[50] Varghese, R. J. et al. Design, fabrication and evaluation of a stretchable high-density electromyography array. *Sensors* **24**, 1810 (2024). 38544073 10.3390/s24061810 PMC10975572

[51] Baracat, F., Mazzoni, A., Micera, S., Indiveri, G. & Donati, E. Decoding gestures from intraneural recordings of a transradial amputee using event-based processing. https://www.techrxiv.org/users/722218/articles/707510-decoding-gestures-from-intraneural-recordings-of-a-transradial-amputee-using-event-based-processing?commit=d50ebbaffa67cbdac3d28112934866f542dea6a2 (2024).

[52] Richter, O. et al. DYNAP-SE2: a scalable multi-core dynamic neuromorphic asynchronous spiking neural network processor. *Neuromorphic Comput. Eng.* **4**, 014003 (2024).

[53] Greatorex, H. et al. A neuromorphic processor with on-chip learning for beyond-CMOS device integration. *Nat. Commun.* **16**, 6424 (2025). 10.1038/s41467-025-61576-6 PMC12254410 40645986

[54] Negro, F., Muceli, S., Castronovo, A. M., Holobar, A. & Farina, D. Multi-channel intramuscular and surface EMG decomposition by convolutive blind source separation. *J. Neural Eng.* **13**, 026027 (2016). 26924829 10.1088/1741-2560/13/2/026027

[55] Eshraghian, J. K. et al. Training spiking neural networks using lessons from deep learning. *Proc. IEEE*. **111**, 1016–1054 (2023).

[56] Yik, J. et al. The neurobench framework for benchmarking neuromorphic computing algorithms and systems. *Nat. Commun.* **16**, 1545 (2025). 10.1038/s41467-025-56739-4 PMC11814226 39934126

[57] Brette, R. & Gerstner, W. Adaptive exponential integrate-and-fire model as an effective description of neuronal activity. *J. Neurophysiol.* **94**, 3637–3642 (2005). 16014787 10.1152/jn.00686.2005

[58] Zendrikov, D., Solinas, S. & Indiveri, G. Brain-inspired methods for achieving robust computation in heterogeneous mixed-signal neuromorphic processing systems. *Neuromorphic Comput. Eng.* **3**, 034002 (2023).

[59] Baracat, F., Indiveri, G. & Donati, E. Finger force decoding from motor units activity on neuromorphic hardware. In *2025 IEEE Biomedical Circuits and Systems Conference (BioCAS)*, 636–640 (2025).

[60] Widrow, B. & Hoff, M. Adaptive switching circuits. In *1960 IRE WESCON Convention Record, Part 4*, 96–104, http://isl-www.stanford.edu/~widrow/papers/c1960adaptiveswitching.pdf (IRE, New York, 1960).

[61] Croci, M., Fasi, M., Higham, N. J., Mary, T. & Mikaitis, M. Stochastic rounding: implementation, error analysis and applications. *R. Soc. Open Sci.* **9**, 211631 (2022). 35291325 10.1098/rsos.211631 PMC8905452

[62] Risi, N., Aimar, A., Donati, E., Solinas, S. & Indiveri, G. A spike-based neuromorphic architecture of stereo vision. *Front. Neurorobot.* **14**, 93 (2020). 10.3389/fnbot.2020.568283 PMC7693562 33304262

[63] Baracat, F. Human motor units from high-density intramuscular microelectrode arrays. 10.6084/m9.figshare.31378327.v1 (2026).

[64] Baracat, F. Farahbaracat/snn-fingerforce-decoding: initial release: Snn finger force decoding. *Zenodo*10.5281/zenodo.19724261 (2026).

