# Supplementary material for: Spiking neural network decoders of finger forces from high-density intramuscular microelectrode arrays
Source: Nat Commun. 2026 Jun 24;17:7937. doi: 10.1038/s41467-026-74243-1 (PMC13447840; doi:10.1038/s41467-026-74243-1)
Supplement: Supplementary file 1 [file 41467_2026_74243_MOESM1_ESM.pdf]

Supplementary Information

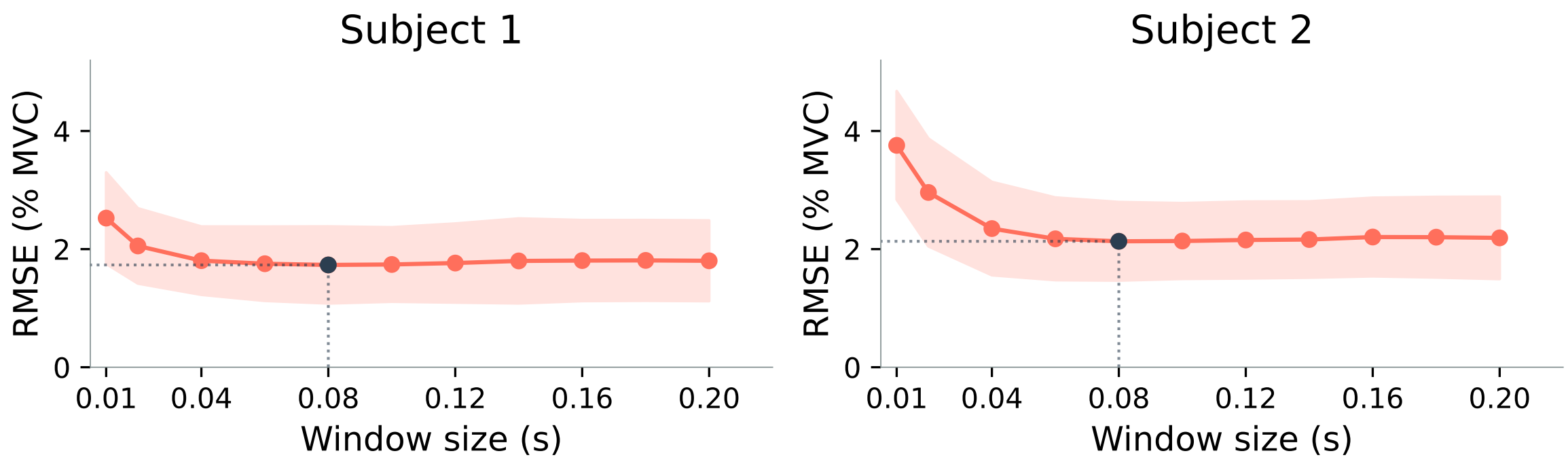

Fig. S1: Effect of analysis window length on decoding performance. Mean ± s.d. RMSE (%MVC) of the baseline ordinary least-squares linear regression (OLS LR) decoder as a function of window size, computed across finger tasks and movement directions. Motor unit spike counts were aggregated using overlapping windows (50% overlap). A window length of 80 ms (black marker) provided a favorable trade-off between decoding accuracy and temporal responsiveness and was therefore selected for all analyses reported in the main text.

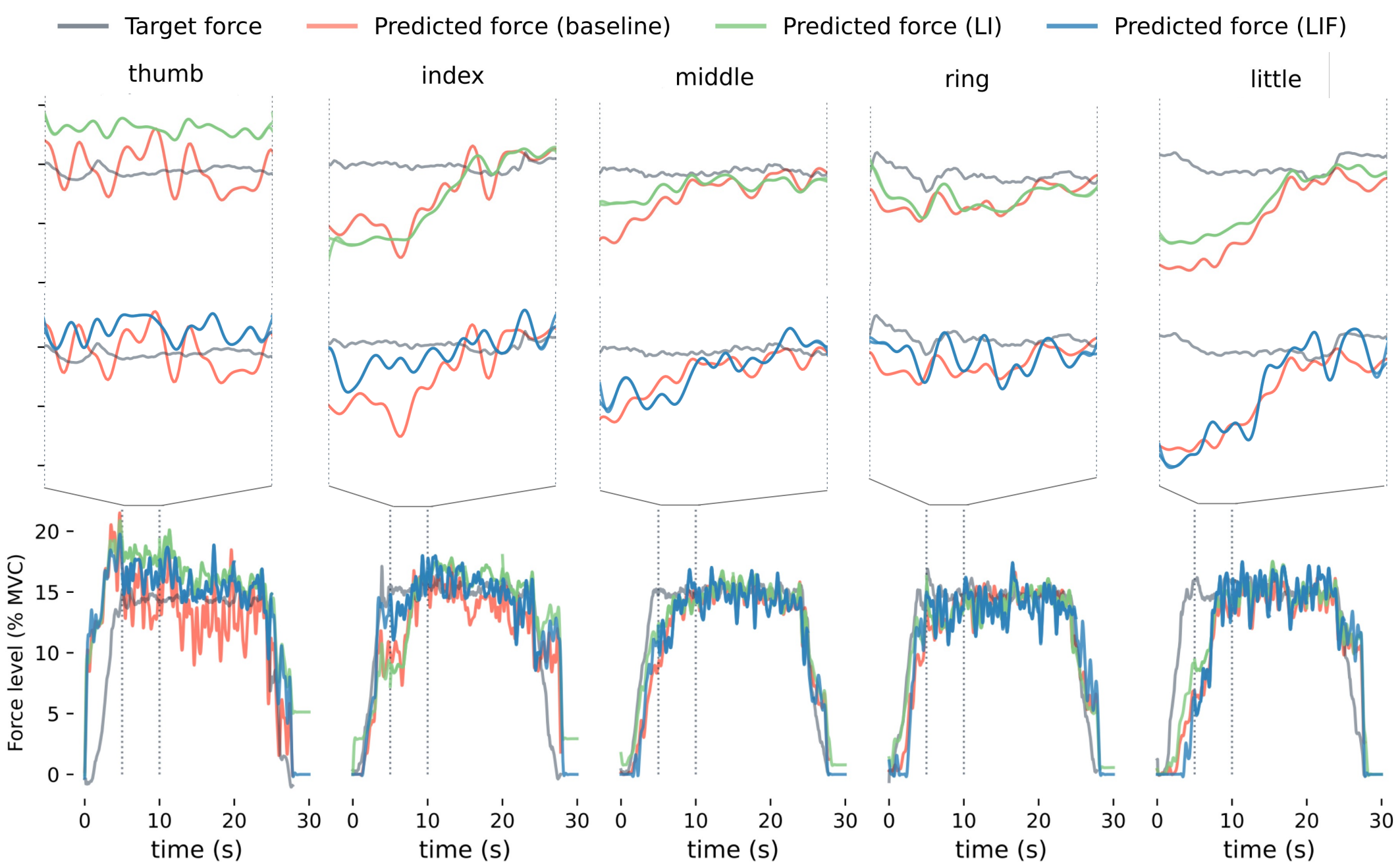

Fig. S2: Example force estimation traces for Subject 1 during individual finger extension tasks (repetition 2), using motor unit spike trains as inputs, with zoomed insets highlighting the interval between 5 and 10 seconds. Only the predicted forces for the active (targeted) finger are shown for clarity.

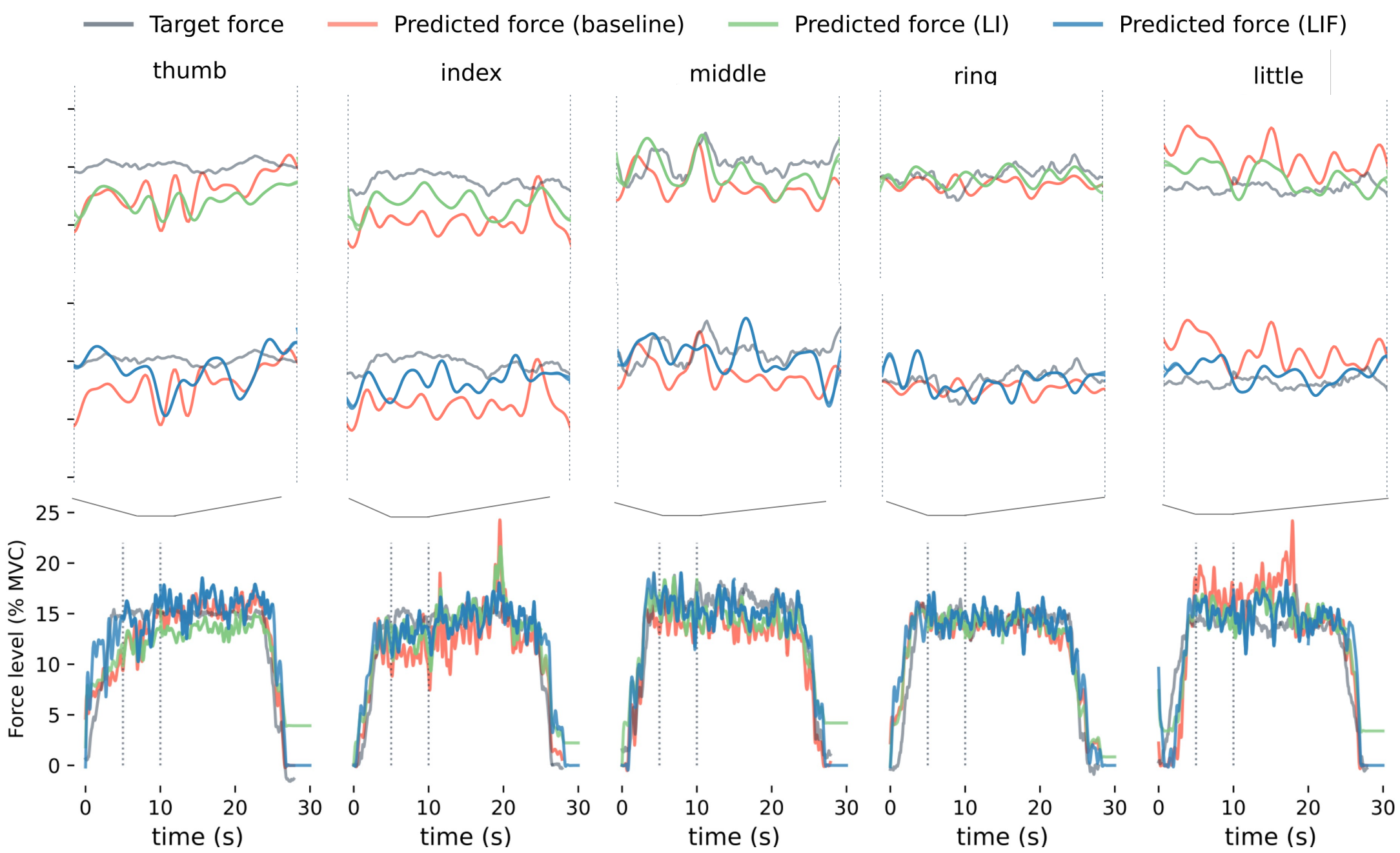

Fig. S3: Example force estimation traces for Subject 2 during individual finger extension tasks (repetition 2), using motor unit spike trains as inputs, with zoomed insets highlighting the interval between 5 and 10 seconds. Only the predicted forces for the active (targeted) finger are shown for clarity.

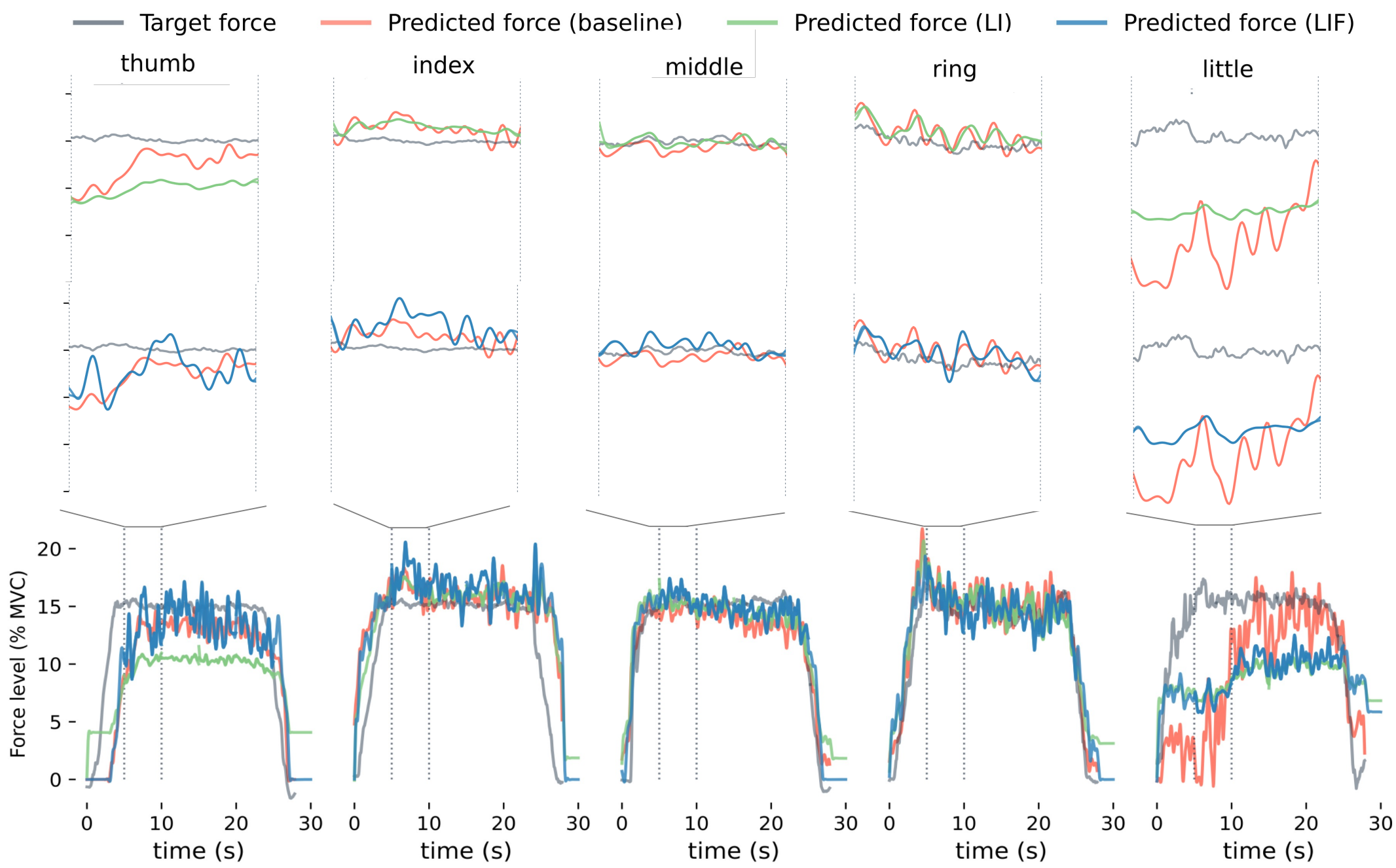

Fig. S4: Example force estimation traces for Subject 2 during individual finger flexion tasks (repetition 2), using motor unit spike trains as inputs, with zoomed insets highlighting the interval between 5 and 10 seconds. Only the predicted forces for the active (targeted) finger are shown for clarity.

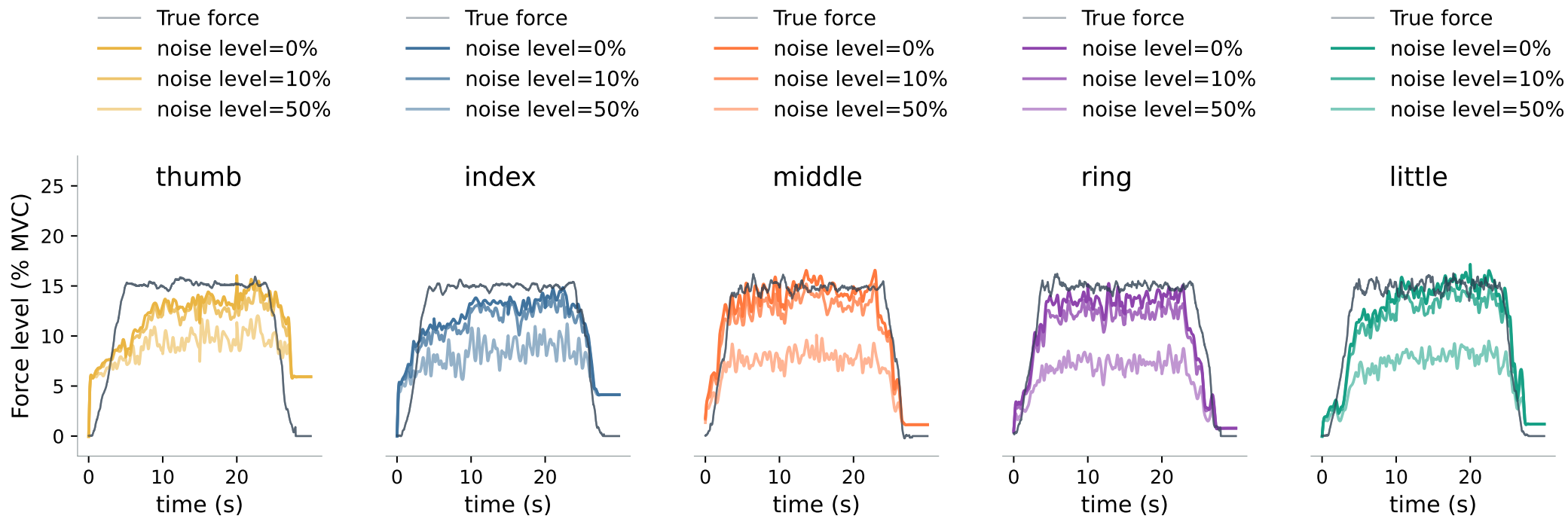

(a) SNN LI

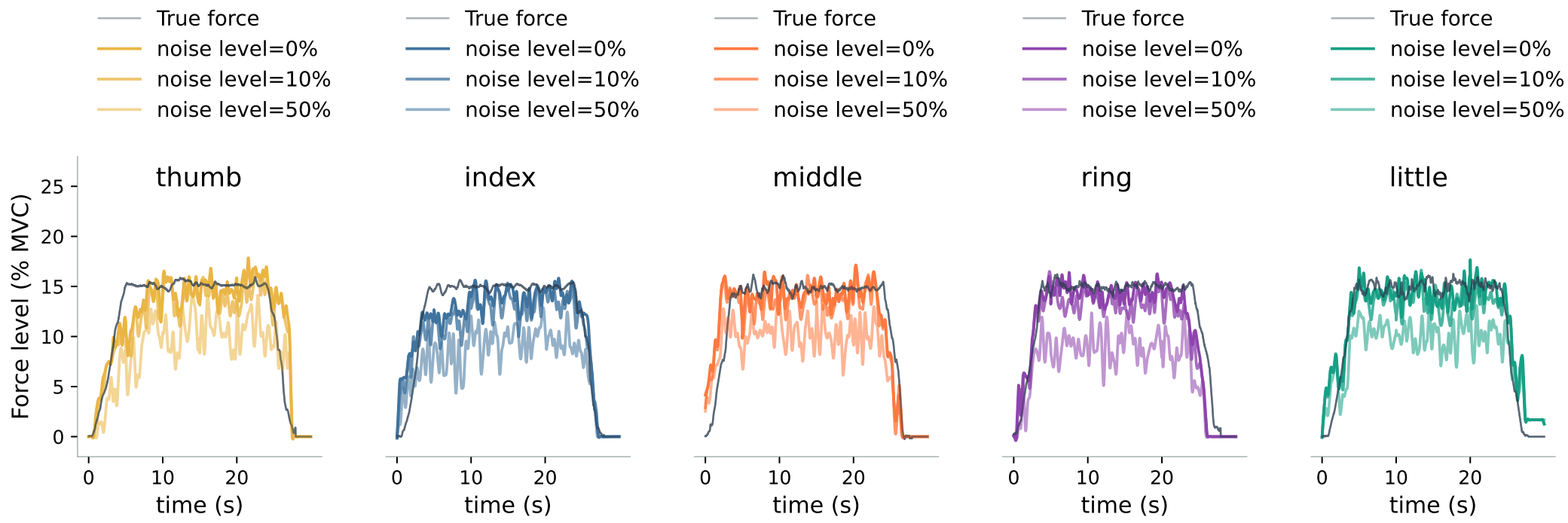

(b) SNN LIF

Fig. S5: **Example force estimations under spike omission decomposition noise.** Decoded forces for Subject 1 during flexion (repetition 1) under increasing levels of spike omission for the SNN models: (a) SNN LI, and (b) SNN LIF configurations.

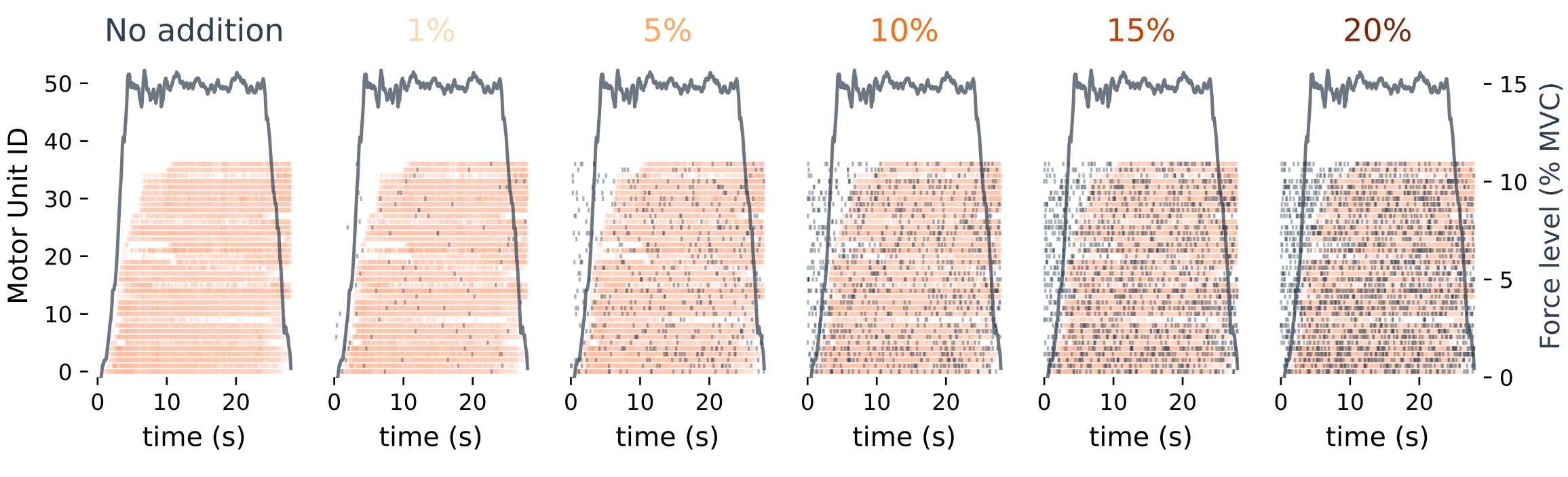

Fig. S6: Raster plots of decomposed motor unit spike trains from a representative trial (middle finger flexion, repetition 2, Subject 1) under increasing levels of false positive detections. False positives were inserted randomly during inference to simulate decomposition errors in real time and are shown in dark color. Overlaid force trajectories (%MVC) correspond to the target force level during the task.

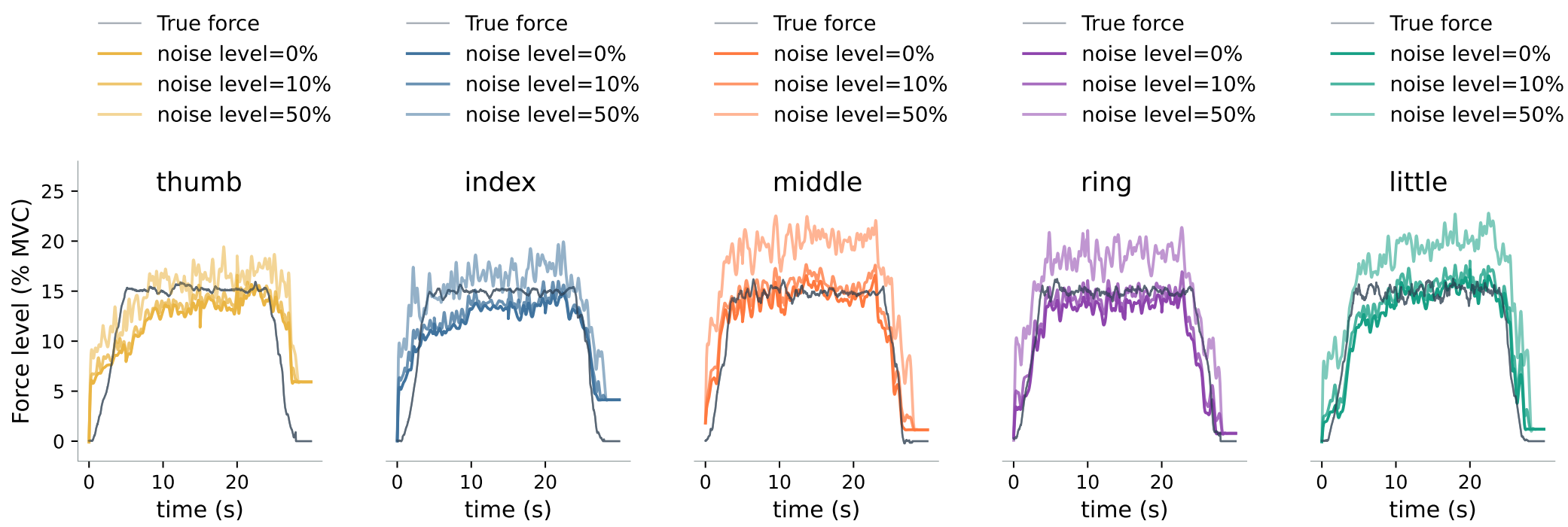

(a) SNN LI

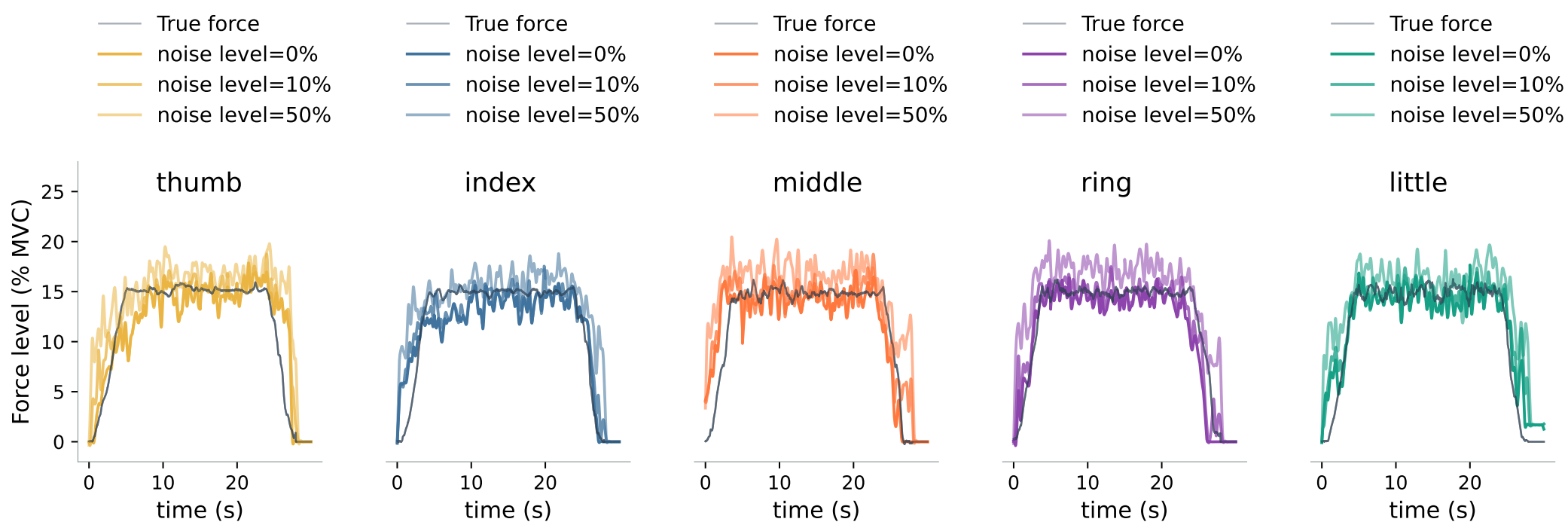

(b) SNN LIF

Fig. S7: **Example force estimations under spike addition (false positive) decomposition noise.** Decoded forces for Subject 1 during flexion (repetition 1) under increasing levels of false positives for the SNN models: (a) SNN LI, and (b) SNN LIF configurations.

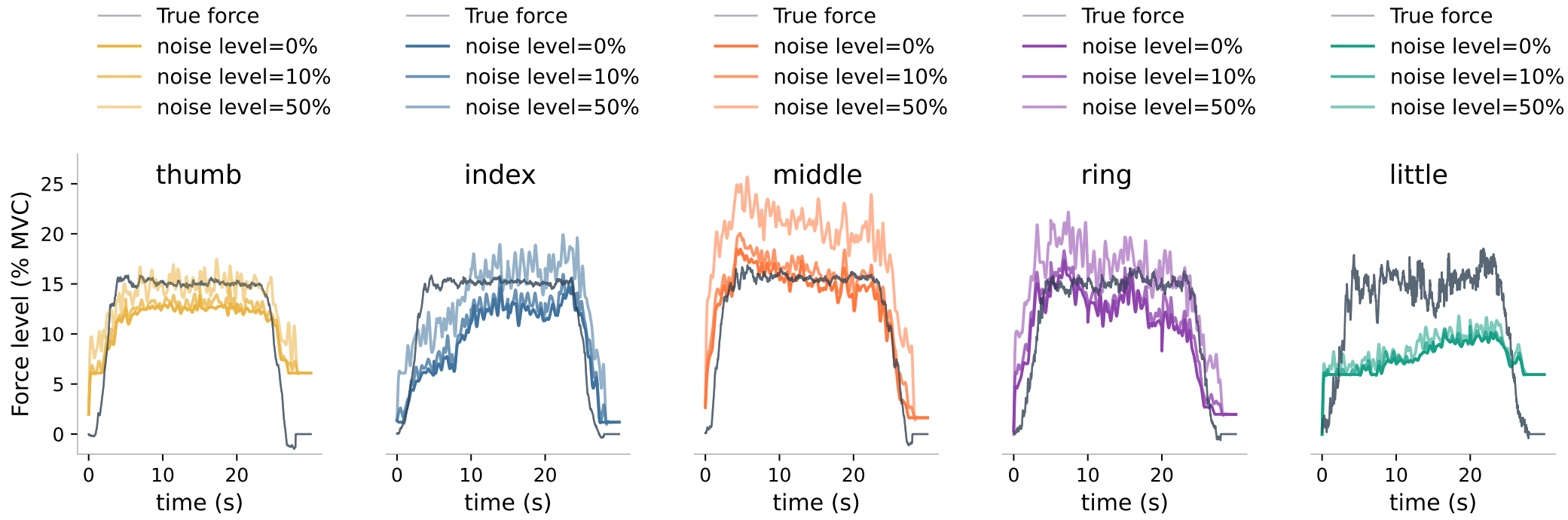

(a) SNN LI

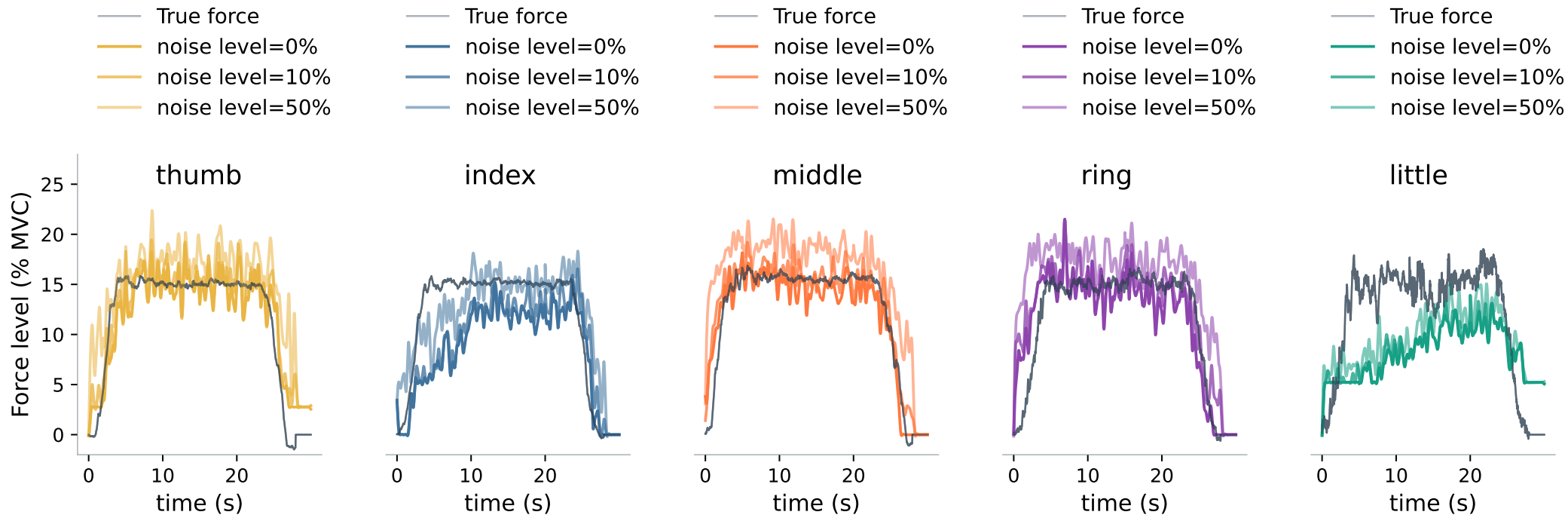

(b) SNN LIF

Fig. S8: **Example force estimations under spike addition (false positive) decomposition noise.** Decoded forces for Subject 2 during flexion (repetition 1) under increasing levels of false positives for the SNN models: (a) SNN LI, and (b) SNN LIF configurations.

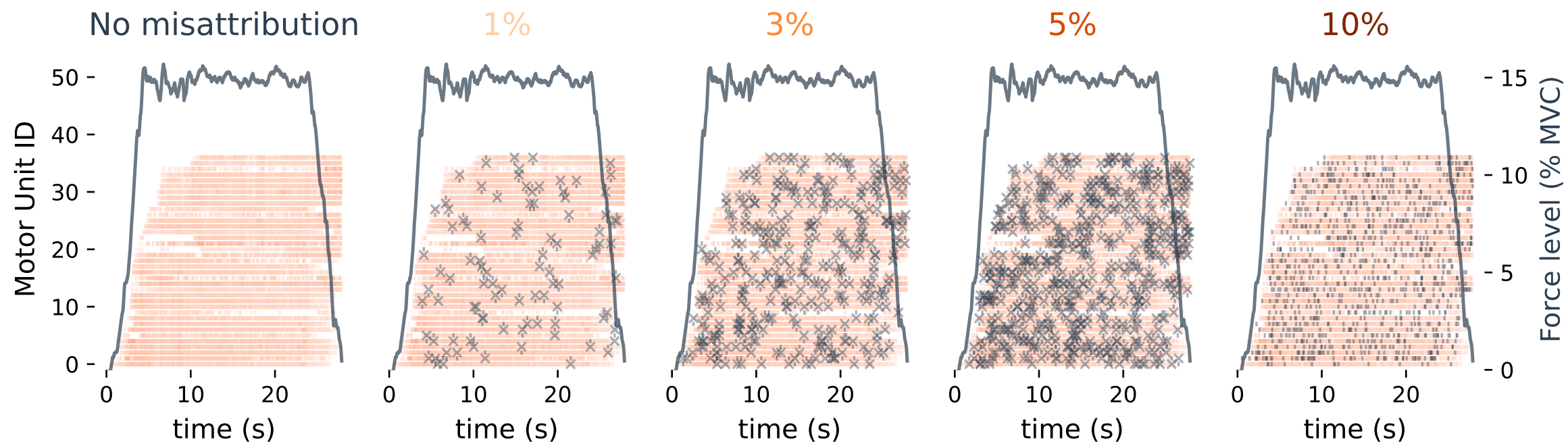

(a)

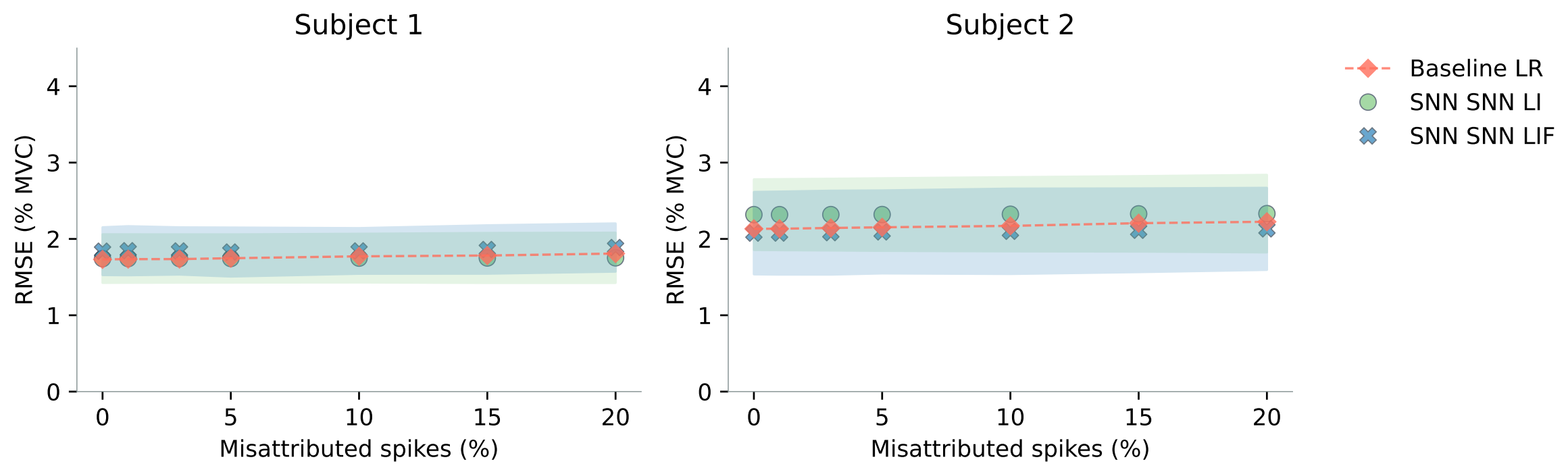

(b)

Fig. S9: **Robustness of SNN configurations to decomposition cross-motor units misattributions during inference.** (a) Raster plots of decomposed motor unit spike trains from a representative trial (middle finger flexion, repetition 2, Subject 1) under increasing levels of misattribution errors. During inference, a percentage of spike times were reassigned to randomly selected neighboring motor units. For misattribution erros below 10%, misattributed events are indicated by cross markers at their original source motor unit, while the corresponding reassigned events appear in the target motor units as dark bars. Overlaid force trajectories (%MVC) correspond to the target force level during the task. (b) Decoding RMSE across all tasks and movement directions for both subjects as a function of percentage of misattributions. Models were trained on clean motor unit spike trains and evaluated under varying levels of false positives. root mean squared errors (RMSEs) are presented as the aggregate mean values across tasks and movement directions, and shaded areas indicate ± standard deviation (SD). The standard deviation of the baseline model were omitted for clarity and are reported in Table S9.

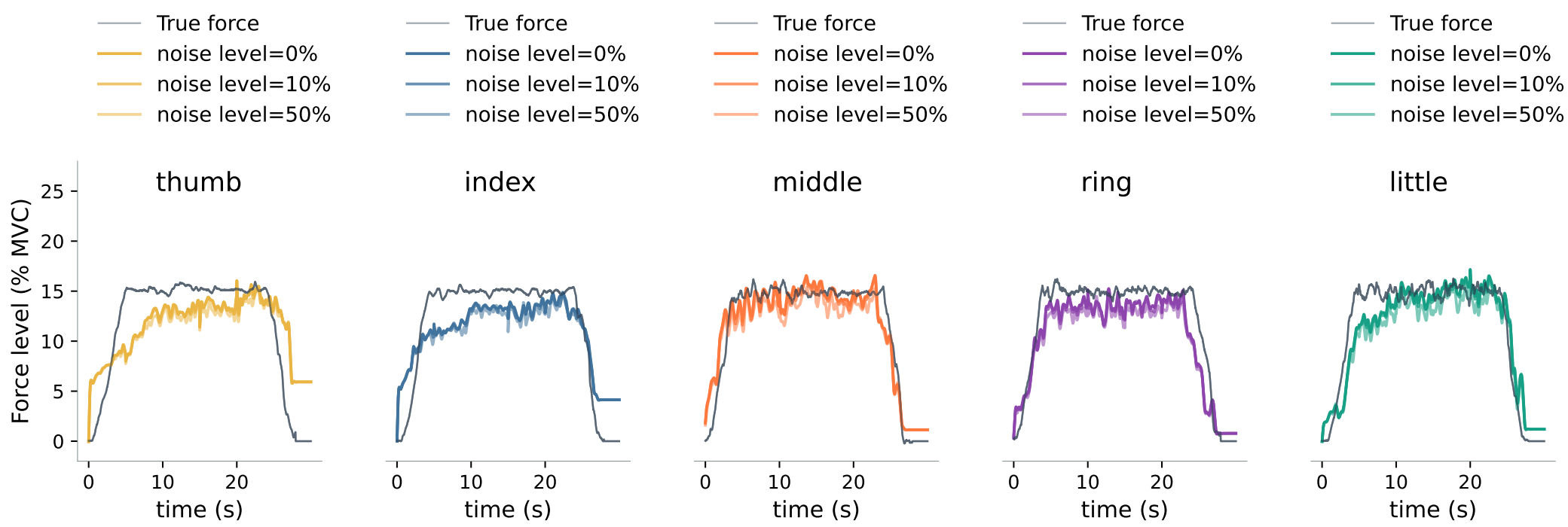

(a) SNN LI

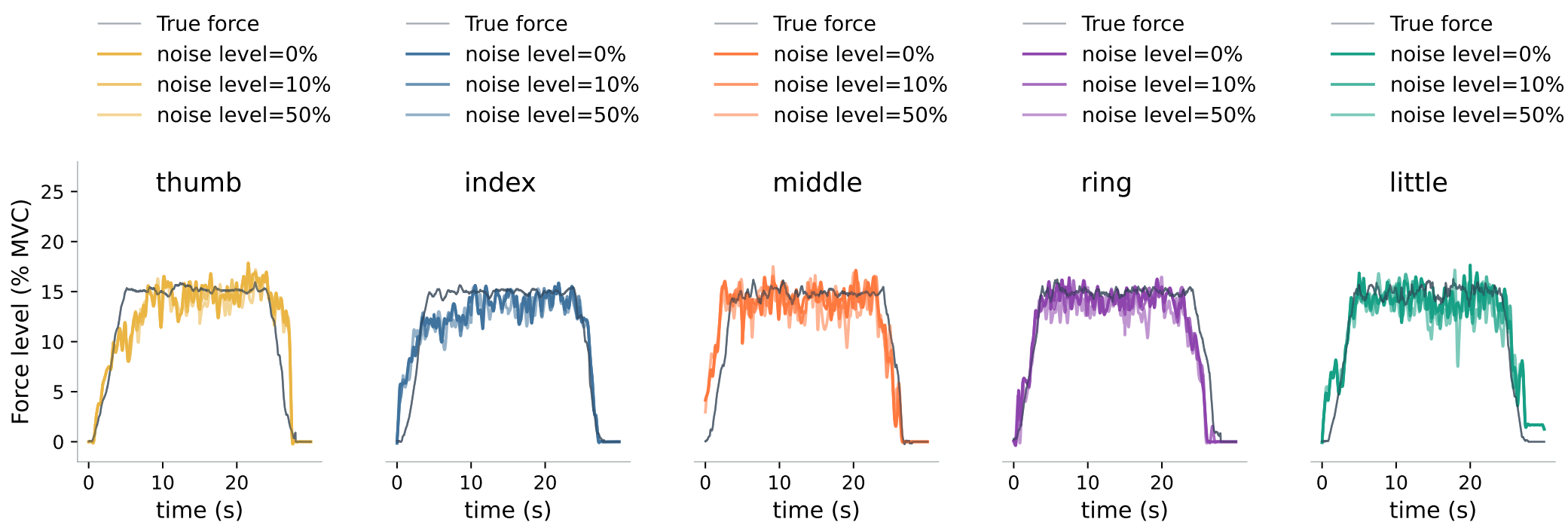

(b) SNN LIF

Fig. S10: **Example force estimations under cross-motor unit misattribution errors.** Decoded forces for Subject 1 during flexion (repetition 1) under increasing levels of misattributions for the SNN models: (a) SNN LI, and (b) SNN LIF configurations.

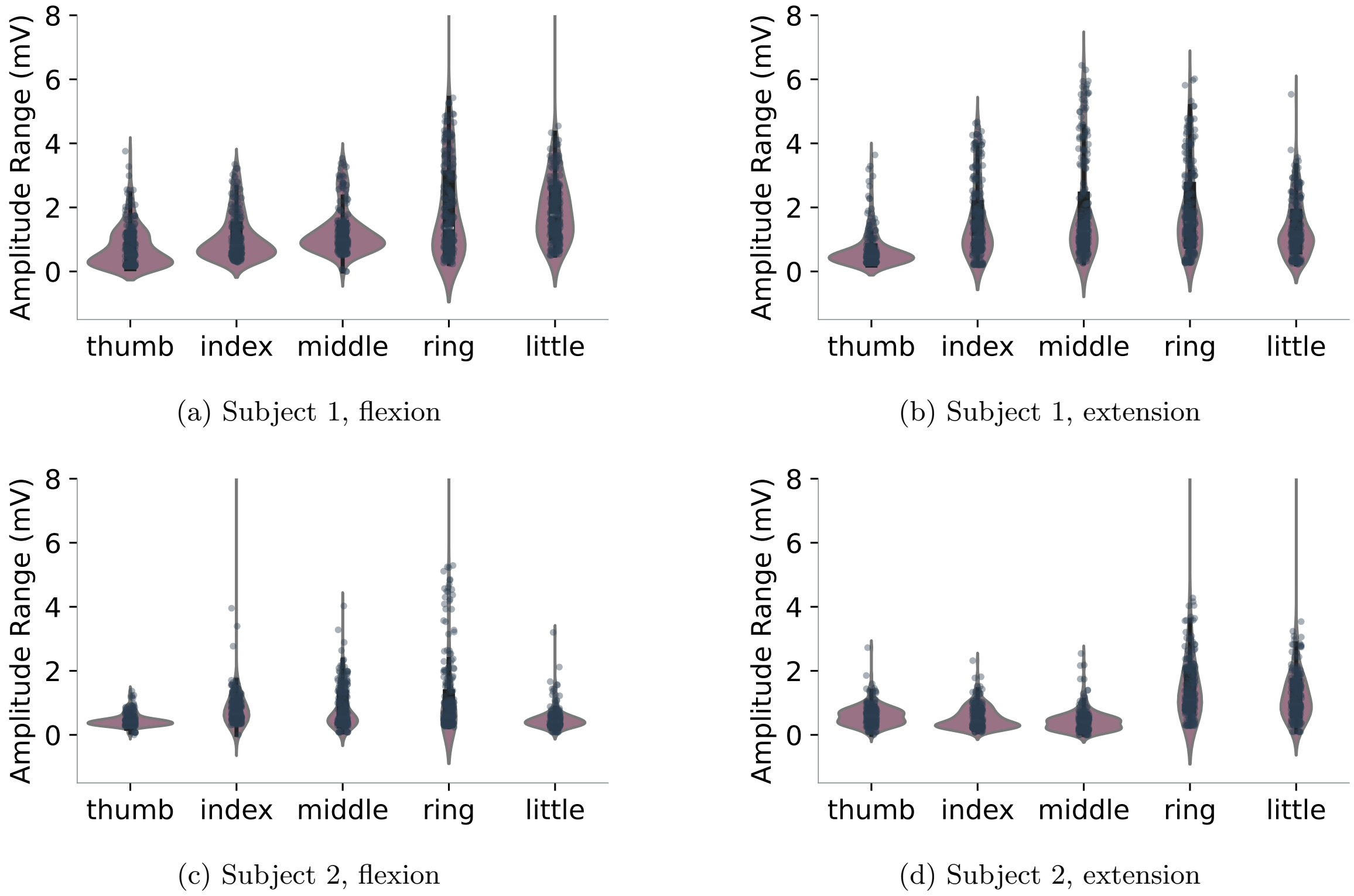

(a) Subject 1, flexion
(b) Subject 1, extension
(c) Subject 2, flexion
(d) Subject 2, extension

Fig. S11: HD-iEMG amplitude range distributions across tasks, repetitions, and movement directions. Violin plots show the distribution of peak-to-peak amplitude ranges (min–max, in mV) computed for each of the 120 HD-iEMG channels across repetitions and finger tasks. (a) Subject 1, flexion. (b) Subject 1, extension. (c) Subject 2, flexion. (d) Subject 2, extension. Individual points represent single-channel measurements (n=120 channel).

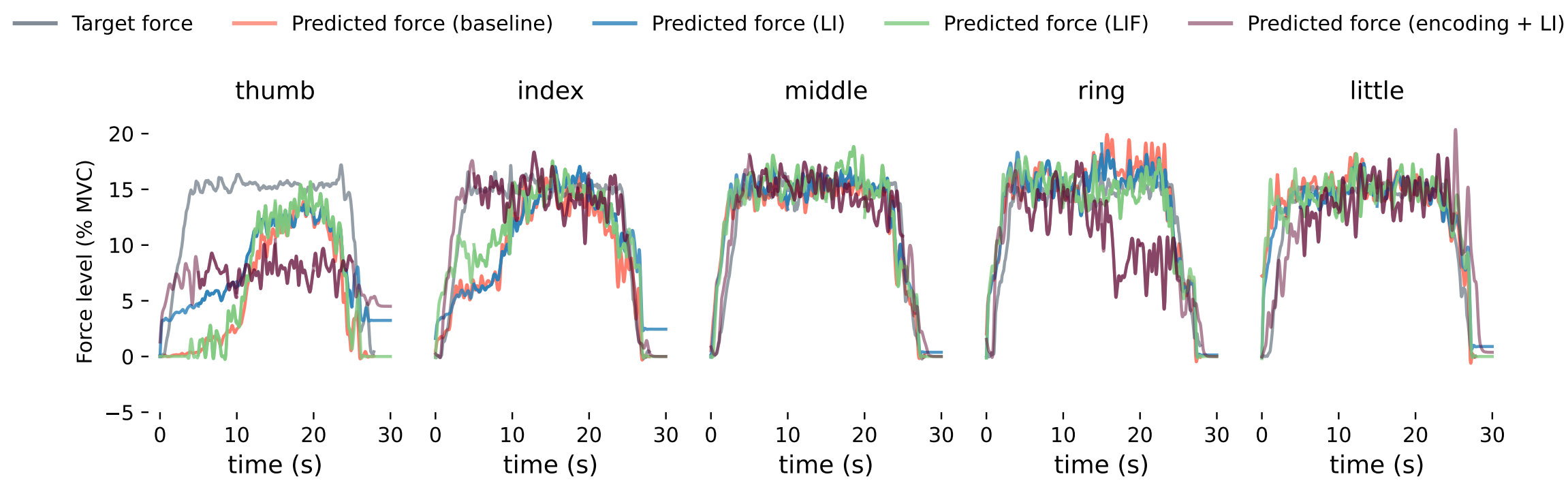

Fig. S12: Representative decoding of finger extension forces from spike-based inputs. Force estimation traces for Subject 1 (repetition 1) across decoder configurations. The LI (blue) and LIF (green) models received decomposed motor unit spike trains; the encoding + LI model (purple) used spike-encoded HD-iEMG; and the baseline linear regression decoder used binned spike counts of motor units as input.

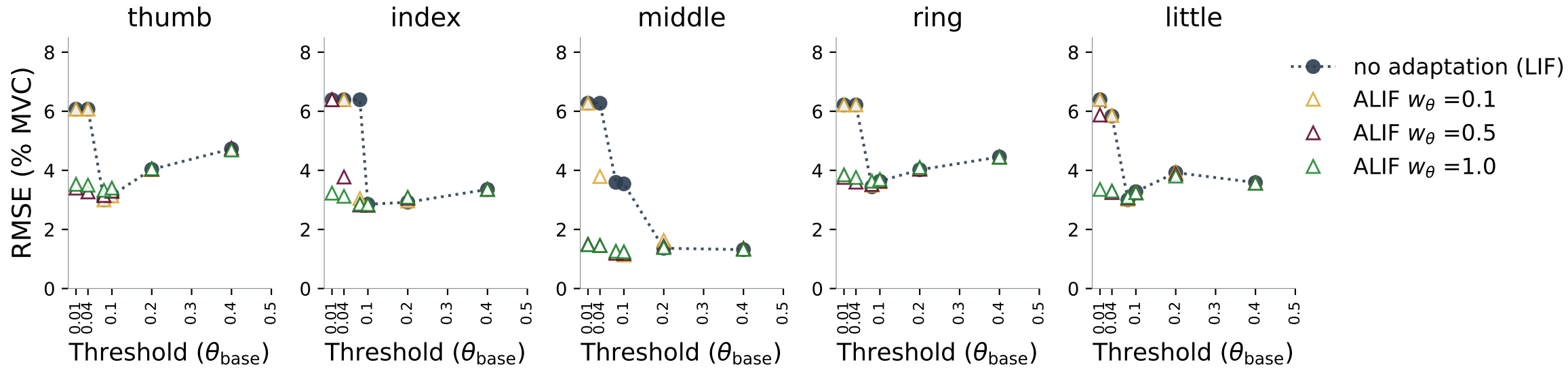

(a) Flexion

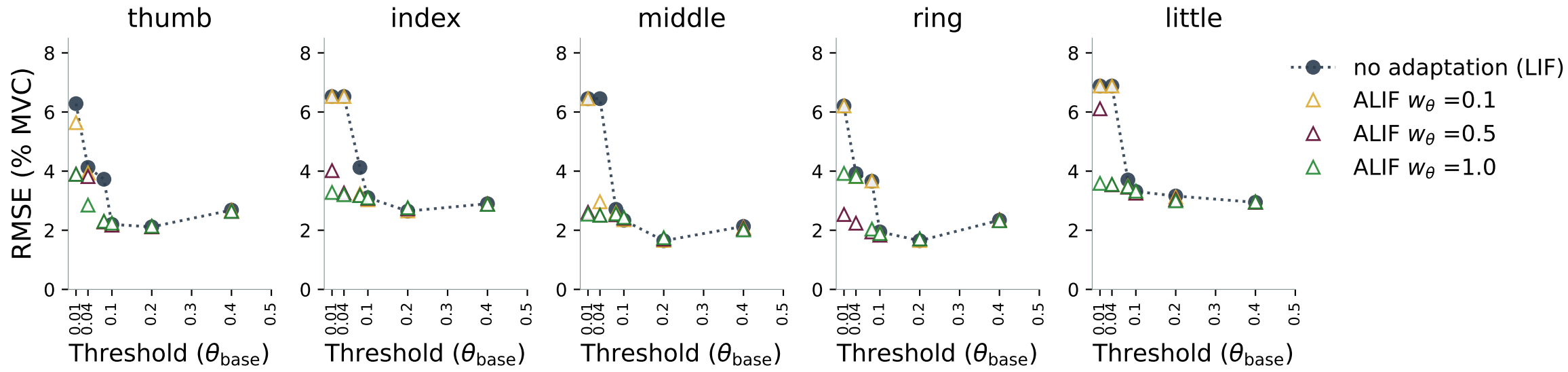

(b) Extension

Fig. S13: Sensitivity analysis of encoding neuron adaptation parameters. Effect of varying the baseline spiking threshold, θ_{base}, and the adaptation scale, w_θ, on decoding performance across finger tasks, measured in terms of RMSE (%MVC) for Subject 1. Results are reported for (a) flexion and (b) extension and compare non-adaptive LIF neurons with adaptive ALIF configurations. Markers indicate the average RMSE across cross-validation folds.

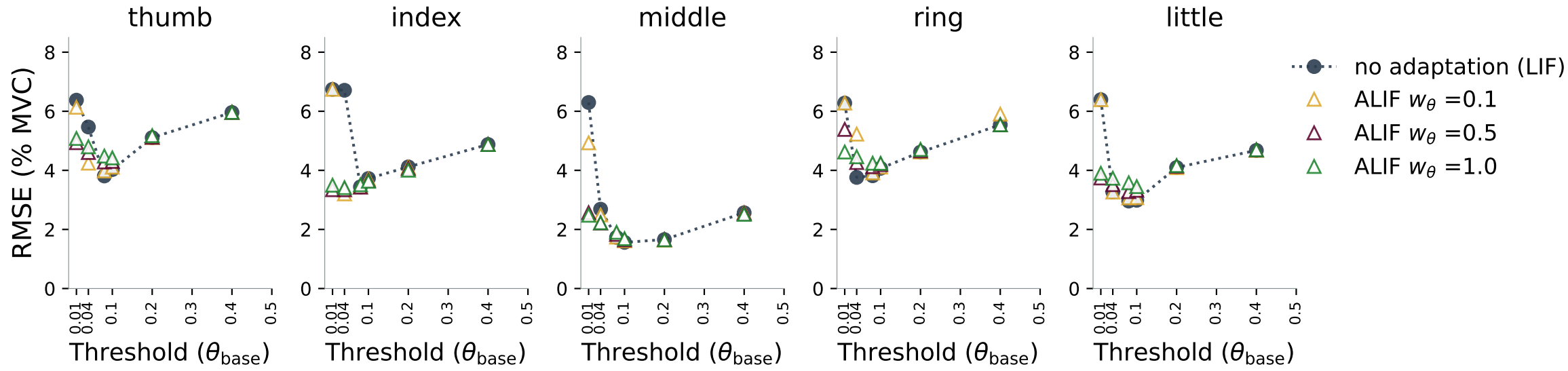

(a) Flexion

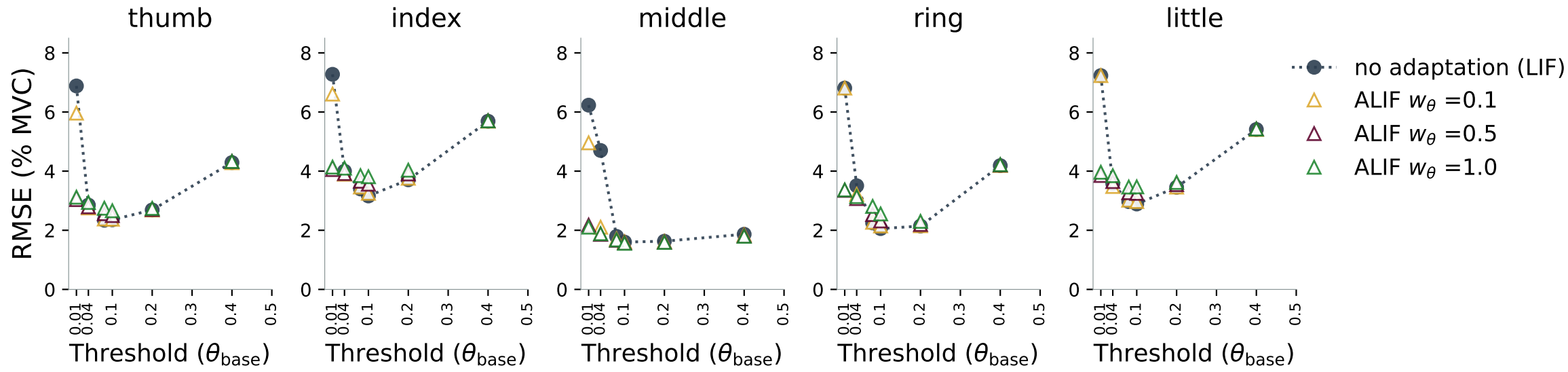

(b) Extension

Fig. S14: Sensitivity analysis of encoding neuron adaptation parameters. Effect of varying the baseline spiking threshold, θ_{base}, and the adaptation scale, w_θ, on decoding performance across finger tasks, measured in terms of RMSE (%MVC) for Subject 2. Results are reported for (a) flexion and (b) extension and compare non-adaptive LIF neurons with adaptive ALIF configurations. Markers indicate the average RMSE across cross-validation folds.

Table S1: Summary of baseline and SNN decoders' performance. Metrics are shown separately for extension and flexion, with bold entries representing the average across both directions. Results are averaged across 10 different initialization seeds chosen at random for the SNN and RNN decoders.

**Subject**	**Input Type**	**Decoder Model**	**Direction**	**RMSE** (%MVC)	**MAE** (%MVC)	$\mathbf{R^2}$
S1	motor unit spike count	Baseline (LR)	Flexion	1.31 ± 0.16	0.77 ± 0.08	0.94 ± 0.02
			Extension	2.16 ± 0.42	1.24 ± 0.19	0.83 ± 0.07
			**Avg.**	$\mathbf{1.73 \pm 0.29}$	$\mathbf{1.01 \pm 0.14}$	$\mathbf{0.88 \pm 0.04}$
	motor unit spike train	RNN (vanilla)	Flexion	1.88 ± 0.39	1.11 ± 0.20	0.87 ± 0.05
			Extension	2.17 ± 0.36	1.31 ± 0.17	0.83 ± 0.06
			**Avg.**	$\mathbf{2.02 \pm 0.37}$	$\mathbf{1.21 \pm 0.19}$	$\mathbf{0.85 \pm 0.05}$
		RNN(GRU)	Flexion	1.58 ± 0.23	1.05 ± 0.16	0.91 ± 0.02
			Extension	2.28 ± 0.32	1.52 ± 0.17	0.81 ± 0.05
			**Avg.**	$\mathbf{1.93 \pm 0.28}$	$\mathbf{1.29 \pm 0.17}$	$\mathbf{0.86 \pm 0.04}$
		SNN (LI)	Flexion	1.50 ± 0.30	0.96 ± 0.17	0.92 ± 0.03
			Extension	1.96 ± 0.16	1.22 ± 0.08	0.87 ± 0.02
			**Avg.**	$\mathbf{1.73 \pm 0.23}$	$\mathbf{1.09 \pm 0.13}$	$\mathbf{0.89 \pm 0.03}$
		SNN (LIF)	Flexion	1.48 ± 0.22	0.95 ± 0.20	0.92 ± 0.03
			Extension	2.08 ± 0.33	1.25 ± 0.22	0.85 ± 0.05
			**Avg.**	$\mathbf{1.78 \pm 0.27}$	$\mathbf{1.10 \pm 0.21}$	$\mathbf{0.88 \pm 0.04}$
	encoded HD-iEMG	SNN (ALIF-LI)	Flexion	2.87 ± 1.81	1.86 ± 1.20	0.62 ± 0.39
			Extension	2.66 ± 1.30	1.86 ± 0.89	0.71 ± 0.29
			**Avg.**	$\mathbf{2.76 \pm 1.57}$	$\mathbf{1.86 \pm 1.05}$	$\mathbf{0.66 \pm 0.35}$
S2	motor unit spike count	Baseline (LR)	Flexion	2.21 ± 0.35	1.35 ± 0.17	0.82 ± 0.05
			Extension	2.06 ± 0.29	1.37 ± 0.15	0.86 ± 0.04
			**Avg.**	$\mathbf{2.13 \pm 0.32}$	$\mathbf{1.36 \pm 0.16}$	$\mathbf{0.84 \pm 0.05}$
	motor unit spike train	RNN (vanilla)	Flexion	2.96 ± 0.86	1.94 ± 0.64	0.68 ± 0.16
			Extension	2.52 ± 0.36	1.72 ± 0.26	0.79 ± 0.05
			**Avg.**	$\mathbf{2.74 \pm 0.61}$	$\mathbf{1.83 \pm 0.45}$	$\mathbf{0.73 \pm 0.11}$
		RNN (GRU)	Flexion	2.27 ± 0.63	1.51 ± 0.57	0.81 ± 0.11
			Extension	2.14 ± 0.33	1.48 ± 0.22	0.85 ± 0.05
			**Avg.**	$\mathbf{2.21 \pm 0.48}$	$\mathbf{1.50 \pm 0.39}$	$\mathbf{0.83 \pm 0.08}$
		SNN (LI)	Flexion	2.47 ± 0.48	1.71 ± 0.40	0.79 ± 0.07
			Extension	2.16 ± 0.19	1.57 ± 0.17	0.86 ± 0.03
			**Avg.**	$\mathbf{2.31 \pm 0.34}$	$\mathbf{1.64 \pm 0.29}$	$\mathbf{0.82 \pm 0.05}$
		SNN (LIF)	Flexion	2.25 ± 0.60	1.50 ± 0.56	0.82 ± 0.09
			Extension	1.99 ± 0.21	1.41 ± 0.19	0.88 ± 0.03
			**Avg.**	$\mathbf{2.12 \pm 0.41}$	$\mathbf{1.46 \pm 0.38}$	$\mathbf{0.85 \pm 0.06}$
	encoded HD-iEMG	SNN (ALIF-LI)	Flexion	3.34 ± 1.10	2.37 ± 0.75	0.61 ± 0.22
			Extension	2.48 ± 1.04	1.89 ± 0.90	0.79 ± 0.16
			**Avg.**	$\mathbf{2.91 \pm 1.15}$	$\mathbf{2.13 \pm 0.86}$	$\mathbf{0.70 \pm 0.21}$

Supplementary Methods

1 Decoders Footprint

The reported decoder footprint accounts for the total number of parameters in the processing pipeline, including zero-valued parameters. Since all parameters are represented at full precision (4 bytes), the memory footprint scales directly with the model parameter count.

For the linear regression (LR), the parameters consist exclusively of the linear regression coefficients. Given M decomposed motor units and $N_{\text{out}} = 5$ output variables, and assuming no intercept term, the total number of parameters is given by

$$P_{\text{LR}} = M \cdot N_{\text{out}} \tag{1}$$

For the RNN-based decoders, the parameter count includes both the preprocessing stage and the recurrent decoding layer. The convolutional preprocessing stage applies an exponential kernel of temporal length k ($k = 5$) independently to each of the M motor units and therefore introduces $k \cdot M$ kernel parameters. The recurrent layer further contains input-to-hidden and hidden-to-hidden weight matrices, without bias terms. For a recurrent layer with H hidden units ($H = 5$), the total number of parameters for a vanilla RNN is therefore given by

$$P_{\text{RNN(vanilla)}} = k \cdot M + H \cdot M + H^2 \tag{2}$$

GRU has three sets of weight matrices, each characterized by distinct input-to-hidden and hidden-to-hidden transformations. Without considering additional bias terms, and including a fully connected projection layer to the output ($N_{\text{out}} = 5$), the total number of parameters for the GRU is given by

$$P_{\text{RNN(GRU)}} = k \cdot M + 3\,(H \cdot M + H^2) + H \cdot N_{\text{out}} \tag{3}$$

For the SNN LI configuration, the network parameters comprise the input-to-output weight matrix, bias terms, and fixed synaptic and membrane time constants. Accordingly, the total number of parameters is given by

$$P_{\text{SNN(LI)}} = M \cdot N_{\text{out}} + N_{\text{out}} + 2 \tag{4}$$

In contrast to the LI configuration, the SNN LIF architecture includes an intermediate spiking layer with trainable membrane time constants (one per output neuron, $N_{\text{out}} = 5$) and fixed firing thresholds. The input projects to this layer through synaptic connections of size $M \cdot N_{\text{out}} + N_{\text{out}}$, characterized by a fixed synaptic time constant. The parameter count associated with this first stage is therefore given by

$$P_{\text{LIF},1} = M \cdot N_{\text{out}} + 2N_{\text{out}} + 2, \tag{5}$$

where the constant term accounts for the fixed firing threshold and synaptic time constant.

This spiking layer is followed by a layer of leaky neurons with fixed membrane time constants and a one-to-one connectivity pattern. However, for consistency in footprint estimation, the full projection matrix, including zero-valued entries, is considered and has size $N_{\text{out}} \times N_{\text{out}}$. Finally, a second filtering stage is applied using leaky neurons with fixed time constants. The parameters associated with these subsequent stages therefore amount to

$$P_{\text{LIF},2} = N_{\text{out}}^2 + 2, \tag{6}$$

where the constant term accounts for the fixed membrane time constants of the leaky neurons in the two filtering stages.

For the SNN architecture with an encoding layer, an initial stage maps the HD-iEMG signals into spike trains. This encoding layer consists of one neuron per electromyography (EMG) channel, denoted

by N_{EMG}. Although the functional connectivity is one-to-one, for consistency in footprint estimation the full connection matrix of size $N_{\mathrm{EMG}} \times N_{\mathrm{EMG}}$ is considered.

Each encoding neuron is characterized by a fixed membrane time constant, an adaptive firing threshold defined independently for each channel, and a global threshold adaptation time constant. The parameter count associated with the encoding stage is therefore given by

$$P_{\mathrm{Enc},1} = N_{\mathrm{EMG}}^2 + N_{\mathrm{EMG}} + 2 \tag{7}$$

The encoding layer is followed by a fully connected layer of leaky neurons. This stage therefore introduces a weight matrix of size $N_{\mathrm{EMG}} \times N_{\mathrm{out}}$ and fixed membrane and synaptic time constants. Finally, a second filtering stage is applied using leaky neurons with fixed time constants. The parameters associated with these subsequent stages therefore amount to

$$P_{\mathrm{Enc},2} = N_{\mathrm{EMG}} \cdot N_{\mathrm{out}} + 3, \tag{8}$$

where the constant term account for the fixed neuron and synaptic time constants of the leaky and filtering layers. Since this architecture relies directly on HD-iEMG signals as input, in contrast to the other models described, its parameter count is identical for both flexion and extension directions, as it does not depend on the number of decomposed motor units.

Table S2: Static metrics summary. Reported total parameter counts include the linear coefficients for the baseline model, and the synaptic weights, biases, and time constants for neurons and synapses in the SNN architectures. Connection sparsity in only measured for the SNN pipelines.

**Subject**	**Decoder**	**Direction**	**Total Param./ Conn. Sparsity**	**Trainable Param.**	**Param. Memory (Bytes)**	**Decomposition Memory (Bytes)**
S1	OLS LR	Flex.	605	605	2420	58080
		Ext.	980	980	3920	94080
	RNN (vanilla)	Flex.	1235	630	4940	58080
		Ext.	1985	1005	7940	94080
	RNN (GRU)	Flex.	2520	1915	10080	58080
		Ext.	4020	3040	16080	94080
	SNN (LI)	Flex.	612 / 0	610	2448	58080
		Ext.	987 / 0	985	3948	94080
	SNN (LIF)	Flex.	644 / 0.0317	615	2576	58080
		Ext.	1019 / 0.0199	990	4076	94080
	SNN (ALIF)	Flex./Ext.	15125 / 0.9520	605	60500	0
S2	OLS LR	Flex.	255	255	1020	24480
		Ext.	465	465	1860	44640
	RNN (vanilla)	Flex.	535	280	2140	24480
		Ext.	955	490	3820	44640
	RNN (GRU)	Flex.	1120	865	4480	24480
		Ext.	1960	1495	7840	44640
	SNN (LI)	Flex.	262 / 0	260	1048	24480
		Ext.	472 / 0	470	1888	44640
	SNN (LIF)	Flex.	294 / 0.0714	265	1176	24480
		Ext.	504 / 0.0408	475	2016	44640
	SNN (ALIF)	Flex./Ext.	15125 / 0.9520	605	60500	0

2 Pipelines Computational Costs

We estimate the average computational cost required to process a single input sample for each decoding pipeline evaluated in this study, as summarized in Fig. S15. For pipelines operating on decomposed motor-unit activity, computation naturally separates into distinct stages. First, motor-unit based approaches rely on an initial *decomposition* stage that extracts motor unit spike trains from multi-channel HD-iEMG. We therefore begin by quantifying the inference-time cost of this decomposition step, assuming a fixed and optimized separation matrix. The remaining stages of each pipeline are then analyzed separately, with costs attributed either to preprocessing of the motor unit spike trains or directly to the decoding model, depending on the architecture.

For M motor units, N_{EMG} input EMG channels, and T discrete time steps per input sample, the number of multiply–accumulate (MAC) operations required to obtain the motor unit spike trains is

$$\text{MAC}_{\text{decomposition}} = M \cdot N_{\text{EMG}} \cdot T \tag{9}$$

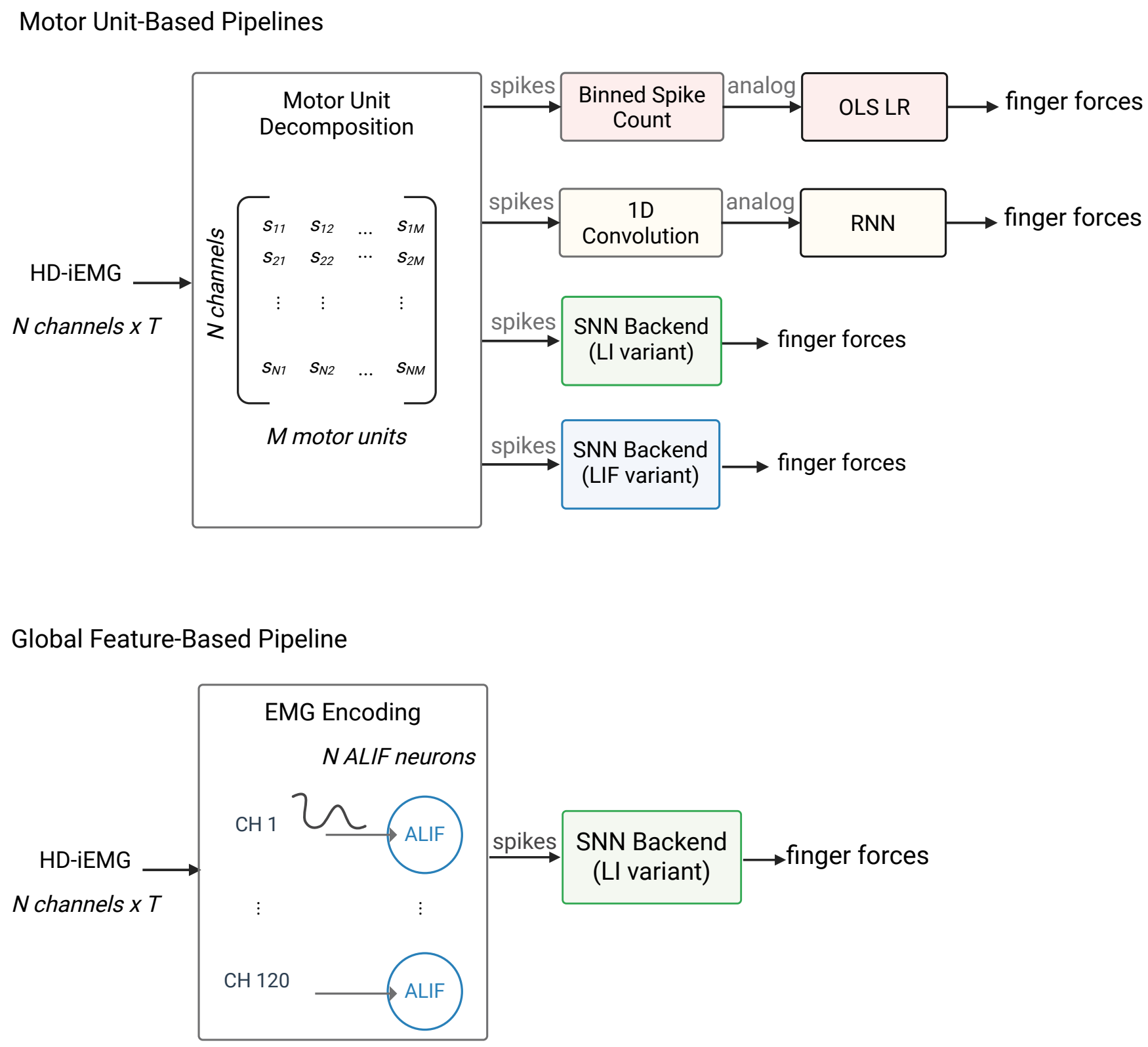

Fig. S15: Schematic of decoding pipelines evaluated in this study using either decomposed motor unit spike trains (top), or spike-encoded HD-iEMG (bottom). Created in BioRender. Baracat, F. (2026) https://BioRender.com/lpx613v.

Following decomposition, the OLS LR pipeline comprises two sequential stages: a preprocessing stage, in which motor-unit spike trains are transformed into binned spike-count features over overlapping temporal windows of 80 ms, and a linear regression decoding stage that maps these features to

the output variables. The preprocessing stage involves accumulate (AC) operations proportional to the total number of detected spikes for each sample across all input motor units.

The computational cost of the regression stage depends on the number of decomposed motor units, the number of output variables, and the number of temporal bins. Let B denote the number of bins per input sample, M the number of decomposed motor units, and $N_{\text{out}} = 5$ the number of output variables. For each bin, the regression stage performs a matrix–vector multiplication between the M-dimensional input vector and the regression coefficients. The total number of MAC operations required to process one input sample is therefore given by

$$\text{MAC}_{\text{LR}} = B \cdot M \cdot N_{\text{out}} \tag{10}$$

For the Recurrent Neural Networks (RNNs), we breakdown the contributions of the preprocessing and decoder stages. Let M denote the number of decomposed motor units, T the number of discrete time steps per input sample, and k the temporal length of the exponential convolution kernel. Each motor unit is independently convolved over the full sample duration, resulting in a total number of MAC operations for the convolutional stage equal to

$$\text{MAC}_{\text{conv}} = k \cdot M \cdot T \tag{11}$$

The computational cost of the recurrent decoding stage depends on the specific RNN architecture and its internal state dimensionality. Let H denote the number of hidden units in the recurrent layer. For the vanilla RNN, the number of MAC operations required to update the hidden state at each time step scales with $H^2 + HM$. For the GRU, three gated transformations (update, reset, and candidate state) are computed at each time step. Over the full input sequence, this results in

$$\text{MAC}_{\text{RNN(vanilla)}} = T \cdot (H^2 + HM) \tag{12}$$

$$\text{MAC}_{\text{RNN(GRU)}} = 3T \cdot (H^2 + HM) \tag{13}$$

The total computational cost of the baseline RNN decoders is therefore given by

$$\text{MAC}_{\text{total}} = \text{MAC}_{\text{conv}} + \text{MAC}_{\text{RNN}} \tag{14}$$

For the SNN, as defined by the NeuroBench framework suite [1], the computational cost associated with processing a single input sample is decomposed into *dense* and *effective* synaptic operations (synaptic operations (SynOps)). This distinction enables the separation of architecture-dependent theoretical complexity from activity-dependent realized cost.

Dense SynOps quantify the total number of potential synaptic interactions implied by the network topology and the input sequence length, independently of the actual neural activity. They account for all possible synaptic operations, including those associated with zero-valued inputs and inactive neurons, and therefore provide a hardware-agnostic upper bound on the computational workload. For a single layer with M input channels, T discrete time steps per input sample, and N_{out} output neurons, the dense synaptic operations are given by

$$\text{SynOp}_{\text{dense}} = N_{\text{out}} \cdot M \cdot T \tag{15}$$

In contrast, *effective* SynOps capture the operations that are actually executed during inference and depend on the input representation. When a layer is driven by spike trains, SynOps are triggered only upon the occurrence of presynaptic spikes and correspond to effective AC operations, exploiting both temporal sparsity and binary-valued activity. Conversely, for encoding layers operating on continuous-valued signals, SynOps are implemented as MAC operations.

More concretely, for spike-driven layers, effective SynOps are computed directly from the presynaptic spike activity. For the LI SNN, the presynaptic layer consists of M motor units projecting to $N_{\text{out}} = 5$ output neurons. Let $\mathbf{s}(t) \in \{0,1\}^M$ denote the motor-unit spike vector at time step t. The total number of presynaptic spikes over an input sample of length T is

$$N_{\text{spk}} = \sum_{t=1}^{T} \sum_{m=1}^{M} s_m(t). \tag{16}$$

Since each presynaptic spike induces synaptic updates on all outgoing connections, the total number of spike-driven synaptic operations for one input sample is

$$\text{AC}_{\text{Eff}-\text{LI}} = N_{\text{spk}} \cdot N_{\text{out}} = 5\, N_{\text{spk}}. \tag{17}$$

Table S3: Computational cost and resource breakdown for Spiking Neural Networks (SNNs). Dense SynOps (MOp) are computed without accounting for input sparsity. Effective SynOps (kOp) account only for synaptic events triggered by pre-synaptic activity. Spike sparsity is defined as $\frac{N_{\text{spk}}}{T\,M}$, where T denotes the number of time steps per input sample and M the number of motor units. Peak/Avg memory and CPU time are reported per input sample during inference.

**Subject**	**Decoder**	**Dir.**	**Dense SynOps (MOp)**	**Eff. SynOps (kOp)**	**Spike Sparsity**	**Peak/Avg. Memory (MiB)**	**Peak/Avg. CPU Time (s)***
S1	SNN (LI)	Flex.	1.690	34.28	0.0192	3.82/3.82	0.434/0.427
		Ext.	2.737	63.60	0.0164	5.43/5.42	0.443/0.426
	SNN (LIF)	Flex.	1.760	35.28	0.0192	5.60/5.59	0.979/0.935
		Ext.	2.807	64.60	0.0164	7.21/7.20	1.055/0.946
	SNN (ALIF)	Flex.	41.895	847.00	0.1037	64.97/64.88	1.511/1.306
		Ext.	41.895	894.00	0.1008	65.17/64.90	1.624/1.324
S2	SNN (LI)	Flex.	0.712	13.19	0.0051	2.33/2.33	1.320/0.617
		Ext.	1.299	28.08	0.0122	3.23/3.22	0.439/0.429
	SNN (LIF)	Flex.	0.782	14.19	0.0051	4.12/4.11	1.105/0.954
		Ext.	1.369	29.08	0.0122	5.00/4.99	0.958/0.927
	SNN (ALIF)	Flex.	41.895	740.00	0.0183	64.98/64.68	1.327/1.288
		Ext.	41.895	785.00	0.0742	64.90/64.72	1.324/1.278

*CPU time corresponds to the wall-clock processing time required to process a full input sample (i.e., an entire repetition).

Table S4: Computational costs and resource breakdown for the OLS LR linear regression models. Operations are reported as the average per input sample (during inference). Binning operations are calculated based on the average total number of spikes per input sample. Costs are split by direction, as the number of motor units involved in each direction varies.

Subject	Direction	Bin. ACs (kOp)	LR MACs (kOp)	Total FLOPs (kOp)	Avg. Memory (MiB)	Peak / Avg. CPU Time (ms)
S1	Flex.	13.663	421.685	857.033	33.44	0.442 / 0.264
	Ext.	27.578	683.060	1393.698	33.44	0.549 / 0.347
S2	Flex.	5.368	177.735	360.838	33.44	0.382 / 0.261
	Ext.	10.958	324.105	659.168	33.44	0.439 / 0.251

Table S5: Computational costs and ressources breakdown for RNN decoders. Operation counts are reported as the average per input sample during inference (forward pass only). Decoder costs are expressed in floating-point operations (FLOPs), while convolutional costs are computed in MACs.

Subject	Decoder	Direction	Conv. MACs (MOp)	RNN FLOPs (MOp)	Total FLOPs (MOp)	Peak/Avg. Memory (MiB)	Peak/Avg. CPU Time (ms)*
S1	vanilla	Flex.	1.69	3.52	6.90	10.83/10.83	20.939/19.893
		Ext.	2.74	5.61	11.09	17.23/17.02	24.004/21.338
	GRU	Flex.	1.69	10.71	14.09	12.90/11.75	57.997/56.659
		Ext.	2.74	17.00	22.48	18.04/18.03	57.962/56.238
S2	vanilla	Flex.	0.71	3.13	4.55	4.86/4.86	21.214/19.222
		Ext.	1.30	5.47	8.08	8.45/8.45	20.498/19.990
	GRU	Flex.	0.71	9.69	11.12	6.20/5.71	61.805/57.402
		Ext.	1.30	16.73	19.33	9.25/9.23	59.844/56.633

*CPU time corresponds to the wall-clock processing time required to process a full input sample (i.e., an entire repetition).

Table S6: Average test performance metrics for each decoder model under increasing spike omission levels. Values are reported as means with standard deviations, computed across finger tasks and movement directions.

**Subject**	**Decoder Model**	**Omission Level (%)**	**RMSE** (%MVC)	**MAE** (%MVC)	$\mathbf{R}^2$
S1	LR	0	$\mathbf{1.73 \pm 0.26}$	1.01 ± 0.18	0.88 ± 0.04
		10	1.90 ± 0.29	1.11 ± 0.22	0.86 ± 0.05
		20	2.20 ± 0.32	1.28 ± 0.23	0.82 ± 0.05
		30	2.60 ± 0.32	1.48 ± 0.24	0.76 ± 0.06
		40	3.04 ± 0.31	1.70 ± 0.26	0.67 ± 0.07
		50	3.53 ± 0.27	1.93 ± 0.29	0.56 ± 0.06
	SNN LI	0	1.74 ± 0.32	1.10 ± 0.17	0.89 ± 0.04
		10	$\mathbf{1.84 \pm 0.33}$	1.18 ± 0.21	0.88 ± 0.04
		20	2.07 ± 0.33	1.34 ± 0.25	0.85 ± 0.04
		30	2.40 ± 0.27	1.54 ± 0.26	0.80 ± 0.04
		40	2.79 ± 0.23	1.77 ± 0.29	0.74 ± 0.04
		50	3.22 ± 0.22	2.01 ± 0.31	0.65 ± 0.04
	SNN LIF	0	1.84 ± 0.31	1.13 ± 0.25	0.88 ± 0.05
		10	1.88 ± 0.34	1.18 ± 0.26	0.87 ± 0.05
		20	$\mathbf{1.98 \pm 0.35}$	1.26 ± 0.28	0.86 ± 0.05
		30	$\mathbf{2.16 \pm 0.37}$	1.39 ± 0.28	0.83 ± 0.06
		40	$\mathbf{2.43 \pm 0.36}$	1.55 ± 0.29	0.79 ± 0.07
		50	$\mathbf{2.81 \pm 0.40}$	1.76 ± 0.29	0.72 ± 0.08
S2	LR	0	2.13 ± 0.54	1.36 ± 0.45	0.84 ± 0.08
		10	2.29 ± 0.57	1.47 ± 0.50	0.81 ± 0.09
		20	2.56 ± 0.57	1.65 ± 0.53	0.77 ± 0.09
		30	2.94 ± 0.55	1.86 ± 0.53	0.71 ± 0.10
		40	3.38 ± 0.52	2.11 ± 0.56	0.62 ± 0.11
		50	3.85 ± 0.48	2.36 ± 0.59	0.51 ± 0.11
	SNN LI	0	2.32 ± 0.46	1.64 ± 0.40	0.82 ± 0.06
		10	2.41 ± 0.54	1.73 ± 0.40	0.81 ± 0.08
		20	2.59 ± 0.57	1.88 ± 0.42	0.78 ± 0.08
		30	2.85 ± 0.54	2.07 ± 0.43	0.74 ± 0.09
		40	3.16 ± 0.48	2.27 ± 0.45	0.68 ± 0.09
		50	3.52 ± 0.42	2.50 ± 0.47	0.60 ± 0.08
	SNN LIF	0	$\mathbf{2.08 \pm 0.54}$	1.42 ± 0.49	0.85 ± 0.07
		10	$\mathbf{2.14 \pm 0.55}$	1.48 ± 0.51	0.85 ± 0.08
		20	$\mathbf{2.26 \pm 0.55}$	1.58 ± 0.52	0.83 ± 0.08
		30	$\mathbf{2.46 \pm 0.58}$	1.73 ± 0.56	0.80 ± 0.09
		40	$\mathbf{2.74 \pm 0.55}$	1.92 ± 0.57	0.75 ± 0.10
		50	$\mathbf{3.11 \pm 0.55}$	2.15 ± 0.58	0.68 ± 0.11

Bold values indicate the best-performing models (lowest mean RMSE) at each omission level for each subject.

Table S7: Average test performance metrics for each decoder model under increasing spike addition (false-positive) levels for Subject 1. Values are reported as means with standard deviations, computed across finger tasks and movement directions.

**Subject**	**Decoder Model**	**Addition Level (%)**	**RMSE** (%MVC)	**MAE** (%MVC)	$\mathbf{R}^2$
S1	LR	0	$\mathbf{1.73 \pm 0.26}$	1.01 ± 0.18	0.88 ± 0.04
		1	$\mathbf{1.73 \pm 0.26}$	1.00 ± 0.18	0.88 ± 0.04
		2	$\mathbf{1.73 \pm 0.26}$	1.01 ± 0.18	0.88 ± 0.04
		3	$\mathbf{1.73 \pm 0.27}$	1.01 ± 0.19	0.88 ± 0.04
		4	$\mathbf{1.73 \pm 0.28}$	1.01 ± 0.20	0.88 ± 0.04
		5	$\mathbf{1.73 \pm 0.29}$	1.01 ± 0.20	0.88 ± 0.04
		10	$\mathbf{1.77 \pm 0.31}$	1.05 ± 0.23	0.88 ± 0.05
		15	1.86 ± 0.36	1.10 ± 0.27	0.87 ± 0.05
		20	1.97 ± 0.40	1.17 ± 0.32	0.85 ± 0.06
		30	2.28 ± 0.45	1.36 ± 0.41	0.80 ± 0.08
		40	2.66 ± 0.49	1.58 ± 0.49	0.73 ± 0.09
		50	3.07 ± 0.58	1.81 ± 0.57	0.64 ± 0.13
	SNN LI	0	1.74 ± 0.32	1.10 ± 0.17	0.89 ± 0.04
		1	1.74 ± 0.32	1.10 ± 0.17	0.89 ± 0.04
		2	1.74 ± 0.32	1.10 ± 0.17	0.89 ± 0.04
		3	1.74 ± 0.31	1.10 ± 0.16	0.89 ± 0.04
		4	1.74 ± 0.31	1.10 ± 0.16	0.89 ± 0.04
		5	1.74 ± 0.31	1.10 ± 0.16	0.89 ± 0.03
		10	$\mathbf{1.77 \pm 0.28}$	1.12 ± 0.16	0.89 ± 0.03
		15	$\mathbf{1.83 \pm 0.24}$	1.16 ± 0.19	0.88 ± 0.03
		20	$\mathbf{1.92 \pm 0.20}$	1.22 ± 0.23	0.87 ± 0.03
		30	$\mathbf{2.15 \pm 0.24}$	1.35 ± 0.31	0.83 ± 0.04
		40	2.45 ± 0.40	1.52 ± 0.41	0.78 ± 0.07
		50	2.78 ± 0.57	1.70 ± 0.51	0.72 ± 0.11
	SNN LIF	0	1.84 ± 0.31	1.13 ± 0.25	0.88 ± 0.05
		1	1.84 ± 0.31	1.13 ± 0.25	0.88 ± 0.05
		2	1.85 ± 0.31	1.14 ± 0.25	0.88 ± 0.04
		3	1.85 ± 0.29	1.14 ± 0.25	0.87 ± 0.04
		4	1.86 ± 0.29	1.15 ± 0.24	0.87 ± 0.04
		5	1.86 ± 0.28	1.15 ± 0.24	0.87 ± 0.04
		10	1.90 ± 0.28	1.18 ± 0.27	0.87 ± 0.04
		15	1.95 ± 0.27	1.22 ± 0.29	0.86 ± 0.04
		20	2.02 ± 0.27	1.27 ± 0.32	0.85 ± 0.04
		30	2.16 ± 0.31	1.36 ± 0.37	0.83 ± 0.05
		40	$\mathbf{2.32 \pm 0.38}$	1.47 ± 0.42	0.81 ± 0.06
		50	$\mathbf{2.48 \pm 0.45}$	1.59 ± 0.49	0.78 ± 0.08

Bold values indicate the best-performing models (lowest mean RMSE) at each addition level.

Table S8: Average test performance metrics for each decoder model under increasing spike addition (false-positive) levels for Subject 2. Values are reported as means with standard deviations, computed across finger tasks and movement directions.

**Subject**	**Decoder Model**	**Addition Level (%)**	**RMSE** (%MVC)	**MAE** (%MVC)	$\mathbf{R^2}$
S2	Baseline (LR)	0	2.13 ± 0.54	1.36 ± 0.45	0.84 ± 0.08
		1	2.13 ± 0.53	1.36 ± 0.44	0.84 ± 0.08
		2	2.13 ± 0.52	1.36 ± 0.44	0.84 ± 0.07
		3	2.13 ± 0.51	1.36 ± 0.43	0.84 ± 0.07
		4	2.13 ± 0.50	1.36 ± 0.43	0.84 ± 0.07
		5	2.13 ± 0.49	1.36 ± 0.43	0.84 ± 0.07
		10	2.17 ± 0.44	1.40 ± 0.42	0.83 ± 0.06
		15	2.24 ± 0.44	1.45 ± 0.45	0.83 ± 0.06
		20	2.33 ± 0.47	1.52 ± 0.48	0.81 ± 0.07
		30	2.58 ± 0.54	1.69 ± 0.54	0.77 ± 0.09
		40	2.90 ± 0.67	1.91 ± 0.60	0.71 ± 0.14
		50	3.26 ± 0.82	2.14 ± 0.65	0.62 ± 0.20
	SNN LI	0	2.32 ± 0.46	1.64 ± 0.40	0.82 ± 0.06
		1	2.32 ± 0.45	1.64 ± 0.39	0.82 ± 0.06
		2	2.31 ± 0.44	1.64 ± 0.39	0.82 ± 0.06
		3	2.31 ± 0.43	1.63 ± 0.39	0.82 ± 0.06
		4	2.30 ± 0.42	1.63 ± 0.39	0.83 ± 0.06
		5	2.30 ± 0.41	1.63 ± 0.39	0.83 ± 0.06
		10	2.30 ± 0.39	1.63 ± 0.39	0.83 ± 0.06
		15	2.32 ± 0.38	1.64 ± 0.39	0.82 ± 0.06
		20	2.36 ± 0.38	1.67 ± 0.40	0.82 ± 0.06
		30	2.48 ± 0.40	1.75 ± 0.43	0.80 ± 0.07
		40	2.65 ± 0.49	1.86 ± 0.46	0.76 ± 0.09
		50	2.86 ± 0.65	1.99 ± 0.53	0.72 ± 0.13
	SNN LIF	0	$\mathbf{2.08 \pm 0.54}$	1.42 ± 0.49	0.85 ± 0.07
		1	$\mathbf{2.08 \pm 0.54}$	1.42 ± 0.50	0.85 ± 0.07
		2	$\mathbf{2.08 \pm 0.53}$	1.43 ± 0.49	0.86 ± 0.07
		3	$\mathbf{2.08 \pm 0.53}$	1.43 ± 0.50	0.85 ± 0.07
		4	$\mathbf{2.08 \pm 0.53}$	1.44 ± 0.50	0.85 ± 0.07
		5	$\mathbf{2.08 \pm 0.53}$	1.44 ± 0.50	0.85 ± 0.07
		10	$\mathbf{2.11 \pm 0.51}$	1.46 ± 0.49	0.85 ± 0.07
		15	$\mathbf{2.16 \pm 0.51}$	1.49 ± 0.49	0.84 ± 0.07
		20	$\mathbf{2.21 \pm 0.48}$	1.54 ± 0.48	0.84 ± 0.07
		30	$\mathbf{2.33 \pm 0.46}$	1.63 ± 0.48	0.82 ± 0.07
		40	$\mathbf{2.48 \pm 0.46}$	1.73 ± 0.48	0.79 ± 0.08
		50	$\mathbf{2.64 \pm 0.49}$	1.85 ± 0.48	0.77 ± 0.09

Bold values indicate the best-performing models (lowest mean RMSE) at each addition level.

Table S9: Average test performance metrics for each decoder model under increasing cross-motor unit misattribution levels. Values are reported as means with standard deviations, computed across finger tasks and movement directions.

**Subject**	**Decoder Model**	**Misattribution Level (%)**	**RMSE** (%MVC)	**MAE** (%MVC)	$\mathbf{R}^2$
S1	LR	0	$\mathbf{1.73 \pm 0.26}$	1.01 ± 0.18	0.88 ± 0.04
		1	$\mathbf{1.74 \pm 0.26}$	1.01 ± 0.18	0.88 ± 0.04
		3	$\mathbf{1.74 \pm 0.25}$	1.01 ± 0.18	0.88 ± 0.04
		5	1.75 ± 0.24	1.01 ± 0.18	0.88 ± 0.04
		10	1.77 ± 0.25	1.03 ± 0.18	0.88 ± 0.04
		15	1.78 ± 0.26	1.04 ± 0.18	0.88 ± 0.04
		20	1.81 ± 0.25	1.05 ± 0.17	0.87 ± 0.04
		50	1.94 ± 0.29	1.12 ± 0.17	0.86 ± 0.05
	SNN LI	0	1.74 ± 0.32	1.10 ± 0.17	0.89 ± 0.04
		1	$\mathbf{1.74 \pm 0.32}$	1.10 ± 0.17	0.89 ± 0.04
		3	$\mathbf{1.74 \pm 0.32}$	1.10 ± 0.17	0.89 ± 0.04
		5	$\mathbf{1.74 \pm 0.31}$	1.10 ± 0.17	0.89 ± 0.04
		10	$\mathbf{1.75 \pm 0.32}$	1.11 ± 0.18	0.89 ± 0.04
		15	$\mathbf{1.75 \pm 0.33}$	1.11 ± 0.18	0.89 ± 0.04
		20	$\mathbf{1.75 \pm 0.33}$	1.11 ± 0.18	0.89 ± 0.04
		50	$\mathbf{1.79 \pm 0.34}$	1.15 ± 0.19	0.89 ± 0.04
	SNN LIF	0	1.84 ± 0.31	1.13 ± 0.25	0.88 ± 0.05
		1	1.85 ± 0.32	1.13 ± 0.25	0.88 ± 0.05
		3	1.84 ± 0.31	1.14 ± 0.26	0.88 ± 0.04
		5	1.83 ± 0.32	1.13 ± 0.26	0.88 ± 0.04
		10	1.84 ± 0.30	1.14 ± 0.25	0.88 ± 0.04
		15	1.86 ± 0.32	1.16 ± 0.27	0.87 ± 0.04
		20	1.89 ± 0.31	1.18 ± 0.28	0.87 ± 0.04
		50	1.92 ± 0.28	1.22 ± 0.27	0.87 ± 0.04
S2	LR	0	2.13 ± 0.54	1.36 ± 0.45	0.84 ± 0.08
		1	2.13 ± 0.54	1.36 ± 0.45	0.84 ± 0.08
		3	2.14 ± 0.53	1.37 ± 0.45	0.84 ± 0.08
		5	2.15 ± 0.55	1.38 ± 0.46	0.84 ± 0.08
		10	2.17 ± 0.56	1.39 ± 0.47	0.83 ± 0.08
		15	2.21 ± 0.56	1.41 ± 0.47	0.83 ± 0.08
		20	2.22 ± 0.58	1.42 ± 0.49	0.83 ± 0.08
		50	2.38 ± 0.65	1.52 ± 0.53	0.80 ± 0.10
	SNN LI	0	2.32 ± 0.46	1.64 ± 0.40	0.82 ± 0.06
		1	2.32 ± 0.46	1.64 ± 0.39	0.82 ± 0.06
		3	2.32 ± 0.47	1.64 ± 0.39	0.82 ± 0.06
		5	2.32 ± 0.47	1.64 ± 0.39	0.82 ± 0.07
		10	2.32 ± 0.49	1.65 ± 0.40	0.82 ± 0.07
		15	2.33 ± 0.49	1.65 ± 0.39	0.82 ± 0.07
		20	2.33 ± 0.51	1.66 ± 0.39	0.82 ± 0.07
		50	2.37 ± 0.56	1.70 ± 0.41	0.81 ± 0.08
	SNN LIF	0	$\mathbf{2.08 \pm 0.54}$	1.42 ± 0.49	0.85 ± 0.07
		1	$\mathbf{2.08 \pm 0.54}$	1.42 ± 0.50	0.85 ± 0.07
		3	$\mathbf{2.08 \pm 0.55}$	1.43 ± 0.50	0.85 ± 0.07
		5	$\mathbf{2.09 \pm 0.54}$	1.44 ± 0.50	0.85 ± 0.07
		10	$\mathbf{2.10 \pm 0.56}$	1.45 ± 0.51	0.85 ± 0.08
		15	$\mathbf{2.11 \pm 0.55}$	1.46 ± 0.51	0.85 ± 0.08
		20	$\mathbf{2.13 \pm 0.54}$	1.48 ± 0.50	0.85 ± 0.07
		50	$\mathbf{2.22 \pm 0.59}$	1.54 ± 0.55	0.84 ± 0.09

Bold values indicate the best-performing models (lowest mean RMSE) at each misattribution level for each subject.

Table S10: Mean firing activity and estimated power consumption for each finger population on Dynamic Neuromorphic Asynchronous Processor (DYNAP-SE). r_{mu} and r_{pop} denote the mean input motor unit and population firing rates per sample, respectively, while σ_{mu} and σ_{pop} indicate their corresponding standard deviations. N_{MU} represents the number of motor units providing input to each population, and P_{tot} denotes the average total power consumption associated with each population.

Finger	Direction	Chip	Core	r_{mu} (Hz)	σ_{mu} (Hz)	N_{MU}	r_{pop} (Hz)	σ_{pop} (Hz)	P_{tot} (μW)
Thumb	Ext	0	1	8.47	2.56	7	12.32	0.78	0.83
Thumb	Flex	0	0	9.19	1.08	4	12.14	0.30	0.69
Index	Ext	0	3	9.31	3.19	14	13.87	2.39	1.32
Index	Flex	0	2	8.63	3.19	13	12.50	0.94	1.16
Middle	Ext	1	1	12.04	2.68	8	10.39	2.90	0.98
Middle	Flex	1	0	10.23	2.71	18	13.50	0.93	1.63
Ring	Ext	1	3	11.36	3.47	41	12.29	0.48	3.28
Ring	Flex	1	2	8.47	2.49	14	12.24	0.44	1.19
Little	Ext	2	1	8.43	3.50	23	12.30	0.89	1.64
Little	Flex	2	0	6.14	0.94	2	11.23	0.45	0.51
**Total Power (μW)**									**13.24**

Table S11: Motor unit decomposition and encoding statistics at 15% MVC. Discharge rates of motor units and encoded iEMG channels are computed across repetitions and finger tasks for each subject using ALIF neurons with $\theta_{\text{base}} = w_\theta = 0.1\,\text{V}$ and $\tau_{\text{adapt}} = 100\,\text{ms}$.

Subject	Direction	Number of motor units	Number of iEMG channels	Motor units discharge rate (pps)	Firing rate of encoded channels (Hz)
S1	Flexion	121	120	11.90 ± 3.70	9.81 ± 3.97
	Extension	196	120	13.00 ± 4.72	12.43 ± 6.80
S2	Flexion	51	120	10.78 ± 3.05	3.90 ± 2.23
	Extension	93	120	12.66 ± 4.38	6.51 ± 4.51
