# Supplementary material for: Spiking neural network decoders of finger forces from high-density intramuscular microelectrode arrays
Source: Nat Commun. 2026 Jun 24;17:7937. doi: 10.1038/s41467-026-74243-1 (PMC13447840; doi:10.1038/s41467-026-74243-1)
Supplement: Supplementary file 2 [file 41467_2026_74243_MOESM2_ESM.pdf]

nature portfolio

Corresponding author(s): Farah Baracat

Last updated by author(s): Apr 24, 2026

Reporting Summary

Nature Portfolio wishes to improve the reproducibility of the work that we publish. This form provides structure for consistency and transparency in reporting. For further information on Nature Portfolio policies, see our Editorial Policies and the Editorial Policy Checklist.

Statistics

For all statistical analyses, confirm that the following items are present in the figure legend, table legend, main text, or Methods section.

n/a	Confirmed	
☐	☒	The exact sample size (*n*) for each experimental group/condition, given as a discrete number and unit of measurement
☐	☒	A statement on whether measurements were taken from distinct samples or whether the same sample was measured repeatedly
☒	☐	The statistical test(s) used AND whether they are one- or two-sided<br>*Only common tests should be described solely by name; describe more complex techniques in the Methods section.*
☒	☐	A description of all covariates tested
☒	☐	A description of any assumptions or corrections, such as tests of normality and adjustment for multiple comparisons
☐	☒	A full description of the statistical parameters including central tendency (e.g. means) or other basic estimates (e.g. regression coefficient) AND variation (e.g. standard deviation) or associated estimates of uncertainty (e.g. confidence intervals)
☒	☐	For null hypothesis testing, the test statistic (e.g. *F*, *t*, *r*) with confidence intervals, effect sizes, degrees of freedom and *P* value noted<br>*Give P values as exact values whenever suitable.*
☒	☐	For Bayesian analysis, information on the choice of priors and Markov chain Monte Carlo settings
☐	☒	For hierarchical and complex designs, identification of the appropriate level for tests and full reporting of outcomes
☐	☒	Estimates of effect sizes (e.g. Cohen's *d*, Pearson's *r*), indicating how they were calculated

Our web collection on statistics for biologists contains articles on many of the points above.

Software and code

Policy information about availability of computer code

Data collection	Recordings of HD-iEMG was not part of the current study, but can be found in https://arxiv.org/abs/2410.11016v2.
Data analysis	Code used to analyse the data and reproduce the main findings is available at https://github.com/FarahBaracat/snn-fingerforce-decoding. Data analysis and simulations were performed in Python (v3.11.6) using PyTorch (v2.0.1), SnnTorch (v0.7.0), NumPy (v1.26.4), and SciPy (v1.11.3). Visualizations were generated using Matplotlib (v3.8.1).

For manuscripts utilizing custom algorithms or software that are central to the research but not yet described in published literature, software must be made available to editors and reviewers. We strongly encourage code deposition in a community repository (e.g. GitHub). See the Nature Portfolio guidelines for submitting code & software for further information.

Data

Policy information about availability of data

All manuscripts must include a data availability statement. This statement should provide the following information, where applicable:

- Accession codes, unique identifiers, or web links for publicly available datasets
- A description of any restrictions on data availability
- For clinical datasets or third party data, please ensure that the statement adheres to our policy

The motor unit decomposition data that support the findings of this study are publicly available on Figshare (https://doi.org/10.6084/m9.figshare.31378327). The original HD-iEMG recordings are not publicly available due to ethical, privacy considerations, and ongoing research but are available from the corresponding author (FB) upon reasonable request and subject to institutional approval. Source data are provided with this paper.

Research involving human participants, their data, or biological material

Policy information about studies with human participants or human data. See also policy information about sex, gender (identity/presentation), and sexual orientation and race, ethnicity and racism.

Reporting on sex and gender	Both participants were male. Sex was not included as a variable in the analysis due to the limited sample size (n = 2), and no sex- or gender-based comparisons were performed.
Reporting on race, ethnicity, or other socially relevant groupings	Race, ethnicity, and other socially relevant groupings were not recorded or considered in this study, as the focus was on within-subject decoding performance. No analyses were performed based on such variables.
Population characteristics	Participants were healthy adult volunteers. No additional covariate-relevant population characteristics were included in the analysis.
Recruitment	Participants were recruited as part of a previously conducted study involving intramuscular microelectrode recordings. The sample reflects the practical constraints of such invasive recordings and may not be representative of the general population.
Ethics oversight	All procedures were conducted in accordance with the Declaration of Helsinki and approved by the relevant institutional review boards at Imperial College London (ICREC Project ID 19IC5640 for intramuscular recordings; JRCO Project ID 18IC4685 for MRI acquisition). All participants provided informed written consent, including consent for the use of their data in subsequent analyses (see https://arxiv.org/pdf/2410.11016v2). No personally identifiable information is included in this manuscript.

Note that full information on the approval of the study protocol must also be provided in the manuscript.

Field-specific reporting

Please select the one below that is the best fit for your research. If you are not sure, read the appropriate sections before making your selection.

[x] Life sciences   [ ] Behavioural & social sciences   [ ] Ecological, evolutionary & environmental sciences

For a reference copy of the document with all sections, see nature.com/documents/nr-reporting-summary-flat.pdf

Life sciences study design

All studies must disclose on these points even when the disclosure is negative.

Sample size	Sample size was not predetermined using statistical methods. The study used an existing dataset (https://arxiv.org/abs/2410.11016v2) comprising n = 2 participants, each performing five finger tasks across two movement directions (flexion and extension), with two repetitions per condition. The sample size was determined by data availability and the practical constraints of invasive high-density intramuscular recordings.
Data exclusions	No data was excluded from the analysis.
Replication	Experiments were replicated across two independent repetitions per condition (five finger tasks and two movement directions) for each subject. Decoding performance was consistent across repetitions, as reflected by similar RMSE, MAE, and R^2 values and overlapping variability estimates. In addition, SNN models were trained across multiple random initializations (10 seeds), yielding comparable performance across runs. All replication attempts were successful, with no systematic differences observed across repetitions or model initializations.
Randomization	Randomization was not applicable to this study, as no experimental groups or treatment conditions were defined. All participants performed all task conditions, and analyses were conducted within-subject. During model training, input data were randomly shuffled across tasks and repetitions to avoid ordering effects.
Blinding	Blinding was not relevant to this study, as no group allocation or subjective outcome assessment was involved. Analyses were performed independently for each subject using fully automated computational pipelines. Given the small number of participants and the absence of experimental groups, blinding procedures were not applicable.

Reporting for specific materials, systems and methods

We require information from authors about some types of materials, experimental systems and methods used in many studies. Here, indicate whether each material, system or method listed is relevant to your study. If you are not sure if a list item applies to your research, read the appropriate section before selecting a response.

Materials & experimental systems

n/a	Involved in the study
☒	☐ Antibodies
☒	☐ Eukaryotic cell lines
☒	☐ Palaeontology and archaeology
☒	☐ Animals and other organisms
☒	☐ Clinical data
☒	☐ Dual use research of concern
☒	☐ Plants

Methods

n/a	Involved in the study
☒	☐ ChIP-seq
☒	☐ Flow cytometry
☒	☐ MRI-based neuroimaging

Plants

Seed stocks

Report on the source of all seed stocks or other plant material used. If applicable, state the seed stock centre and catalogue number. If plant specimens were collected from the field, describe the collection location, date and sampling procedures.

Novel plant genotypes

Describe the methods by which all novel plant genotypes were produced. This includes those generated by transgenic approaches, gene editing, chemical/radiation-based mutagenesis and hybridization. For transgenic lines, describe the transformation method, the number of independent lines analyzed and the generation upon which experiments were performed. For gene-edited lines, describe the editor used, the endogenous sequence targeted for editing, the targeting guide RNA sequence (if applicable) and how the editor was applied.

Authentication

Describe any authentication procedures for each seed stock used or novel genotype generated. Describe any experiments used to assess the effect of a mutation and, where applicable, how potential secondary effects (e.g. second site T-DNA insertions, mosiacism, off-target gene editing) were examined.
