# Supplementary material for: Spiking neural network decoders of finger forces from high-density intramuscular microelectrode arrays
Source: Nat Commun. 2026 Jun 24;17:7937. doi: 10.1038/s41467-026-74243-1 (PMC13447840; doi:10.1038/s41467-026-74243-1)
Supplement: Supplementary file 3 [file 41467_2026_74243_MOESM3_ESM.pdf]

nature portfolio

Peer Review File

Spiking Neural Network Decoders of Finger Forces from High-Density Intramuscular Microelectrode Arrays

Corresponding Author: Ms Farah Baracat

This file contains all reviewer reports in order by version, followed by all author rebuttals in order by version.

Version 0:

Reviewer comments:

Reviewer #1

(Remarks to the Author)
The paper compares different SNN-based solutions to perform finger force regression from HD-iEMG electrodes during flexion and extension movements. The examined solutions exploit an output layer including 5 LI neurons, whose membrane voltage is trained to reproduce the force variable, applied to the spikes resulting from motor unit decomposition or encoded through an input layer of LIF neurons.

The comparison with a linear regression approach shows promising results, with an efficient event-driven solution, demonstrating higher robustness to noise and interference modeled as missing spikes, while removing the need for the storage of the separation matrices needed for motor unit decomposition.

- the proposed SNN architecture is indeed extremely efficient; however, it could be of interest to analyze a comparison with other SNN topologies exploited in the literature for EMG processing, besides the baseline linear regression solution, highlighting the most relevant advantages; the authors only mention two studies applying SNNs to motor unit spikes in HD-sEMG signals, without commenting the complexity/accuracy of such models, and do not consider other solutions applied to spike-encoded HD-sEMG signals;

- can the authors comment whether the proposed approach would be suitable for HD-sEMG acquisition?

- can the authors clarify why the encoding SNN solution has so many non-trainable vs trainable parameters?

- Minor: The results in Supplementary Table 2 would be more easily interpreted by highlighting the best solution for each omission level (whether it is baseline, or SNN LI, or SNN LIF).

(Remarks on code availability)

Reviewer #2

(Remarks to the Author)
This paper introduces a spike-based decoding framework combining spiking neural networks (SNNs) with motor unit activity extracted from high-density intramuscular microelectrode arrays (HD-iMEAs). The study systematically evaluates different SNN configurations, compares motor unit spike trains with spike-encoded intramuscular EMG (HD-iEMG), and analyzes trade-offs between decoding accuracy, robustness, and memory footprint. The work is novel in exploring SNNs for proportional finger force decoding and demonstrates the potential of neuromorphic processing for low-latency applications. However, there are some weaknesses and concerns:
1. Insufficient emphasis on significance: The introduction overemphasizes methodological details while downplaying the broader importance of the work. A clearer depiction of the research significance, particularly in the context of assistive technologies and neuromorphic computing, would strengthen the framing.

2. The reported performance of encoded HD-iEMG and SNN is not clear: The encoded HD-iEMG input showed a considerable performance drop compared with baseline (e.g., RMSE increased from 1.73 to 2.55 in Subject 1). The paper should explicitly discuss whether this trade-off is acceptable for practical applications and clarify how the memory savings (e.g., avoiding decomposition matrices) compensate for the reduced accuracy. Supplementary Table 4 reports Encoding SNN, but this configuration is not explained in the main text and should be clarified.
3. Unclear clarification of data collection and model outputs: In Section 4.1, it is stated that only one finger was active during each task, while the other fingers remained at rest. However, in Sections 4.3 and 4.4, the decoder models are described as having multiple outputs corresponding to all five fingers. This discrepancy may cause confusion for readers. I suggest that the authors clarify this point explicitly in the manuscript and, if possible, include a schematic figure illustrating the complete pipeline from data acquisition (single-finger activation) to multi-output decoding. Such a figure would make the data flow and model outputs much clearer.
4. Limited dataset and unclear results: Only two subjects were included, with no detailed description of training/testing splits or per-subject data amount. The paper does not clarify whether datasets were collected in one session or across multiple days, which is critical for assessing stability and practical applications. Meanwhile, only two subjects failed to show the generalizability of the proposed methods.
5. Comprehensiveness of Baselines. Currently, the comparisons are limited to a linear regression model. While this demonstrates that SNNs can achieve “comparable” performance, it does not sufficiently showcase their advantages over modern decoding approaches. It is strongly suggested to include comparisons with at least one nonlinear deep learning model (e.g., 1D CNN or small-scale RNN) to highlight the potential benefits of SNNs in latency, power consumption, or event-driven processing under comparable accuracy. In addition, beyond LI and LIF neuron models, a brief discussion or experiment involving more expressive models such as PLIF could be included to explore the performance upper bound.
6. Quantitative Evidence of System-Level Advantages. Although the paper emphasizes the advantages of SNNs in latency and memory efficiency, the arguments remain largely theoretical and lack key quantitative metrics (e.g., inference latency, energy consumption, or simulated FLOPs). It is recommended to supplement this with quantitative analysis of computational complexity and theoretical latency.
7. In-Depth Analysis of Performance Differences. The explanation for the significant performance drop of spike-encoded HD-iEMG in the flexion task is somewhat general, attributing it to “signal amplitude variability and fixed thresholds.” However, it lacks deeper empirical evidence to support this conclusion and point to potential solutions. Ablation or sensitivity analyses are suggested to illustrate how varying thresholds affect event rates and final RMSE. The discussion could also more specifically reference and prospect how adaptive thresholds [40] or heterogeneous coding populations [36] might alleviate this issue.
8. Scope of Robustness Evaluation. The robustness tests currently only simulate one type of decomposition error (missed detections), without considering other common real-world disturbances such as false positives, electrode shifts, or EMG amplitude variations. It is recommended to clearly state the boundaries of the current robustness evaluation in the discussion or limitations section, address the impact of other real-world interference factors, and propose how these scenarios could be systematically validated in the future.

(Remarks on code availability)
N/A

Reviewer #3

(Remarks to the Author)
Summary & Key Results:
This study uses signals from high-density intramuscular EMG (HD-iEMG) and compares three input-to-decoder routes for simultaneous, proportional, and continuous decoding of five-finger forces—(1) MU spikes → SNN, (2) iEMG → LIF event encoding → SNN, and (3) MU binning → linear regression. The work is end-to-end, covering physiology and hardware, data processing, and algorithmic implementation, and it includes random MU dropouts to emulate imperfect online decomposition.

Noteworthy results:
• Event-driven SNN without time binning achieves force-regression errors comparable to the linear baseline while reducing latency and memory footprint.
• iEMG → LIF → SNN attains only a small loss in accuracy without MU decomposition, yielding a lighter and more practical implementation.
• Under random MU dropout, the SNN with an intermediate LIF layer is more robust to missing spikes.

Significance & Context:
Applying event-based representations with SNN decoding to continuous proportional control (a regression task, not a classification task) is relevant for prosthetic control and other device-side uses that require low latency and low memory/power. Compared with prior SNN work that mostly uses surface EMG for classification, this manuscript relies on intramuscular signals and targets continuous regression, which is closer to real end-effector control. A head-to-head comparison of iEMG → LIF → SNN versus MU → SNN in the same framework is also new and informative.

Do the data support the claims?:

For the claim that non-binned, event-based decoding can match traditional approaches while lowering latency and memory, the current data largely support the conclusions. It would be helpful to add evidence that is closer to real-world deployment (see Major points).

Methodology & Reproducibility:
Acquisition and synchronization, task design, model structures, and key training details are described clearly. To further improve reproducibility and transparency, please provide code, parameter settings, and a minimal data subset sufficient to reproduce the main figures.

Major points
1. Limited external validity. Please add at least one of the following:
• Closed-loop online or hardware-in-the-loop demonstration, reporting end-to-end latency, throughput, memory, energy per sample, and communication bandwidth;
• Cross-day/cross-session stability plus few-shot calibration results;
• A small clinical cohort (e.g., amputees) or feasibility for dynamic, multi-level force, or multi-finger cooperative tasks.
2. Comparators are too weak to isolate the SNN advantage. Comparing only to linear regression is not sufficient. Please add lightweight ANN baselines with matched parameter count and latency (e.g., small CNN or GRU) under aligned inputs:
• If using binned inputs, use binning for both ANN and SNN;
• If using event inputs, explain how the ANN receives them.
Report MACs per sample, peak/average memory, and inference time, not just errors.
3. Statistics and uncertainty need strengthening. With few participants and repeated trials, uncertainty can be underestimated. Please:
• Use a hierarchical/mixed-effects model and report effect sizes with 95% CIs;
• Apply multiple-comparison contro where appropriate;
• Clarify train/validation/test splits and safeguards against data leakage across splits (the same time segment should not appear in multiple sets).
4. Event encoding depends on manual thresholds; maintainability is unclear. Please add adaptive-threshold or target event-rate closed-loop tests and provide threshold–event-rate–accuracy curves, demonstrating stability under electrode drift and muscle-state changes.
5. Robustness covers only dropouts; misses false positives and misattribution. Online MU decomposition can introduce false-positive spikes and cross-MU misattribution. Please simulate both and quantify their effects on error and latency, comparing MU → SNN, iEMG → LIF → SNN, and the baseline.

Minor points
1. Writing and style consistency (please perform a single, full-manuscript pass):
• Use “real-time” when adjectival and “in real time” as an adverb; unify “multichannel” (do not mix with “multi-channel”);
• In text use “Fig. X”; in captions use “Figure X”;
2. Consolidate method details and clarify online/offline boundaries.
Provide a single table for LIF threshold strategy (per electrode/participant), target event-rate ranges, and time constants. State explicitly which steps are offline and which are online, with time budgets for each.
3. Data and code sharing.
Supply a minimal reproducible package (subset of data, training/inference scripts, and model weights) with dependencies and versioning to reproduce the main figures.

Reporting Summary:
For transparent evaluation, please ensure the Reporting Summary includes: participant information and inclusion/exclusion criteria; sample-size and power considerations; randomization and blinding; preprocessing; hyperparameter search ranges and surrogate-gradient settings; time-constant and threshold configurations; train/validation/test splits and random seeds; outlier handling and multiple-comparison control.

Closing remark:
This is a complete, engineering-oriented study that applies event-based inputs plus SNNs to continuous proportional decoding with a meaningful set of route comparisons. Adding online/hardware-in-the-loop or cross-session/adaptive evidence, fair lightweight ANN baselines, and stronger statistics and reproducibility materials would substantially increase the strength and reach of the paper.

(Remarks on code availability)
Code and data availability：
Based on the materials provided for review, I did not see a code repository, model weights, or a dataset/minimal data subset. The manuscript states that data and code are available from the corresponding author upon reasonable request, which suggests they were not included directly in the review package.
If these materials are already hosted elsewhere, a link in the manuscript (or in the response to reviewers) would be very helpful.

Reviewer #4

(Remarks to the Author)
This paper proposes SNN decoders to decode motor unit spikes from EMG signals. The SNN decoders can achieve similar performance as the Linear Regression baseline while using a much lower memory footprint. In addition, the authors also show that the two-layer SNN decoder can be more robust to random spike omission compared to the baseline. The proposed work can be used in BCI to perform real-time prediction of finger movements. However, there are several concerns from the reviewer that need to be addressed.

Concern 1: The potentially higher computational cost of SNN has not been examined.

If we consider the binned input for linear regression as B, then each step of linear regression computation will be P=WB, where W is the parameter and P is the output. Now, we consider the input dimension as DB and the output dimension as DP. Then the overall computational cost for linear regression will be DB*DP.

Since SNNs do not bin inputs into a single vector, each spike will be integrated into P with a computational cost of DP. Suppose we have sparsity as S and the window size as WIN, then the overall SNN computational cost will be S*WIN*DB*DP. When S*WIN >> 1 (e.g., low sparsity or multiple spikes per motor unit within the window), the SNN may require substantially more operations than the baseline, even if it reduces the activation memory.

Therefore, the authors need to quantify this trade-off. Report the sparsity, FLOPs or synaptic operations, wall-clock time, and energy cost comparisons.

Concern 2: Unknown overall memory and computational cost reduction on the full pipeline.

The decoder is only the terminal stage of the pipeline. Prior to decoding, raw EMG must be decomposed into motor-unit spike trains, a step the authors note is costly but do not quantify for their experiments. Without end-to-end profiling, it is unclear what fraction of total runtime, FLOPs, energy, and memory is attributable to the decoder versus EMG decomposition. If the decomposition dominates, potential savings from using an SNN decoder may have a negligible impact on overall system efficiency.

Therefore, the authors need to show the overall memory and computational cost reduction on the full pipeline.

Concern 3: Inadequate sample size and insufficient baseline comparisons

Two subjects cannot support the paper's architectural and methodological conclusions. The Mann-Whitney U tests use n=20 samples from repeated measures on the same two individuals, violating independence assumptions. Claims about SNN reliability (lines 20-22) are unsubstantiated.

In addition, linear regression on binned spike counts is a very naïve baseline method from the ML perspective that deliberately discards temporal information. It will be interesting to see a comparison with more advanced temporal processing methods like RNN-based approaches. There is also a question about the capability of the proposed method. Since it can only achieve similar (or worse) performance than linear regression, it may achieve much lower performance than other, more advanced methods in more complex tasks and settings.

Therefore, the authors can recruit additional participants (minimum n=10) or reframe entirely as a proof-of-concept feasibility study with appropriate statistical methods for repeated measures. The authors can include at least one recurrent baseline to justify claims about temporal processing advantages.

Concern 4: Real-time claims not validated.

The manuscript frames around real-time, low-latency processing (Abstract, lines 287-299, Fig. 1c), but all analysis is offline. The "10 ms latency" is a simulation parameter, not measured. Without hardware implementation or benchmarking, the core premise remains speculative. Please check Concern 1 as well.

Therefore, the authors can either implement on neuromorphic hardware with measured latencies or remove real-time advantage claims.

Concern 5: The representative of the spike omission and robustness mechanism is unclear.

The authors performed perturbation experiments to show the network's resilience to errors in Section 2.2. However, the experiment is limited to random spike omission. It is not clear how representative random spike omission is of the real-world errors of EMG signals. It feels like this experiment is specifically designed to show the advantage of event-based spike processing. In addition, LIF's noise robustness (Fig. 4) is attributed to thresholding (lines 186-188), but this configuration also has additional layers and parameters.

Therefore, additional experiments with different modes of noise and error are preferred to support the claim. Additional ablation studies are also needed on LI with matched depth, LIF with matched parameters, and threshold sensitivity analysis.

Concern 6: Hyperparameter justification is lacking.

80ms window: sweep results not shown (lines 403-405)

Time constants (30ms, 80ms): no justification provided (lines 459, 462)

Encoding thresholds: inconsistent between subjects, suggesting poor generalizability

Architecture depth: no exploration reported

Therefore, the authors need a systematic hyperparameter analysis and sensitivity studies.

(Remarks on code availability)

Version 1:

Reviewer comments:

Reviewer #1

(Remarks to the Author)
the authors have addressed my concerns.

(Remarks on code availability)

Reviewer #2

(Remarks to the Author)
All my previous comments have been addressed.

(Remarks on code availability)

Reviewer #3

(Remarks to the Author)
I thank the authors for the careful and substantial revision. The manuscript has been significantly strengthened, and most of my previous concerns have now been addressed in a satisfactory manner.

The revised paper now provides a much more convincing and balanced system-level assessment of spike-based force decoding from HD-iEMG. In particular, the addition of matched-capacity recurrent baselines (vanilla RNN and GRU), the expanded analysis of memory footprint and computational cost across the full pipeline, the replacement of fixed-threshold encoding with an adaptive ALIF-based encoder together with threshold–event-rate–accuracy sensitivity analyses, the broader robustness analysis including false positives and cross–motor-unit misattribution, and the proof-of-concept validation on neuromorphic hardware all materially improve the manuscript.

I also appreciate that the authors have now narrowed the framing of the work appropriately. In its revised form, the paper is best interpreted as a feasibility and architectural study rather than a generalization study or a clinically validated real-time control system. Within that more appropriate scope, I think the main conclusions are now largely supported by the data.

The work is of interest to the field because it moves beyond simple classification settings and examines continuous, proportional decoding under explicit resource constraints, which is directly relevant to embedded and neuromorphic control scenarios. The head-to-head comparison between motor-unit-based and spike-encoded input routes is also informative and useful for future device-side implementations.

I therefore support publication in principle.

(Remarks on code availability)
I reviewed the public code repository and find it to be a valuable and generally usable resource for the community. The repository is well structured and includes a clear README, environment specification, configuration guidance, and named entry-point scripts for the main experimental workflows reported in the manuscript, including the SNN, noisy-input, encoded HD-iEMG, RNN, and regression baselines. This substantially improves the transparency of the study and should facilitate reuse by other researchers working on neuromorphic or resource-constrained EMG decoding.

Reviewer #4

(Remarks to the Author)

I want to thank the authors for the detailed response. This makes me feel that my feedback really helped to improve the paper. My concerns regarding comments 4.4 to 4.6 are all solved, as the authors have added additional experiments and results in the revised paper.

Regarding comments 4.1 and 4.2, the authors now include both the full pipeline and the decoding component computation and memory cost comparisons. The conclusions I draw from the newly added results are:

1. The authors are right that the SNN decoding computation is much less than the LR due to high sparsity in input spikes.

2. My guess in the review comment 4.2 is right that the motor unit decomposition dominates both the memory and computational cost of the full pipeline. Therefore, this makes decreasing the cost of the decoding component kind of meaningless.

Regarding the second point of my conclusions, I have two suggestions for the authors:

1. Line 60-63 on the additional memory cost of LR is not really a good motivation anymore, since the motor unit decomposition dominates the memory cost if they are in the same system. In Figure 1, the authors show the possibility of having two systems; maybe the author needs to make it clearer why two systems are better.

2. I agree with the authors that for a real-time low-cost system, a solution not using motor unit decomposition is preferred. However, the current performance of the SNN without motor units is still far from that of the one with them. It will be interesting to see what results LR and RNN will get for the same input data without motor unit decomposition. Will SNN do better both in cost and accuracy?

For comment 4.3, my concern about the statistical test is resolved. Although I understand that the point of having more subjects can be hard, I still think it can help to show the generalizability of the results.

(Remarks on code availability)

Open Access This Peer Review File is licensed under a Creative Commons Attribution 4.0 International License, which permits use, sharing, adaptation, distribution and reproduction in any medium or format, as long as you give appropriate credit to the original author(s) and the source, provide a link to the Creative Commons license, and indicate if changes were made.
In cases where reviewers are anonymous, credit should be given to 'Anonymous Referee' and the source.
The images or other third party material in this Peer Review File are included in the article's Creative Commons license, unless indicated otherwise in a credit line to the material. If material is not included in the article's Creative Commons license and your intended use is not permitted by statutory regulation or exceeds the permitted use, you will need to obtain permission directly from the copyright holder.
To view a copy of this license, visit https://creativecommons.org/licenses/by/4.0/

Response to reviewers for the manuscript “Spiking Neural Network Decoders of Finger Forces from High-Density Intramuscular Microelectrode Arrays”

We thank the Editor and the Reviewers for their careful reading of the manuscript and for the detailed, constructive comments. We have substantially revised the paper in response to these suggestions. The revised manuscript includes new experimental results, additional baseline comparisons, quantitative system-level analyses, and clarifications aimed at strengthening both the technical rigor and the broader significance of the work.

In particular, we have (i) expanded the set of baseline models by adding lightweight recurrent neural networks (vanilla RNN and GRU) with matched parameter counts; (ii) provided a detailed quantitative analysis of memory footprint, computational cost, and effective operation counts across the full decoding pipeline; (iii) extended the robustness evaluation to cover multiple classes of decomposition errors, including false negatives, false positives, and cross–motor-unit misattribution; (iv) added sensitivity analyses for the spike-encoding stage to explicitly relate encoding thresholds, firing rates, and decoding accuracy; and (v) clarified the scope and limitations of the study with respect to dataset size, session variability, and applicability to HD-sEMG recordings. Importantly, to move beyond purely offline validation, we have added a neuromorphic hardware demonstration using the DYNAP-SE mixed-signal processor, reporting measured inference latency and power consumption. Throughout the manuscript, we have revised the Introduction and Discussion to better emphasize the relevance of the proposed spike-based decoding framework for low-latency, resource-constrained assistive technologies, while carefully avoiding overgeneralization beyond the present experimental setting.

Below, we respond to each reviewer comment point by point. In the manuscript, new text and new revisions that replaced old text are marked in blue, while parts that were deleted are shown in ~~red~~.

Replies to reviewer 1

(Remarks to the author)
The paper compares different SNN-based solutions to perform finger force regression from HD-iEMG electrodes during flexion and extension movements. The examined solutions exploit an output layer including 5 LI neurons, whose membrane voltage is trained to reproduce the force variable, applied to the spikes resulting from motor unit decomposition or encoded through an input layer of LIF neurons.
The comparison with a linear regression approach shows promising results, with an efficient event-driven solution, demonstrating higher robustness to noise and interference modeled as missing spikes, while removing the need for the storage of the separation matrices needed for motor unit decomposition.

Comment 1.1

- The proposed SNN architecture is indeed extremely efficient; however, it could be of interest to analyze a comparison with other SNN topologies exploited in the literature for EMG processing, besides the baseline linear regression solution, highlighting the most relevant advantages; the authors only mention two studies applying SNNs to motor unit spikes in HD-sEMG signals, without commenting the complexity/accuracy of such models, and do not consider other solutions applied to spike-encoded HD-sEMG signals;

Reply: We thank the reviewer for this comment, and would like to take this opportunity to clarify a key point. The intention of this work is not to propose a new Spiking Neural Network (SNN) topology per se, but rather to systematically evaluate how shallow, event-driven SNN architectures perform for continuous, multi-output force regression, while explicitly quantifying footprint

and computational cost. We have extended the Introduction to more clearly place our approach in context to prior SNN-based electromyography (EMG) processing studies (see lines 96–109).

More specifically, existing SNN studies in the EMG domain predominantly focus on gesture classification from low-density or high-density surface EMG. These approaches typically rely on spike-encoded global EMG features (e.g., delta modulation, up/down event streams) and employ multi-layer or hybrid SNN architectures, sometimes combined with external readout classifiers, and evaluated on benchmark datasets such as NinaPro. While effective for discrete gesture recognition, these works do not address proportional and simultaneous force regression from decomposed motor unit activity, nor do they explicitly quantify memory and computational trade-offs under resource-constrained conditions.

In contrast, our study focuses on (i) physiologically grounded motor unit spike trains obtained from high-density intramuscular recordings, (ii) continuous multi-output force decoding, and (iii) a systematic analysis of accuracy, memory footprint, and computational cost across decoding pipelines (the analyses in point iii have been added in this revision). Within this scope, and given the novelty of the dataset, we deliberately evaluated shallow SNN architectures in comparison to conventional baselines to isolate architectural trade-offs and assess their suitability for neuromorphic implementation. In this revised manuscript, we have further added the demonstration of the implementation of the proposed decoder on a mixed-signal, general-purpose neuromorphic platform, showing how the measured decoding performance translates under real hardware constraints.

Comment 1.2

- can the authors comment whether the proposed approach would be suitable for HD-sEMG acquisition?

Reply: We thank the reviewer for this interesting point. We have clarified in the Discussion (see lines 539–543) the relation of the proposed approach to high-density surface electromyography (HD-sEMG) recordings. While the experiments presented in this study focus on high-density intramuscular electromyography (HD-iEMG) to ensure high-quality motor unit decomposition, the proposed pipelines and the architectural trade-offs analyzed in this work remain applicable to HD-sEMG setups. The primary differences are expected to lie in the total number of available EMG channels and in the number and quality of the decomposed motor units, which may influence absolute decoding performance but do not alter the relative comparisons or conclusions regarding computational cost, memory footprint, and event-driven processing.

Comment 1.3

- can the authors clarify why the encoding SNN solution has so many non-trainable vs trainable parameters?

Reply: We thank the reviewer and apologize for the lack of clarity in the original manuscript. The relatively large number of parameters in the encoding SNN arises from the use of HD-iEMG signals as direct inputs.
More specifically, the encoding layer maps each HD-iEMG channel to a dedicated spiking neuron, resulting in a one-to-one functional connectivity. However, the footprint analysis accounts for the full weight matrices that needs to be stored in memory, including zero-valued entries. As a consequence, the encoding stage contributes a parameter matrix of size $N_{\text{EMG}} \times N_{\text{EMG}}$, even though only the diagonal elements correspond to active connections.
Importantly, the weights of this encoding projection are fixed and not learned. The majority of trainable parameters are instead associated with the subsequent fully connected layer of leaky neurons, which projects from the encoding layer to the output space (dimension $N_{\text{EMG}} \times N_{\text{out}} = 120 \times 5$). This explains why the encoding SNN exhibits a larger proportion of non-trainable parameters compared to other models.
We have clarified in the main text (see lines 943–951) that the reported parameter counts include both zero and non-zero weight matrices. In addition, we have added a detailed description of the parameter breakdown in Supplementary Material A. Furthermore, we now explicitly report a complementary metric in Supplementary Table 3, namely connection sparsity, which accounts for the presence of pruned weights and provides an intuitive measure of the proportion of inactive connections.

Comment 1.4

- Minor: The results in Supplementary Table 2 would be more easily interpreted by highlighting the best solution for each omission level (whether it is baseline, or SNN LI, or SNN LIF).

Reply: We thank the reviewer for this suggestion. We now highlight the best-performing model in Supplementary Table 7 for each omission level by typesetting in bold the minimum RMSE value across the three decoders (baseline, SNN LI and SNN LIF).

Replies to reviewer 2

(Remarks to the author)
This paper introduces a spike-based decoding framework combining spiking neural networks (SNNs) with motor unit activity extracted from high-density intramuscular microelectrode arrays (HD-iMEAs). The study systematically evaluates different SNN configurations, compares motor unit spike trains with spike-encoded intramuscular EMG (HD-iEMG), and analyzes trade-offs between decoding accuracy, robustness, and memory footprint. The work is novel in exploring SNNs for proportional finger force decoding and demonstrates the potential of neuromorphic processing for low-latency applications. However, there are some weaknesses and concerns:

Comment 2.1

Insufficient emphasis on significance: The introduction overemphasizes methodological details while downplaying the broader importance of the work. A clearer depiction of the research significance, particularly in the context of assistive technologies and neuromorphic computing, would strengthen the framing.

Reply: We thank the reviewer for this comment. We have revised the Introduction to more clearly frame the study in the context of assistive technologies.

Specifically, we now explicitly articulate the practical challenge of achieving proportional and simultaneous finger-level control in assistive systems, emphasizing that decoding accuracy alone is insufficient. We highlight the additional constraints on latency, power consumption, memory footprint, and data bandwidth that arise in wearable and implantable systems (see lines 39–44).

We then motivate spike-based, event-driven processing as a system-level response to these constraints, rather than as a purely methodological alternative (see lines 74–95). Finally, we clarify how the comparative analysis presented in this study informs the selection of decoding pipelines under real-world hardware constraints, explicitly tying architectural trade-offs to the needs of assistive systems (see lines 141–147).

Comment 2.2

The reported performance of encoded HD-iEMG and SNN is not clear: The encoded HD-iEMG input showed a considerable performance drop compared with baseline (e.g., RMSE increased from 1.73 to 2.55 in Subject 1). The paper should explicitly discuss whether this trade-off is acceptable for practical applications and clarify how the memory savings (e.g., avoiding decomposition matrices) compensate for the reduced accuracy. Supplementary Table 4 reports Encoding SNN, but this configuration is not explained in the main text and should be clarified.

Reply: We thank the reviewer for raising this important point. In the revised manuscript, we now explicitly quantify and discuss the trade-off between decoding accuracy, memory footprint, and computational cost for both the motor unit–based and spike-encoded HD-iEMG pipelines (see Fig. 3).
More specifically, we now comment explicitly on the observed increase in root mean squared error (RMSE) (approximately 0.6–1 %maximal voluntary contraction (MVC), depending on the subject and condition) when using the spike-encoded HD-iEMG representation. We emphasize that whether this drop in accuracy is acceptable depends on the intended application and its tolerance to force estimation errors. For tasks requiring very fine force modulation, even small deviations may be functionally relevant (see lines 440–474). However, in many assistive control scenarios, decoding errors within a few percent of MVC remain within a practically usable range, particularly when considered alongside the substantial reduction in memory footprint and computational cost achieved by bypassing motor unit decomposition.

We further clarify that the primary savings arise from eliminating the need to store subject-specific separation matrices and from avoiding the computational overhead of motor unit decomposition, which dominates inference-time cost in the motor unit–based pipeline. By contrast, the spike-encoding approach maintains a fixed and lightweight memory allocation and introduces only a minor computational overhead, resulting in markedly lower overall processing requirements.

In addition, we have replaced the label "Encoding SNN" with "SNN (ALIF)" in Supplementary Tables 3 and 6 to clarify that this configuration corresponds to the pipeline including the spike-encoding stage implemented with ALIF neurons. We hope this modification removes any ambiguity.

Comment 2.3

Unclear clarification of data collection and model outputs: In Section 4.1, it is stated that only one finger was active during each task, while the other fingers remained at rest. However, in Sections 4.3 and 4.4, the decoder models are described as having multiple outputs corresponding to all five fingers. This discrepancy may cause confusion for readers. I suggest that the authors clarify this point explicitly in the manuscript and, if possible, include a schematic figure illustrating the complete pipeline from data acquisition (single-finger activation) to multi-output decoding. Such a figure would make the data flow and model outputs much clearer.

Reply: We thank the reviewer for raising this point and apologize for the confusion. We have revised the manuscript (see Sections 4.1 and 4.3, lines 698–705 and lines 742–747) to make this point clear.

Indeed, the experimental protocol involves the intentional activation of a single finger at a time. However, the recorded force traces show that the remaining fingers are not strictly inactive. As illustrated in Fig. 1c, non-task fingers still exhibit some involuntary forces, which can reach approximately 2–5 %MVC. These forces are contribute to the recorded EMG signals and therefore to the activity of the decomposed motor units.
For this reason, the decoding problem is formulated as a multi-output regression task that estimates the forces of all five fingers simultaneously, even during nominally single-finger trials (Section 4.3). One one side, this formulation allows the decoder to faithfully capture the coupling between neural activity and measured forces, without artificially constraining non-task finger outputs to zero. On the other, this modeling choice naturally supports scalability to more complex control scenarios involving simultaneous multi-finger activations, without requiring changes to the decoder architecture. We have modified Fig. 1, to include a schematic illustrating the complete pipeline (Fig. 1a).

Comment 2.4

Limited dataset and unclear results: Only two subjects were included, with no detailed description of training/testing splits or per-subject data amount. The paper does not clarify whether datasets were collected in one session or across multiple days, which is critical for assessing stability and practical applications. Meanwhile, only two subjects failed to show the generalizability of the proposed methods.

Reply: We thank the reviewer for this comment. We acknowledge that the present study includes only two subjects and fully agree that with a limited number of participants, we can not make population-level claims. We have revised the manuscript to make this point explicit (see Discussion lines 670–672).

We would like to clarify that the present work is intended as an initial step toward systematically understanding how best to interface spiking neural network backends with motor units decomposed from HD-iEMG recordings. To the best of our knowledge, this is the first study employing multiple high-density intramuscular microelectrode arrays in humans to proportionally decode individual finger forces from motor unit activity. The experimental protocol required precise targeting of forearm muscles and careful validation of decomposition quality, which made data collection technically demanding, particularly in the early stages. For this reason, the study was limited to two participants, allowing us to focus on carefully quantifying and comparing different decoding strategies.

Our previous work demonstrated finger task classification from decomposed motor units [1]. Here, we extend that line of research by shifting the focus toward proportional force decoding and, importantly, toward a computational and deployment-oriented perspective, examining how motor unit extraction can be combined with spike-based decoding architectures, and what are resource-related considerations (e.g. footprint, computational cost) arise with from this integration.

We nevertheless hope that, by clearly articulating the limitations of the dataset and cohort size (see lines 667–670), the present study is appropriately interpreted as a feasibility and architectural investigation, intended to inform future work on larger cohorts, multi-session datasets, and more complex control scenarios.

Comment 2.5

Comprehensiveness of Baselines. Currently, the comparisons are limited to a linear regression model. While this demonstrates that SNNs can achieve "comparable" performance, it does not sufficiently showcase their advantages over modern decoding approaches. It is strongly suggested to include comparisons with at least one nonlinear deep learning model (e.g., 1D CNN or small-scale RNN) to highlight the potential benefits of SNNs in latency, power consumption, or event-driven processing under comparable accuracy. In addition, beyond LI and LIF neuron models, a brief discussion or experiment involving more expressive models such as PLIF could be included to explore the performance upper bound.

Reply: We thank the reviewer for this constructive suggestion. As summarized above, we have substantially extended the analyses to address this point. Specifically, we now include (i) lightweight nonlinear baselines (vanilla Recurrent Neural Network (RNN) and Gated Recurrent Unit (GRU)) and compare all decoding pipelines not only in terms of accuracy but also memory footprint and computational cost; (ii) an adaptive leaky integrate-and-fire (ALIF)-based configuration within the encoding pipeline; and (iii) a demonstration of the proposed SNN approach on mixed-signal neuromorphic hardware. The manuscript has been revised accordingly.

We would first like to clarify our initial decision not to include additional deep learning baselines. This choice was motivated by two main considerations. First, the relatively small size of the dataset, as this work represents an initial systematic study of HD-iEMG-based proportional decoding of finger forces in two subjects, raised concerns about overfitting when using more complex models. Second, our original experimental design aimed to maintain comparability with existing literature that relies on decomposed motor units and linear regression for isometric force decoding [2, 3].

Nonetheless, in the revised manuscript, we now include two lightweight nonlinear baselines, namely a vanilla RNN and a GRU. Both models use a restricted hidden dimension ($H = 5$), resulting in a number of trainable parameters comparable to that of the proposed SNN architectures (see lines 208–217). This choice was made to ensure a fair comparison under matched model capacity and to mitigate overfitting given the limited dataset size. In addition to decoding accuracy, we now report memory footprint and computational costs (see Methods Sections 4.8 and 4.9).

Under these constrained settings, the RNNs and GRU models did not yield substantial performance improvements over the linear regression (LR) baseline or the SNN-based decoders, with average RMSE differences remaining within approximately 0.2–0.6 %MVC across subjects (see Table 1 and lines 218–243). As discussed in the Results section, we acknowledge two important caveats. First, the limited amount of training data may restrict the potential of recurrent architectures. Second, to match parameter counts, the RNNs were limited to a single recurrent layer with only five hidden units, which may not be sufficient to capture more complex nonlinear temporal structure, even if present. Therefore, the absence of clear gains should be interpreted in the context of matched-capacity and limited-data conditions, rather than as a general limitation of recurrent models.

Regarding computational resources, in the main text we now restrict the comparison to the average number of floating-point operations (FLOPs)/synaptic operations (SynOps) operations required per input sample (Fig. 3), accounting for all stages of each decoding pipeline. As shown in the Results (see lines 244–272), the recurrent baselines incur substantially higher operation counts than both the linear decoder and the SNNs, even under a conservative dense-computation assumption. Taken together, these results motivated our decision to proceed with the linear baseline as the primary comparator for the SNNs in subsequent experiments.

In the revision, we further implemented the leaky integrate-and-fire (LIF)-based decoder on the DYNAP-SE mixed-signal neuromorphic processor and report direct measurements of inference latency and on-chip power consumption (Methods Section 4.11, see lines 483–527). On this hardware platform, inference was achieved with approximately 35 ms latency and an average measured power consumption of approximately 13 μW.
We would like to emphasize that these measurements pertain specifically to this general-purpose research neuromorphic platform, which operates under its own architectural constraints and design trade-offs. They should therefore not be interpreted as a direct translation from idealized software simulation to optimized hardware performance. Rather, they serve to illustrate the feasibility of deploying the proposed spike-based decoder on event-driven neuromorphic hardware, and to provide an indicative estimate of latency and power under realistic hardware conditions.

Finally, regarding model expressiveness, we agree that more complex neuron models could potentially increase the performance upper bound. In this direction, the revised manuscript already includes an ALIF-based encoding architecture, which introduces adaptive firing thresholds to better accommodate amplitude variability in spike-encoded HD-iEMG signals (see Section 2.3, lines 385–409, and lines 615–623).

Comment 2.6

Quantitative Evidence of System-Level Advantages. Although the paper emphasizes the advantages of SNNs in latency and memory efficiency, the arguments remain largely theoretical and lack key quantitative metrics (e.g., inference latency, energy consumption, or simulated FLOPs). It is recommended to supplement this with quantitative analysis of computational complexity and theoretical latency.

Reply: We thank the reviewer for this valuable comment and fully agree that a quantitative characterization of system-level performance is important to support the reported advantages of SNNs. We have extended the manuscript to primarily include a detailed quantitative analysis of computational costs. Although we also report the average and peak runtime memory usage and

wall-clock inference time per sample (Supplementary Tables 4,5,6), we focus in the main text on operation counts; this metric is independent of any specific hardware or simulation environment and would therefore allow for a fair comparison across the pipelines.

In particular, we estimate the average number of FLOPs and SynOps for the baseline decoders (OLS LR and RNNs) and for the SNN pipelines. These estimates are further broken down by processing stage: for the baseline models, we separately report the multiply–accumulate (MAC) operations associated with input preprocessing and decoding, while for the SNNs we report both dense and effective SynOps to account for differences in hardware realization and activity-dependent sparsity (see Fig. 3, and lines 244–272).

To provide a realistic assessment of latency and energy efficiency under event-driven execution, we additionally mapped the LIF-based decoder onto a general-purpose, mixed-signal, asynchronous neuromorphic processor. On the DYNAP-SE platform, we report direct measurements of inference latency and energy consumption (see Section 4.11, see lines 483–527 and Fig. 11). Specifically, we measure an average inference latency of 35.96 ± 99.81 ms at 13 µW per sample.

Comment 2.7

In-Depth Analysis of Performance Differences. The explanation for the significant performance drop of spike-encoded HD-iEMG in the flexion task is somewhat general, attributing it to "signal amplitude variability and fixed thresholds." However, it lacks deeper empirical evidence to support this conclusion and point to potential solutions. Ablation or sensitivity analyses are suggested to illustrate how varying thresholds affect event rates and final RMSE. The discussion could also more specifically reference and prospect how adaptive thresholds [40] or heterogeneous coding populations [36] might alleviate this issue.

Reply: We thank the reviewer for this comment. In this revision, we replaced the manually tuned encoding neurons with an adaptive threshold mechanism based on ALIF neurons (see lines 385–409). This adaptation mechanism explicitly accounts for the signal amplitude variability observed across electrodes, finger tasks, and subjects (Supplementary Fig. 10). By dynamically adjusting the firing threshold based on recent activity, it provides an intrinsic normalization of firing rates across channels and eliminates the need for electrode-specific manual calibration.

To further examine how encoding parameters influence downstream decoding, we conducted a sensitivity analysis of the baseline threshold θ_{base} and the adaptation scale w_θ (Fig. 9). The analysis reveals a clear relationship between ALIF parameters, the resulting firing activity, and final decoding accuracy. When the encoding layer generates event rates that deviate from those of physiological motor units (10-12 hertz), decoding performance deteriorates (see lines 422–429). This behavior is expected, as the downstream network parameters were kept unchanged to enable a controlled comparison between input modalities. In contrast, an intermediate parameter regime, for both subjects, yields firing statistics more closely aligned with motor unit activity and results in consistently lower errors for both subjects, despite differences in anatomical electrode placement. These results directly address the limitations of fixed-threshold encoding highlighted in the original manuscript.

Even so, a residual accuracy gap remains between the motor unit–based and spike-encoded pipelines. We now explicitly quantify and contextualize this difference in the main text and discuss it within a broader trade-off between decoding precision, memory footprint, and computational cost (see Fig.3, lines 440–474). The observed increase in error for spike-encoded HD-iEMG remains on the order of approximately 1 %MVC.

Importantly, although the introduction of adaptive thresholding improves robustness across subjects and removes the need for manual calibration, it does not fully eliminate the performance gap anticipated in the original manuscript. This indicates that the remaining difference cannot be explained solely by suboptimal threshold selection, but more likely reflects the representational shift that occurs when moving from physiologically grounded motor unit discharge timings to a more global HD-iEMG-based representation, even when expressed in the event domain. As outlined in the Introduction, motor unit activity provides a direct and temporally precise description of the descending motor drive, whereas global EMG signals inherently aggregate activity across multiple sources.
Accordingly, we position event-based encoding as a viable lower-complexity alternative when decomposition is impractical or infeasible. Adaptive thresholding stabilizes this representation across conditions, and future work may investigate heterogeneous encoding populations to further enhance sensitivity to input variability while preserving the computational advantages of the approach (see lines 629–641).

Comment 2.8

Scope of Robustness Evaluation. The robustness tests currently only simulate one type of decomposition error (missed detections), without considering other common real-world disturbances such as false positives, electrode shifts, or EMG amplitude variations. It

is recommended to clearly state the boundaries of the current robustness evaluation in the discussion or limitations section, address the impact of other real-world interference factors, and propose how these scenarios could be systematically validated in the future.

Reply: We thank the reviewer for this comment. We have now extended our evaluation to include other types of decomposition errors, namely false-positives (i.e. spike additions) and cross-motor unit misattribution errors. Their impact on decoding performance is now reported in the main text (lines 326–348, Figs. 6 and 7), with detailed quantitative results provided in Supplementary Tables 8, 9, and 10.
Overall, the results show that all decoding approaches are less sensitive to false positives than to false negatives. This behavior is linked to the trapezoidal force profiles used in our experiments: random spike additions mainly affect the transient ramp phases, leading to short-lived force overestimation, whereas the longer hold phase dominates the overall prediction. As a result, errors induced by false positives are generally less detrimental than those caused by missed detections. In contrast, cross–motor unit misattributions have a negligible impact, as they largely preserve the global spike timing structure and therefore minimally disrupt the decoded force trajectory.

Finally, we have clarified the scope and boundaries of the robustness analysis in the revised Discussion (see lines 594–604). Specifically, we now state explicitly that the robustness tests were confined to decomposition-level perturbations introduced within the same recording session and therefore reflect relatively mild instabilities. We further acknowledge that larger signal-level non-stationarities—such as those arising from electrode shifts, impedance changes, or cross-session variability—were not directly evaluated in the present study. These aspects are now discussed as important directions for future work, where longitudinal recordings could provide a more comprehensive assessment of robustness.

Replies to reviewer 3

(Remarks to the author)
Summary & Key Results:
This study uses signals from high-density intramuscular EMG (HD-iEMG) and compares three input-to-decoder routes for simultaneous, proportional, and continuous decoding of five-finger forces—(1) MU spikes → SNN, (2) iEMG → LIF event encoding → SNN, and (3) MU binning → linear regression. The work is end-to-end, covering physiology and hardware, data processing, and algorithmic implementation, and it includes random MU dropouts to emulate imperfect online decomposition.

Noteworthy results:

- Event-driven SNN without time binning achieves force-regression errors comparable to the linear baseline while reducing latency and memory footprint.
- iEMG → LIF → SNN attains only a small loss in accuracy without MU decomposition, yielding a lighter and more practical implementation.
- Under random MU dropout, the SNN with an intermediate LIF layer is more robust to missing spikes.

Significance & Context:
Applying event-based representations with SNN decoding to continuous proportional control (a regression task, not a classification task) is relevant for prosthetic control and other device-side uses that require low latency and low memory/power. Compared with prior SNN work that mostly uses surface EMG for classification, this manuscript relies on intramuscular signals and targets continuous regression, which is closer to real end-effector control. A head-to-head comparison of iEMG → LIF → SNN versus MU → SNN in the same framework is also new and informative.

Do the data support the claims?:
For the claim that non-binned, event-based decoding can match traditional approaches while lowering latency and memory, the current data largely support the conclusions. It would be helpful to add evidence that is closer to real-world deployment (see Major points).

Methodology & Reproducibility:
Acquisition and synchronization, task design, model structures, and key training details are described clearly. To further improve reproducibility and transparency, please provide code, parameter settings, and a minimal data subset sufficient to reproduce the main figures.

Reply: We thank the reviewer for raising this point. We now provide the links to the data and code (lines 1074–1078, lines 1079–1082). The decomposed motor unit datasets supporting the main findings have been uploaded on Figshare. The Figshare repository is currently set to private to preserve anonymity during peer review; however, access has been provided to the reviewers. For completeness, the original HD-iEMG recordings have also been made available to the reviewers during the evaluation process. Upon publication, the decomposed motor unit datasets will be made publicly accessible on Figshare, whereas the raw HD-iEMG recordings will remain available upon reasonable request due to ethical and privacy considerations.

Comment 3.1

Major points
Limited external validity. Please add at least one of the following:

- *Closed-loop online or hardware-in-the-loop demonstration, reporting end-to-end latency, throughput, memory, energy per sample, and communication bandwidth;*
- *Cross-day/cross-session stability plus few-shot calibration results;*
- *A small clinical cohort (e.g., amputees) or feasibility for dynamic, multi-level force, or multi-finger cooperative tasks.*

Reply: We thank the reviewer for raising this important point. We would like to first clarify that we position the present study as a first step toward clinical deployment. This work represents an initial attempt to understand and systematically identify effective strategies for interfacing with motor units decomposed from HD-iEMG recordings.
It is important to highlight that, to the best of our knowledge, this is the first study employing multiple high-density intramuscular microelectrode arrays in human participants to decode fine finger forces in a regression framework based on motor unit activity. The experimental protocol required very precise targeting of the muscles involved in finger tasks and was technically challenging, particularly in the initial stages. For these reasons, the study was limited to two participants, allowing us to first systematically quantify and compare the advantages of different decoding backends. Our previous work has demonstrated the classification of finger tasks from motor units [1]. Here, we complement this line of research by introducing a computational and deployment-oriented perspective, exploring the combination of motor unit extraction with spiking neural network backends.

With respect to the reviewer's suggestions, we have addressed the first point by adding a hardware-based decoding demonstration. In particular, we now implement the proposed SNN decoder on a mixed-signal asynchronous neuromorphic processor (DYNAP-SE), developed in our group, and report quantitative measurements of accuracy, inference latency and power consumption. These results provide an initial hardware-in-the-loop validation of LIF-based proposed decoding pipeline and demonstrate how the simulation-based findings translate to a physical, event-driven computing substrate (see Section 2.4, and Methods Section 4.11), taking into consideration the possible chip constraints. We argue that the measured latency of 35.96 ± 99.81 ms and 13 μW power consumption directly support the suitability of the approach for low-latency, device-side deployment. Finally, we explicitly outline how the proposed framework could be extended in future work to address the remaining aspects of external validity highlighted by the reviewer. These include closed-loop and few-shot recalibration experiments, evaluation on dynamic and more complex finger tasks (see lines 594–604, lines 673–678, and lines 662–666).

Comment 3.2

Comparators are too weak to isolate the SNN advantage. Comparing only to linear regression is not sufficient. Please add lightweight ANN baselines with matched parameter count and latency (e.g., small CNN or GRU) under aligned inputs:

- *If using binned inputs, use binning for both ANN and SNN;*
- *If using event inputs, explain how the ANN receives them. Report MACs per sample, peak/average memory, and inference time, not just errors.*

Reply: We thank the reviewer for this suggestion. We have now included two additional lightweight baselines in the main text, namely a vanilla RNN and a GRU, with hidden size restricted to $H = 5$ in order to closely match the parameter count of the proposed SNNs models (lines 208–217, and lines 218–243).
To ensure aligned inputs across models, the RNNs operate on smoothed motor unit spike trains obtained by convolving the original spike sequences with an exponential kernel whose time constant matches the synaptic time constant used in the SNNs (i.e. 10 ms). This preprocessing yields continuous-valued discharge patterns that closely mirror the synaptic integration stage in the SNN, where incoming spikes are filtered before being integrated by the neurons (lines 760–769).

Under matched-capacity and input-aligned conditions, the RNN- and GRU-based pipelines did not yield substantial accuracy improvements over the SNN or linear regression baselines, with differences in RMSE remaining within approximately 0.2–0.6 %MVC across subjects. As discussed in the manuscript, this result should be interpreted in context. The available dataset is limited, which may restrict the ability of recurrent models to leverage more complex temporal structure. In addition, to ensure a fair comparison, the recurrent architectures were constrained to a single layer with five hidden units, limiting their overall capacity. Under these controlled conditions, all models achieved similar decoding accuracy.

In addition, we now report for all models the average number of FLOPs/SynOp operations per input sample, together with the model size (parameter memory). We also measured wall-clock inference time and runtime memory usage (average and peak), which are reported in Supplementary Tables 4, 6, and 5. However, in the main text we focus on the trade-offs between accuracy, model footprint, and operation count, as runtime and memory measurements strongly depend on the specific hardware, operating system, and software framework. The reported timing and memory values were obtained on a standard computing platform (*Apple M1, 8-core CPU*) and reflect execution within our software implementation.

Finally, we include energy estimates for the proposed SNN when deployed directly on neuromorphic hardware DYNAP-SE (Section 4.11, Fig. 11). These measurements provide a conservative lower-bound estimate of the achievable efficiency when the pipeline is implemented on a general-purpose neuromorphic research platform.

Comment 3.3

Statistics and uncertainty need strengthening. With few participants and repeated trials, uncertainty can be underestimated. Please:

- *Use a hierarchical/mixed-effects model and report effect sizes with 95% CIs;*
- *Apply multiple-comparison control where appropriate;*
- *Clarify train/validation/test splits and safeguards against data leakage across splits (the same time segment should not appear in multiple sets).*

Reply: We appreciate the reviewer for raising this point and agree that, with only two subjects, the use of Mann–Whitney tests, or inferential statistical tests more generally, can lead to over-generalization. For this reason, we revised the analysis to focus on reporting within-subject effect sizes together with confidence intervals, as suggested (see lines 199–207, and lines 218–243). We have also clarified in the Methods the data split strategy (see lines 871–873).

In this setting, fitting a hierarchical or mixed-effects model would also not be well supported, as the small number of subjects does not allow for reliable estimation of subject-level variability and could give a misleading sense of population-level inference. Specifically, effect sizes were computed as the mean of paired decoding-error (RMSE) differences across all experimental conditions (finger tasks × movement directions × repetitions) within each subject. Uncertainty was quantified using bootstrap resampling at the repetition level, capturing variability across repeated measurements (see the revised Methods Section 4.10).

Finally, we explicitly acknowledge in the revised Discussion that the limited cohort size does not support strong population-level statistical claims. Accordingly, we frame the statistical analysis as highlighting consistent within-subject trends and comparative architectural trade-offs, rather than generalization across individuals (see lines 670–672).

Comment 3.4

Event encoding depends on manual thresholds; maintainability is unclear. Please add adaptive-threshold or target event-rate closed-loop tests and provide threshold–event-rate–accuracy curves, demonstrating stability under electrode drift and muscle-state changes.

Reply: We thank the reviewer for this suggestion. We replaced the LIF neurons in the encoding stage with ALIF neurons and performed a sensitivity analysis of the baseline threshold θ_{base} and adaptation scale w_θ (Fig. 9, lines 422–429). The analysis shows a clear relationship between encoding parameters, resulting event rates, and decoding accuracy. When firing rates deviate from physiological motor unit activity, performance degrades, whereas an intermediate parameter regime yields stable firing and consistently low RMSE across subjects and tasks. Importantly, a single parameter set generalizes well despite variability in EMG amplitudes, reducing the need for manual threshold tuning compared to fixed-threshold encoding.

However, a residual performance gap between spike-encoded and motor unit–based decoding remains. The observed increase in error is approximately 1 %MVC and is now explicitly contextualized within the broader trade-off between decoding accuracy, memory footprint, and computational cost (lines 440–474, lines 629–641).

Comment 3.5

Robustness covers only dropouts; misses false positives and misattribution. Online MU decomposition can introduce false-positive spikes and cross-MU misattribution. Please simulate both and quantify their effects on error and latency, comparing MU $\rightarrow$ SNN, iEMG $\rightarrow$ LIF $\rightarrow$ SNN, and the baseline.

Reply: We thank the reviewer for raising this important point. We have extended our robustness analysis beyond spike omissions to explicitly include false-positive (spike additions) and cross–motor unit misattribution errors, and quantified their effects on decoding performance. The corresponding results are presented in Figs. 6 and 7 of the main text, with detailed summaries of RMSE, mean absolute error (MAE), and R^2 metrics reported in Supplementary Tables 8, 9, and 10. To complement these quantitative results, we also include representative decoded force traces at selected error levels in Supplementary Figs. 7, 8, and 9. For completeness and consistency, analogous visualizations are also provided for the spike-omission experiment (Supplementary Fig. 6).

These robustness experiments were intentionally applied to the MU→decoder pipelines, as they are designed to emulate decomposition-specific failure modes encountered during online motor unit identification, including missed detections, false positives, and cross–motor

unit misattributions. Such clearly characterized perturbations pertain to the decomposition stage [4] and are therefore relevant for both the MU→SNN and MU→LR baselines. In contrast, the iEMG→LIF→SNN pipeline does not rely on an explicit motor unit decomposition stage and is therefore not directly subject to these decomposition-specific error modes.

Comment 3.6

Minor points
Writing and style consistency (please perform a single, full-manuscript pass):

- *Use "real-time" when adjectival and "in real time" as an adverb; unify "multichannel" (do not mix with "multi-channel");*
- *In text use "Fig. X"; in captions use "Figure X";*

Reply: We thank the reviewer for pointing this out. We carefully revised the manuscript to ensure consistent style throughout. We standardized the use of "real-time" and "in real time," unified "multichannel," and corrected figure references to use "Fig." in the text.

Comment 3.7

Consolidate method details and clarify online/offline boundaries. Provide a single table for LIF threshold strategy (per electrode/participant), target event-rate ranges, and time constants. State explicitly which steps are offline and which are online, with time budgets for each.

Reply: We thank the reviewer for this suggestion. Since the encoding stage now uses ALIF neurons with a single shared parameter set across all subjects and electrodes, a separate table for threshold settings is no longer necessary.
We have clarified the distinction between offline and online components of the pipeline (see lines 852–865). Offline steps include signal preprocessing, motor unit decomposition, parameter selection, and model training. Online components include spike encoding, event-driven SNN inference, and force decoding. Although all analyses in this study were conducted offline for experimental control, the latter stages are designed for real-time operation and were validated on neuromorphic hardware (Section 2.4), including measurement of real-time decoding latency.

Comment 3.8

Data and code sharing. Supply a minimal reproducible package (subset of data, training/inference scripts, and model weights) with dependencies and versioning to reproduce the main figures.

Reply: In the revised manuscript, we have expanded the Data and Code Availability sections to explicitly describe the resources that are now provided (see lines 1074–1078, lines 1079–1082).

Comment 3.9

Reporting Summary:
For transparent evaluation, please ensure the Reporting Summary includes: participant information and inclusion/exclusion criteria; sample-size and power considerations; randomization and blinding; preprocessing; hyperparameter search ranges and surrogate-gradient settings; time-constant and threshold configurations; train/validation/test splits and random seeds; outlier handling and multiple-comparison control.

Reply: We thank the reviewer for this suggestion. The dataset used in this study was previously introduced in [1], where detailed information regarding participant characteristics, and the experimental protocol is provided. To avoid redundancy, we originally referred readers to that work for acquisition-related details. In the revised manuscript, we have expanded the Methods section to clarify hyperparameter search ranges where applicable (for instance in support of the choice of the analysis window, lines 734–736), surrogate-gradient settings (see Table 2), as well as time constants and threshold configurations for the neuron models (lines 801–821, Methods section 4.7). In addition, we now make the full code used to reproduce all analyses publicly available (lines 1079–1082).

Comment 3.10

(Remarks on code availability):
Code and data availability: Based on the materials provided for review, I did not see a code repository, model weights, or a dataset/minimal data subset. The manuscript states that data and code are available from the corresponding author upon reasonable request, which suggests they were not included directly in the review package. If these materials are already hosted elsewhere, a link in the manuscript (or in the response to reviewers) would be very helpful.

Reply: We thank the reviewer for this comment. The decomposed motor unit datasets supporting the main findings have been deposited on Figshare. The Figshare repository is currently set to private to preserve anonymity during peer review; however, access has been provided to the reviewers.

For completeness, the original HD-iEMG recordings have also been made available to the reviewers during the evaluation process. Upon publication, the decomposed motor unit datasets will be made publicly accessible on Figshare, whereas the raw HD-iEMG recordings will remain available upon reasonable request due to ethical and privacy considerations. The Data and Code Availability sections have been updated accordingly (see lines 1074–1078, lines 1079–1082).

Replies to reviewer 4

(Remarks to the author)
This paper proposes SNN decoders to decode motor unit spikes from EMG signals. The SNN decoders can achieve similar performance as the Linear Regression baseline while using a much lower memory footprint. In addition, the authors also show that the two-layer SNN decoder can be more robust to random spike omission compared to the baseline. The proposed work can be used in BCI to perform real-time prediction of finger movements. However, there are several concerns from the reviewer that need to be addressed.

Comment 4.1

Concern 1: The potentially higher computational cost of SNN has not been examined.
*If we consider the binned input for linear regression as B, then each step of linear regression computation will be P=WB, where W is the parameter and P is the output. Now, we consider the input dimension as DB and the output dimension as DP. Then the overall computational cost for linear regression will be DB*DP.*
*Since SNNs do not bin inputs into a single vector, each spike will be integrated into P with a computational cost of DP. Suppose we have sparsity as S and the window size as WIN, then the overall SNN computational cost will be S*WIN*DB*DP. When S*WIN » 1 (e.g., low sparsity or multiple spikes per motor unit within the window), the SNN may require substantially more operations than the baseline, even if it reduces the activation memory.*
Therefore, the authors need to quantify this trade-off. Report the sparsity, FLOPs or synaptic operations, wall-clock time, and energy cost comparisons.

Reply: We thank the reviewer for raising this important point and fully agree that, without explicit measurements of spike sparsity and computational costs across different pipelines, claims regarding the computational efficiency of SNNs remain incomplete.
We have now included detailed measurements of the average computational cost per input sample during inference for both the baseline models and the SNN pipelines (see lines 244–272 and Supplementary Material B). To better isolate the contribution of each processing stage, we further decompose the computational cost by stage.

For the ordinary least-squares linear regression (OLS LR) pipeline, we separately report the cost of input preprocessing (i.e., computing spike counts over temporal bins), measured in accumulate (AC) operations, and the cost of the subsequent matrix multiplication between the regression coefficients and the input matrix, measured in MAC operations. For the SNNs, we report both *dense* (worst-case) and *effective* SynOps to explicitly bracket the computational trade-off under different sparsity assumptions. Dense SynOps correspond to the theoretical worst-case scenario in which all synaptic updates are performed at every time step, as would occur on hardware without support for sparsity. In contrast, effective SynOps account for the actual number of operations executed in the presence of sparse input activity.

To further clarify our computational cost analysis, we detail below the relationship between the reported metrics and the reviewer's formulation. Let D denote the number of motor units, B the number of temporal bins per sample, WIN the number of discrete time steps per sample for the SNN, and $P = 5$ the number of output variables.

For the OLS LR model, the input is represented as a matrix of size $B \times D$, and the regression coefficients have size $D \times P$. The resulting matrix multiplication therefore has a computational cost of

$$\mathrm{MAC}_{\mathrm{LR}} = B \cdot D \cdot P,$$

corresponding to the standard cost of multiplying a $(B \times D)$ matrix with a $(D \times P)$ matrix to obtain a $(B \times P)$ output.

For the SNN, inputs are processed in an event-driven manner and are not aggregated into temporal bins. At each time step, synaptic updates are triggered only by presynaptic spikes. Each presynaptic spike induces an update on all P output neurons, resulting in P AC operations due to the binary nature of the spike. Let S denote the average normalized spike sparsity, defined as the fraction of active input neurons per time step, i.e., $S = \frac{N_{\mathrm{spk}}}{WIN \cdot D}$, where N_{spk} denotes the total number of presynaptic spikes per input sample. Over WIN time steps, the total number of presynaptic spikes is therefore given by $N_{\mathrm{spk}} \approx WIN \cdot S \cdot D$. The resulting effective computational cost associated with only the synaptic updates is then

$$\mathrm{SynOp}_{\mathrm{eff}} = WIN \cdot S \cdot D \cdot P.$$

All operation counts are measured using the Neurobench framework. We additionally report the measured average spike sparsity for each task and subject, together with average and peak wall-clock inference time per sample on CPU (Supplementary Tables 4, 5, 6). In the main text, however, we focus on operation counts, as this metric is independent of the specific hardware used and would allow for a fair comparison across pipelines.

To complement these software-level measurements, we further report direct measurements of inference latency and energy consumption when deploying the SNN decoder on asynchronous neuromorphic hardware (Section 2.4, lines 483–527). On DYNAP-SE, inference is fully event-driven and sparse, resulting in a latency of 35.96 ± 99.81 ms and power consumption on the order of 13 μW during continuous decoding (Section 4.11, Fig. 11, Supplementary Table 11).

Comment 4.2

Concern 2: Unknown overall memory and computational cost reduction on the full pipeline.
The decoder is only the terminal stage of the pipeline. Prior to decoding, raw EMG must be decomposed into motor-unit spike trains, a step the authors note is costly but do not quantify for their experiments. Without end-to-end profiling, it is unclear what fraction of total runtime, FLOPs, energy, and memory is attributable to the decoder versus EMG decomposition. If the decomposition dominates, potential savings from using an SNN decoder may have a negligible impact on overall system efficiency.
Therefore, the authors need to show the overall memory and computational cost reduction on the full pipeline.

Reply: We thank the reviewer for this important point and agree that motor unit decomposition is the dominant contributor to the overall computational and memory cost of the full pipeline. In the revised manuscript, we explicitly quantify the relative contributions of the different stages. For pipelines operating on decomposed motor-unit activity, we separately estimate the costs associated with (i) motor unit decomposition, (ii) preprocessing of spike trains, and (iii) the decoder stage. Detailed calculations are provided in Supplementary Materials A and B, and the resulting trade-offs between accuracy, memory footprint, and computational cost are summarized in Fig. 3.

Our analysis confirms that motor unit decomposition dominates both memory usage and computational cost. The separation matrices must be stored in memory and scale with the number of EMG channels and identified motor units. In addition, the inference-time cost of decomposition scales with the number of motor units, channels, and time steps per sample. As a result, improvements at the decoder level alone have a limited impact on overall system efficiency.

Importantly, this observation actually motivates the spike-encoding pipeline evaluated in this work. By bypassing motor unit decomposition entirely, the spike-based approach enables system-level reductions in memory and computational cost, at the expense of a modest reduction in decoding accuracy (approximately 1%MVC; see lines 629–641).

While end-to-end runtime and energy profiling would be informative, such measurements depend strongly on the specific hardware, operating system, and software framework used for simulation. For this reason, we instead report platform-independent operation counts to enable a fair comparison across pipelines. Latency and energy measurements are provided only where they can be directly measured on the DYNAP-SE neuromorphic hardware (see Section 2.4), thereby avoiding confounding effects from software overhead. Finally, we would like to note that the goal of the present work is not to claim global optimality of SNNs decoders in isolation, but to provide a comparative analysis of decoding backends under two realistic system configurations: one in which motor unit decomposition is required, and one in which it is avoided altogether. We believe this framing, now made explicit in the revised manuscript, provides a more accurate system-level interpretation of the reported efficiency gains.

Comment 4.3

Concern 3: Inadequate sample size and insufficient baseline comparisons
Two subjects cannot support the paper's architectural and methodological conclusions. The Mann-Whitney U tests use n=20 samples from repeated measures on the same two individuals, violating independence assumptions. Claims about SNN reliability (lines 20-22) are unsubstantiated.
In addition, linear regression on binned spike counts is a very naïve baseline method from the ML perspective that deliberately discards temporal information. It will be interesting to see a comparison with more advanced temporal processing methods like RNN-based approaches. There is also a question about the capability of the proposed method. Since it can only achieve similar (or worse) performance than linear regression, it may achieve much lower performance than other, more advanced methods in more complex tasks and settings.

Therefore, the authors can recruit additional participants (minimum n=10) or reframe entirely as a proof-of-concept feasibility study with appropriate statistical methods for repeated measures. The authors can include at least one recurrent baseline to justify claims about temporal processing advantages.

Reply: We thank the reviewer for this comment. We acknowledge that the present study includes a limited number of participants and therefore does not support population-level (i.e generalization) claims. We have explicitly articulated this point in the Discussion (see lines 670–672). Accordingly, we revised the statistical analysis and now report within-subject effect sizes together with their associated 95% confidence intervals, rather than relying on inferential statistical tests (see lines 199–207).

Regarding the baseline models, we agree that linear regression on binned spike counts is a simple approach from a machine-learning perspective; however, it is consistent with prior work on isometric force decoding from decomposed motor units [2, 3]. Given the focus on isometric tasks with slowly varying force profiles, the need for highly expressive temporal models is reduced, as compared to dynamic contraction settings for instance.

Nevertheless, to directly address the reviewer's concern, we added two lightweight recurrent baselines (vanilla RNN and GRU) with hidden size $H = 5$, matched in parameter count to the proposed SNNs models (see lines 208–217). Under these constraints, recurrent models do not yield consistent accuracy improvements over linear regression or SNN-based decoding, while incurring higher computational and memory costs (Fig. 3, lines 218–243, lines 244–272).

Finally, we acknowledge that larger cohorts will be necessary to strengthen statistical power and to assess generalizability across individuals. Furthermore, higher-capacity models may be advantageous in more complex or dynamic task settings than those evaluated here. In particular, tasks involving rapidly varying force trajectories may require richer temporal modeling. These directions are now explicitly discussed as future work (see lines 673–678).

Comment 4.4

Concern 4: Real-time claims not validated.
The manuscript frames around real-time, low-latency processing (Abstract, lines 287-299, Fig. 1c), but all analysis is offline. The "10 ms latency" is a simulation parameter, not measured. Without hardware implementation or benchmarking, the core premise remains speculative. Please check Concern 1 as well.
Therefore, the authors can either implement on neuromorphic hardware with measured latencies or remove real-time advantage claims.

Reply: We agree with the reviewer that real-time and low-latency claims must be supported by measured evidence. In response, we have revised the manuscript to include a hardware-based validation of the proposed decoding pipeline.

Specifically, we implemented the SNN decoder on the Dynamic Neuromorphic Asynchronous Processor (DYNAP-SE), a mixed-signal asynchronous neuromorphic processor, and report direct measurements of inference latency and power consumption during real-time operation (see Sections 2.4, 4.11). The network is deployed on chip with inputs delivered sequentially in real time. We then measured inference latency per sample during on-chip execution, demonstrating that the decoder satisfies the stated real-time constraints when implemented on event-driven hardware. These results replace the previous simulation-based latency estimates and provide a conservative lower-bound assessment of the achievable performance on a general-purpose neuromorphic research platform.

Comment 4.5

Concern 5: The representative of the spike omission and robustness mechanism is unclear.
The authors performed perturbation experiments to show the network's resilience to errors in Section 2.2. However, the experiment is limited to random spike omission. It is not clear how representative random spike omission is of the real-world errors of EMG signals. It feels like this experiment is specifically designed to show the advantage of event-based spike processing. In addition, LIF's noise robustness (Fig. 4) is attributed to thresholding (lines 186-188), but this configuration also has additional layers and parameters. Therefore, additional experiments with different modes of noise and error are preferred to support the claim. Additional ablation studies are also needed on LI with matched depth, LIF with matched parameters, and threshold sensitivity analysis.

Reply: We thank the reviewer for raising this important point. We take this opportunity to clarify our initial rationale for focusing on spike omission (false negatives) in the original robustness analysis. Given the presence of firing thresholds in spiking neurons, subthreshold membrane fluctuations are naturally filtered and do not propagate to downstream layers. We therefore hypothesized that

spike omission would represent a critical failure mode for SNN-based decoding and aimed to explicitly assess its impact. In contrast, for other types of decomposition errors, we did not have a clear prior expectation regarding their effect on decoding performance.

In the revised manuscript, we have extended the robustness evaluation to include additional error modes, namely spike addition (false positives) and cross–motor unit misattribution which randomly re-assigns spikes to neighboring motor units, and have quantified their impact on decoding accuracy in the main text (lines 326–348, and lines 891–897) and Supplementary tables 8, 9, and 10. We also emphasize that these perturbations were introduced exclusively during inference, to emulate realistic errors that may arise in real-time operation due to slight electrode shifts or changes in muscle conditions between training and testing (see lines 594–604).

The results indicate that false-positive errors generally have a more limited impact than missed detections for both the baseline and the SNNs. In particular, random spike additions, especially at high error levels, tend to occur during transient phases of the force profile and lead to temporary force overestimation. However, because the steady hold phase dominates the overall prediction, the resulting degradation in performance remains moderate. Cross–motor unit misattributions exhibit an even smaller effect, as they preserve the temporal structure of the spike trains and therefore minimally disrupt the decoded force trajectories.

Finally, we note that exact depth matching across pipelines is not straightforward, as the architectures serve different computational roles. In particular, the spiking configuration requires an additional layer to convert discrete spike events into a continuous output representation, which has no direct analogue in the leaky or linear baseline. As such, strict depth matching would modify the functional structure of the models rather than ensure a fair comparison. For this reason, additional ablation studies on threshold parameters and matched-depth variants were not included. The experiments in Section 2.2 were specifically designed to evaluate inference-time perturbations on already trained and systematically compared pipelines (SNN+LI, SNN+LIF, and OLS LR), therefore isolating robustness under the respective optimal training conditions for each configuration.

Comment 4.6

Concern 6: Hyperparameter justification is lacking.

- *80ms window: sweep results not shown (lines 403-405)*
- *Time constants (30ms, 80ms): no justification provided (lines 459, 462)*
- *Encoding thresholds: inconsistent between subjects, suggesting poor generalizability*
- *Architecture depth: no exploration reported*

Therefore, the authors need a systematic hyperparameter analysis and sensitivity studies.

Reply: We have revised the manuscript to motivate the selected values.

We added the sweep analysis results (see Supplementary Fig 1, lines 734–736). To address inconsistent encoding thresholds across subjects, we replaced fixed-threshold encoders with adaptive leaky integrate-and-fire (ALIF) neurons. We provide threshold–event-rate–accuracy curves and sensitivity analysis showing stable performance across subjects using a single parameter set (see Fig. 9, lines 422–429). Finally, we clarified the choice of the architecture depth, and SNN hyperparameters (see lines 776–779, and lines 801–821).

Replies to reviewer 5

(Remarks to author)
I co-reviewed this manuscript with one of the reviewers who provided the listed reports. This is part of the Nature Communications initiative to facilitate training in peer review and to provide appropriate recognition for Early Career Researchers who co-review manuscripts.

References

[1] A. Grison et al. *Intramuscular High-Density Micro-Electrode Arrays Enable High-Precision Decoding and Mapping of Spinal Motor Neurons to Reveal Hand Control*. 2024. DOI: 10.48550/arXiv.2410.11016.

[2] F. Xu, Y. Zheng, and X. Hu. "Estimation of Joint Kinematics and Fingertip Forces using Motoneuron Firing Activities: A Preliminary Report". In: *2021 10th International IEEE/EMBS Conference on Neural Engineering (NER)*. 2021, pp. 1035–1038. DOI: 10.1109/NER49283.2021.9441433.

[3] M. Xia et al. "Simultaneous and proportional control of wrist and finger force via motor unit activity". In: *Biomedical Signal Processing and Control* (2025), p. 107399. DOI: 10.1016/j.bspc.2024.107399.

[4] J. C. Kline and C. J. De Luca. "Error reduction in EMG signal decomposition". In: *Journal of Neurophysiology* (2014), pp. 2718–2728. DOI: 10.1152/jn.00724.2013.

Revision 2 - Response to Reviewer 4 for the manuscript “Spiking Neural Network Decoders of Finger Forces from High-Density Intramuscular Microelectrode Arrays”

1. Line 60-63 on the additional memory cost of LR is not really a good motivation anymore, since the motor unit decomposition dominates the memory cost if they are in the same system. In Figure 1, the authors show the possibility of having two systems; maybe the author needs to make it clearer why two systems are better.

We thank the reviewer for this suggestion. We agree that emphasizing the memory overhead associated with spike buffering is not precise when motor unit decomposition is part of the pipeline, as it dominates the overall memory cost. We have revised the sentence to instead focus on the representational mismatch, which provides a clearer motivation for integrating SNNs with motor units.

2. I agree with the authors that for a real-time low-cost system, a solution not using motor unit decomposition is preferred. However, the current performance of the SNN without motor units is still far from that of the one with them. It will be interesting to see what results LR and RNN will get for the same input data without motor unit decomposition. Will SNN do better both in cost and accuracy?

We appreciate the reviewer’s suggestion and agree that comparing LR and RNN models on the same input data without motor unit decomposition would be informative. In this work, our focus was on addressing the representational mismatch and building on the strong performance observed with motor unit-based decoding. Extending the comparison to conventional models under the same non-decomposed input representation would be a natural next step.
